# Healing from the Ocean: Targeting Shared Mechanisms in Autism and Epilepsy Using Algae-Derived Compounds

**DOI:** 10.3390/md24080277

**Published:** 2026-08-10

**Authors:** Dorit Avni, Orly Weissberg, Noam Pintel, Liat Izraelov

**Affiliations:** 1 Translational Biocompounds Research Group, MIGAL-Galilee Research Institute, Kiryat Shmona 1101600, Israel; 2 The Department of Biotechnology, Tel Hai University, Kiryat Shmona 1220800, Israel

**Keywords:** autism spectrum disorder, epilepsy, marine algae, inflammation, oxidative stress, gut–brain axis, bioactive compounds, microbiome, neurofunction

## Abstract

Autism spectrum disorder (ASD) and epilepsy are complex, frequently co-occurring neurodevelopmental and neurological disorders that share key mechanisms, such as altered neurotransmission, oxidative stress, neuroinflammation, and gut–brain axis disruption. Despite pharmacological advances, current treatments often provide only partial relief and are associated with significant side effects. The comorbidity of ASD and epilepsy, affecting millions worldwide, remains under-recognised and poorly addressed, imposing a profound burden on patients, families, and healthcare systems through reduced quality of life, increased caregiving demands, and substantial social and economic costs. This review highlights the convergent pathways shared between ASD and epilepsy, including immune dysregulation, synaptic dysfunction, and metabolic imbalance, which create opportunities for unified therapeutic strategies. Marine algae have emerged as a sustainable source of bioactive compounds offering a unique potential to address these overlapping pathologies. Algal polyunsaturated fatty acids, carotenoids, polyphenols, polysaccharides, and vitamins have antioxidant, anti-inflammatory, neuroprotective, and microbiota-modulating activities. By addressing both the biological underpinnings and clinical burden of ASD–epilepsy comorbidity, algae-based strategies represent a novel and ecologically sustainable direction for mitigating ASD–epilepsy comorbidity and advancing marine-inspired neurotherapeutics.

## 1. Introduction

### 1.1. Autism Spectrum Disorder (ASD)

ASD is a complex neurodevelopmental condition that has gained increasing attention owing to its increasing prevalence and impact. It is characterized by impaired social communication, language deficits, and repetitive behaviors [1,2,3]. In 2020, ASD was found to affect approximately 1 in 36 8-year-old children in the United States [4,5]. Currently, the only FDA-approved medications for ASD-related symptoms are risperidone and aripiprazole, which help to control irritability and aggression. However, there are no approved medications for the core symptoms of ASD such as social communication difficulties and repetitive behaviors. Other medications, such as psychotropic drugs, are often used to treat co-occurring conditions such as anxiety or Attention Deficit Hyperactivity Disorder (ADHD). However, they are not specifically approved for ASD [6,7]. These limited treatment options highlight the need for novel interventions, such as nutritional therapeutics.

The exact cause of ASD remains unclear; however, evidence suggests that both genetic and environmental factors contribute to its development [1,8]. Numerous genes are associated with ASD, highlighting their strong genetic components. In addition, environmental factors such as prenatal infections, air pollution, advanced parental age, and maternal diet have also been implicated in increasing the risks [9,10]. Recent studies have highlighted the significant roles of neuroinflammation and oxidative stress in the pathophysiology of ASD. It was found that maternal immune activation, in particular, can lead to inflammation and oxidative stress, contributing to brain abnormalities and ASD development [11,12,13,14].

Neuroinflammation is recognised as a central driver of ASD pathology. Increased microglial activation and elevated pro-inflammatory cytokines, including IL-6, IL-1β, TNF-α, and IFN-γ, have been reported in multiple brain regions of individuals with ASD, particularly in the cortex, amygdala, and hippocampus, which are critical for social and cognitive function [15,16,17]. Cerebrospinal fluid analyses further demonstrate elevated inflammatory mediators in ASD, alongside associations between chronic low-grade inflammation and behavioral impairments [18,19]. Activated microglia and heightened cytokine signaling are now considered characteristic features of ASD brain pathology [20]. These immune alterations correlate with broader inflammatory and chemokine activation, as well as changes in peripheral and central immune cell populations observed in both human studies and animal models [21]. In addition, oxidative stress and increased reactive oxygen species contribute to ASD-related neurobiological dysfunction [22].

Maternal immune activation during pregnancy has been linked to ASD risk in offspring [23]. Experimental models, such as maternal immune activation induced by lipopolysaccharide (LPS), have shown that offspring exposed to this condition exhibit autistic-like behaviors, including reduced social interactions and repetitive behaviors. These models also revealed impaired fetal brain development, significant changes in gut microbiota composition, and increased levels of inflammation markers linked to ASD pathogenesis [13,24,25]. Clinical evidence further supports this association, as women who were hospitalised due to infections during pregnancy and early life infections in infants have both been correlated with a higher incidence of ASD [26,27].

The gut–brain axis plays a crucial role in ASD-related symptoms. Children with ASD often exhibit distinct gut microbiota profiles compared with typical children, which is linked to the restricted dietary habits of children with ASD. These have been found to influence brain function and behavior [25,28,29,30]. Studies have found that children with ASD frequently experience gastrointestinal (GI) comorbidities. In 2023, the prevalence of GI symptoms in children with ASD was estimated to be approximately 20% higher than that observed in the general pediatric population [29,30]. These symptoms include constipation, diarrhea, bloating, abdominal pain, acid reflux, vomiting, excessive gas, foul-smelling stool, and food allergies [31,32]. It was found that children with ASD have approximately a 56% *Bacteroidetes* to *Firmicutes* ratio compared to typical children, with behavioral effects in these children. These findings strengthen the association between ASD and gut microbiome [33,34,35,36,37]. Certain gut bacteria produce short-chain fatty acids (SCFAs) that can modulate brain activity and behavior. Additionally, they regulate the serotonin system, an essential neurotransmitter in the gut–brain axis [38]. Increased intestinal permeability, often called “leaky gut”, has been observed in some individuals with ASD, potentially allowing harmful substances to impact the brain [39]. The gut microbiota significantly influences the production of neurotransmitters. Research indicates that gut bacteria produce neurotransmitters, such as dopamine, norepinephrine, serotonin, and gamma-aminobutyric acid (GABA), which influence brain function and behavior [40,41], and emphasises the importance of the gut–brain axis in ASD. Given the prebiotic and anti-inflammatory properties of algae, their role in modulating gut–brain dysfunction warrants examination.

Epilepsy is one of the most common comorbidities in individuals with ASD, with studies indicating a substantial overlap in neurodevelopmental pathways. Epidemiological data suggest that epilepsy affects 6–27% of individuals with ASD, with a prevalence rising to 46% among those with intellectual disability. Conversely, epilepsy is associated with an elevated risk of ASD, with comorbidity rates of up to 37% [42,43]. While the risk factors for developing epilepsy in individuals with ASD remain unclear, the proposed leading hypotheses include genetic factors, excitatory/inhibitory imbalance, and structural brain abnormalities [43]. Studies suggest that children with both ASD and epilepsy may present a distinct clinical phenotype, often marked by an earlier onset of ASD symptoms, increased repetitive behavior, unusual sensory interests, and significant gross motor coordination impairments [43]. Compared to children with ASD alone, those with ASD and epilepsy experience more severe cognitive and language impairments, with epileptic seizures worsening these difficulties [42,43,44,45]. Electroencephalography (EEG) abnormalities, particularly epileptiform discharges, are frequently observed in children with ASD and have been linked to autistic behaviors even in the absence of clinically diagnosed epilepsy, showing a significant correlation with ASD symptoms [46,47,48]. This comorbidity is associated with disrupted synaptic plasticity and an excitatory–inhibitory imbalance, resulting in cortical hyperexcitability and increased seizure susceptibility. Structural and neurochemical abnormalities, such as neocortical minicolumn pathology and impaired gamma-aminobutyric acid-ergic (GABAergic) signaling, further support shared neurodevelopmental mechanisms [49,50].

Furthermore, abnormalities in genes regulating GABAergic, such as SH3 and multiple ankyrin repeat domains protein 3, Contactin-associated protein-like 2, Sodium voltage-gated channel alpha subunit 1, Neuroligin 4, X-linked, and Phosphatase and tensin homolog (PTEN) have been identified in individuals with both ASD and epilepsy, suggesting a shared genetic basis between the two conditions [46,47,48,49,51,52]. PTEN mutations, for example, are associated with a high prevalence of ASD (51%) and epilepsy (15%), and are often accompanied by structural brain abnormalities and impaired myelination [52]. Neurotransmission has been linked to dysregulated neuronal activity that leads to seizures and ASD-related symptoms [53,54,55]. Neuroinflammation, glial cell dysfunction, and dysregulated cytokine signaling play critical roles in the pathophysiology of both epilepsy and ASD, contributing to neuronal hyperexcitability and synaptic dysfunction. Excessive activation of inflammatory pathways, particularly in microglia, leads to oxidative stress, impaired autophagy, persistent neuroimmune activation, exacerbating seizures, and ASD-related symptoms. Targeting neuroinflammation through anti-inflammatory therapies has shown potential in mitigating both seizure susceptibility and ASD-associated behavioral impairments, highlighting its relevance as a therapeutic avenue [53,56,57,58].

### 1.2. Epilepsy

Epilepsy is a neurological disorder characterised by recurrent unprovoked seizures resulting from abnormal electrical activity in the brain [59]. It is diagnosed after two unprovoked seizures or when a brain condition indicates a high risk of further seizures [60]. Epilepsy is one of the most prevalent neurological disorders, affecting approximately 1% of the global population [61]. The International League Against Epilepsy classification system provides a structured framework for epilepsy diagnosis and management, categorising epilepsy into seizure and epilepsy types. “Seizure type” refers to the individual characteristics of a seizure, whereas “epilepsy type” describes the overall seizure pattern observed in a patient [62]. The diagnosis of epilepsy relies on a combination of clinical history assessment, EEG to detect abnormal neuronal activity, and MRI to identify structural brain abnormalities. However, innovative approaches include genomic testing for epilepsy-related mutations, advanced neuroimaging techniques for enhanced structural and functional brain assessments, and artificial intelligence-based models for seizure prediction and risk assessment [63,64].

Epilepsy can arise from a wide range of factors, including genetic mutations, structural abnormalities in the brain, metabolic dysfunction, immune dysregulation, and infections [65]. An imbalance between excitatory and inhibitory neuronal activity, as well as ion channel dysfunction, plays a crucial role in the pathophysiology of epilepsy [54,66,67]. Neuroinflammation is increasingly recognized as a contributor to epileptogenesis. Microglial activation and elevated pro-inflammatory signaling promote neuronal hyperexcitability and seizure susceptibility [68,69,70]. Inflammatory cascades, including NF-κB– and mTOR-mediated pathways, also interact with blood–brain barrier integrity and synaptic stability [71,72], reinforcing mechanistic overlap with ASD.

The primary treatment for epilepsy involves antiepileptic drugs (AEDs), which modulate neuronal excitability and suppress seizure activity. Common AEDs include phenytoin, carbamazepine, valproate, lamotrigine, and levetiracetam, all of which target ion channels and neurotransmitter systems to stabilise neuronal firing [73,74]. Despite the availability of these pharmacological options, 30–40% of patients experience drug-resistant seizures, necessitating alternative therapeutic approaches [75,76]. For patients with drug-resistant epilepsy, surgical interventions such as the removal of the seizure-causing brain area can be considered. However, not all patients are suitable candidates because of surgical risks and potential complications. In addition, neurostimulation therapies, including deep brain stimulation, vagus nerve stimulation, and responsive neurostimulation, provide alternative mechanisms for seizure control by modulating brain activity to prevent seizures; however, their effectiveness varies among individuals [77]. Dietary modifications such as the ketogenic diet have also demonstrated efficacy in reducing seizure frequency in some individuals; however, maintaining such diets can be challenging and may lead to side effects [75,78]. Although many patients benefit from current treatments, drug resistance remains a challenge, highlighting the need for new therapies.

The gut–brain axis is increasingly implicated in epilepsy. Dysbiosis, altered neurotransmitter regulation, increased intestinal permeability, and microbiota-derived metabolites have been associated with seizure susceptibility and drug resistance [79,80,81,82,83,84,85,86,87,88,89,90,91,92,93,94]. Modulation of microbial composition, including through dietary or probiotic interventions, may influence neuronal excitability and inflammatory tone [79,86,87,88,89]. These findings support the contribution of gut–brain axis disruption to epileptogenesis while highlighting the need for mechanistically guided clinical validation. Taken together, these findings highlight the interconnected roles of immune activation, oxidative stress, and gut–brain axis disruption in ASD and epilepsy, underscoring the need to examine their shared mechanisms as a foundation for innovative therapies.

### 1.3. The Shared Mechanisms Between ADS and Epilepsy

The overlap between ASD and epilepsy extends beyond clinical comorbidity and reflects convergent biological vulnerabilities. Both disorders exhibit chronic neuroinflammation, oxidative imbalance, excitatory–inhibitory dysregulation, and gut–brain axis disruption [8,95,96,97,98]. Dysregulation of inflammatory cascades, including mTOR- and COX-2-associated pathways, further contributes to seizure susceptibility and neurodevelopmental dysfunction [53]. Disruptions in the gut–brain axis and alterations in the microbiome reinforce these interactions, promoting systemic immune activation and central neuroinflammation [95,96,97,98,99,100]. Together, these interconnected processes form a shared mechanistic framework underlying ASD–epilepsy comorbidity.

Treating epilepsy in individuals with ASD is challenging because of the high variability in response to AEDs. Certain AEDs may increase repetitive behaviors or cognitive dysfunction [101]. Additionally, existing treatments often rely on generalised epilepsy approaches that do not address the unique characteristics of ASD [49]. Given the high prevalence of comorbid ASD and epilepsy, there is an increased need to develop targeted pharmacological strategies that address their shared pathophysiological mechanisms, minimise associated comorbidities, and enable early multidisciplinary intervention [102]. Symptom variability and potential drug interactions further complicate the selection of safe and effective therapies [103]. The increasing complexity of treating epilepsy and ASD necessitates the exploration of novel, well-tolerated therapeutic avenues, including plant-based therapies, to achieve more personalised and effective treatment outcomes. Among these promising natural sources, algae present a compelling option, with diverse bioactive compounds that exhibit neuroprotective, anti-inflammatory, and antioxidant properties [104,105,106,107,108,109].

### 1.4. Mechanistic Framework of Algae-Derived Compounds in ASD–Epilepsy Comorbidity

Algae-derived bioactive compounds exert their potential therapeutic effects in ASD–epilepsy comorbidity through modulation of a limited set of interconnected molecular and cellular pathways. Despite their structural diversity, polysaccharides, carotenoids, polyunsaturated fatty acids, polyphenols, and vitamins converge on four principal mechanistic domains: neuroinflammation, oxidative stress imbalance, synaptic and excitatory–inhibitory dysregulation, and gut–brain axis disruption. These processes are biologically interdependent. Chronic activation of inflammatory pathways enhances oxidative damage, which in turn alters neuronal excitability and synaptic plasticity, while microbiota-driven immune signaling further amplifies central neuroinflammation and blood–brain barrier dysfunction.

At the molecular level, algae-derived compounds commonly target key signaling cascades implicated in both ASD and epilepsy, including NF-κB, JAK/STAT, mTOR, PI3K/Akt/GSK3β, TLR4/NLRP3, and the Nrf2 antioxidant pathway. Polysaccharides and polyphenols frequently suppress pro-inflammatory cytokine production and microglial activation, carotenoids enhance antioxidant defenses and modulate inflammasome activity, PUFAs regulate membrane excitability and ion channel function, and vitamins support mitochondrial metabolism and neurotransmitter synthesis. Additionally, several compound classes modulate microbiota–gut–brain signaling pathways, contributing to immune and neurochemical regulation.

**Figure 1:**
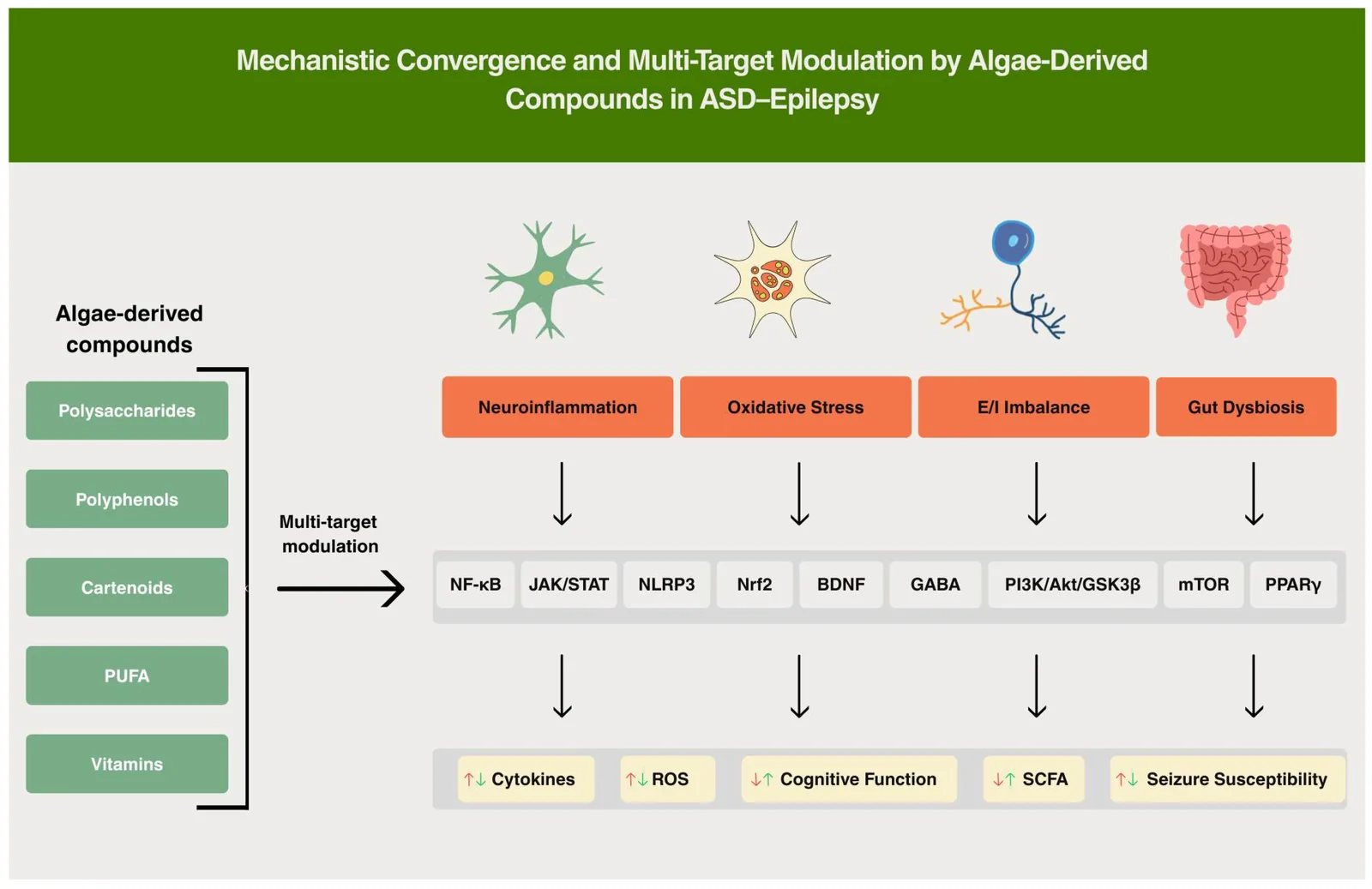
Mechanistic convergence between ASD and epilepsy and multi-target modulation by algae-derived compounds. ASD and epilepsy share interconnected mechanisms, including neuroinflammation, oxidative stress, excitatory/inhibitory imbalance, and gut dysbiosis, converging on pathways such as NF-κB, JAK/STAT, NLRP3, Nrf2, PI3K/Akt/GSK3β, mTOR, PPARγ, BDNF, and GABA signaling. Algae-derived compounds (polysaccharides, polyphenols, carotenoids, PUFAs, and vitamins) modulate these pathways, contributing to reduced inflammatory and oxidative burden, improved neurotrophic and synaptic balance, enhanced SCFA production, and decreased seizure susceptibility. Red arrows indicate pathological alterations, and Green arrows indicate modulatory effects of marine bioactives. Schematic diagram was created with Canva.

This mechanistic convergence provides a coherent biological framework for considering algae-derived compounds as multi-target modulators of ASD–epilepsy pathology rather than single-pathway agents. The integrated signaling interactions underlying these effects are summarized in Figure 1 and Table 1.

## 2. The Potential Effect of Algae-Based Bioactive Compounds on ASD and Epilepsy

Comprising nearly 70% of the Earth’s surface, algae are an essential group of organisms that thrive in diverse ocean ecosystems. Given their ability to produce a diverse range of bioactive compounds, these organisms have garnered significant scientific attention. Algae are an important source of compounds with diverse biological functions [110,111]. Currently, more than 200,000 eukaryotic marine species have been confirmed, of which approximately 44,000 are algae [112,113]. Algae thrive in freshwater and marine ecosystems, and are autotrophic organisms that can be unicellular or multicellular, producing their own food through photosynthesis [114,115]. Algae absorb carbon dioxide (CO_2_) during photosynthesis and, in combination with water and mineral salts, form complex organic molecules and release oxygen (O_2_). Algae vary significantly in size, morphology, and structural complexity; therefore, they are divided into two main groups: microalgae and macroalgae [116].

Macroalgae are photosynthetic eukaryotes that play a fundamental role in aquatic ecosystems. Although classification systems vary depending on the criteria used, the most widely accepted approach categorizes macroalgae into three major groups based on their dominant pigments: brown algae (Ochrophyta), red algae (Rhodophyceae), and green algae (Chlorophyceae) [113,117,118]. Green algae are commonly associated with freshwater environments, whereas brown and red algae are predominantly found in marine habitats, with red algae particularly abundant in deeper or tropical marine waters [119]. Their nutritional components include proteins (5–47%), lipids (1–5%), minerals (7–36%), and polysaccharides (15–76%) [112,120,121]. They are rich in micronutrients, such as vitamins and secondary metabolites, which are mostly antioxidants, including pigments and polyphenols [113].

Microalgae are both prokaryotic and eukaryotic photosynthetic organisms. Compared to higher plants, they constitute a diverse group of organisms with relatively simple cellular structures [122]. Microalgae are highly varied, with estimates ranging from 200,000 to 800,000 species in natural environments [123]. They range in size from approximately 0.2–200 µm, are highly efficient at accumulating carbon, and make an essential contribution to efforts to combat climate change by absorbing carbon dioxide [124,125,126]. Microalgae are composed of bioactive compounds, including polyphenols, phytosterols, pigments, mycosporine-like amino acids, proteins, lipids, carbohydrates, vitamins, and antioxidants [127].

Both macroalgae and microalgae are recognized as valuable sources of bioactive compounds with significant benefits to human health. These organisms are rich in a wide array of essential nutrients, including proteins, polysaccharides, lipids, PUFAs, flavonoids, vitamins, minerals, amino acids, and carotenoids [113,128,129], many of which exhibit antioxidant, anti-inflammatory, antimicrobial, and other therapeutic activities [113], making them promising candidates for nutraceutical applications. To provide structural context for the discussed compound classes, representative chemical structures are shown in Table 2.

### 2.1. Polysaccharides

Polysaccharides are long-chain carbohydrates composed of monosaccharide units that are linked by glycosidic bonds. They perform important structural, storage, and metabolic functions in living organisms [130]. Marine algae are an essential source of polysaccharides, especially sulfated types, including fucoidan, carrageenan, and alginate [131]. These algal polysaccharides exhibit diverse biological activities with the most prominent anti-inflammatory effects [132,133,134]. Compounds such as ulvan from green algae, fucoidan from brown algae, and carrageenan and sulfated galactans from red algae exhibit a wide range of therapeutic characteristics due to variations in branching patterns, degree of sulfation, monosaccharide composition, and molecular weight [135]. Their biological activities include lipid reduction and anti-viral, immunomodulatory, and anti-cancer effects [136,137]. Furthermore, algal polysaccharides have demonstrated notable antioxidant activity both in vitro and in vivo, as evidenced by their ability to reduce oxidative stress markers in hyperlipidemic animal models [138]. Additionally, their prebiotic effects have been linked to improved gut microbiota composition and benefits in models of inflammatory bowel disease [139]. Recent advancements have expanded the application of polysaccharides in biomedical fields, where they are incorporated into hydrogels, nanoparticles, and scaffolds for drug delivery, wound healing, and tissue engineering [140]. These advances underscore the therapeutic versatility of algal polysaccharides and highlight their potential for integration into pharmaceutical and nutraceutical applications.

#### 2.1.1. The Potential Effect of Polysaccharides on ASD

Polysaccharides are promising compounds for managing ASD, addressing its core symptoms by supporting gut health and reducing inflammation and oxidative stress in the brain and body. They play a role in regulating the gut–brain axis, which is linked to ASD [141,142]. Jang et al. suggested that metabolites and bioactive compounds produced by probiotics, including polysaccharides, may help manage neurological disorders, such as ASD, by influencing the gut–brain axis. However, further research is necessary to confirm these potential benefits and fully understand their therapeutic impact [143].

Polysaccharides, specifically heparan sulfate linked to the glypicans, are crucial for the formation of proper synapses and neural communication by regulating guidance signals that ensure accurate neuronal connectivity. When these signals are disrupted, synapse formation may be weakened or misdirected, potentially contributing to abnormal brain development and behaviors commonly observed in ASD [144,145,146]. In support of their neurodevelopmental relevance, polysaccharides derived from *Paris polyphylla* have been shown to improve learning and memory in the offspring of aging pregnant mice via the Wnt/β-catenin signaling pathway [147]. Beyond synaptic development, polysaccharides also exert neuroprotective effects in rodent models by reducing oxidative stress, further supporting their potential in ASD management [141]. β-Glucan has shown improvements in behavioral scores (CARS) and beneficial modulation of gut microbiota composition, indicating its potential as a therapeutic agent targeting the gut–brain axis in children with ASD [148,149,150].

While Kim et al. suggested that an aqueous extract of *Sargassum horneri*, rich in polysaccharides, increased the levels of IL-2, IFN-γ, and TNF-α in immunosuppressed mice [151], other studies indicate that algae-derived polysaccharides more commonly exhibit anti-inflammatory effects. Specifically, reductions in pro-inflammatory cytokines and inhibition of Nuclear Factor kappa-light-chain-enhancer of activated B cells (NF-κB) and Mitogen-Activated Protein Kinase (MAPK) signaling pathways have been observed [152,153,154]. Fucoidan from the brown alga *Saccharina japonica* demonstrated strong anti-inflammatory activity in RAW 264.7 macrophages. The cells showed inhibition of TNF-α, IL-1β, and IL-6 secretion as well as the NF-κB, MAPK, and JAK-STAT pathways [155]. Additionally, fucoidans from brown algae have been shown to regulate Toll-like receptor (TLR) 3 activity and inhibit cytokines (IL-1α, IL-1β, TNFα, and IL-6) and chemokines (C-C motif chemokine ligand (CCL)-5, CCL22, CXCL1, CXCL5, and CXCL8) [156], as well as COX-2 and Inducible Nitric Oxide Synthase (iNOS) [157]. Beyond immunomodulation, fucoidan isolated from *Sargassum fusiforme* has been shown to improve cognitive function, including spatial learning and memory, in mice [158]. Supporting these findings, Santos et al. recently highlighted the immunomodulatory potential of marine-derived polysaccharides in ASD-related immune dysregulation. Of the 38 ASD-associated inflammatory mediators, 21 were influenced by marine-derived molecules, with *Sargassum* and other macroalgae showing particularly strong effects on cytokine regulation and neuro-inflammation. Further studies using ASD models are warranted to confirm their therapeutic potential [159].

Polysaccharides may further influence ASD-related neurobiology through modulation of the microbiota–gut–brain axis. β-Glucan has been shown to enhance gut barrier integrity, modulate immune signaling, and promote beneficial shifts in microbial composition [160]. Additional studies report increased brain-derived neurotrophic factor (BDNF) expression, reduced pro-inflammatory cytokines, and improved behavioral outcomes following β-glucan or prebiotic supplementation [161,162]. These microbiota-mediated and immunomodulatory effects are consistent with broader findings demonstrating anti- [163,164,165,166,167,168,169,170,171]. Together, these findings indicate that supplementation with algae-based β-glucan and polysaccharides may affect neurobehavioral outcomes relevant to ASD spectrum conditions via the microbiota–gut–brain axis.

#### 2.1.2. The Potential Effect of Polysaccharides on Epilepsy

Over 20 antiepileptic drugs have been developed in the past few decades; however, drug-resistant epilepsy remains a significant clinical challenge, emphasising the urgent need for novel therapeutic approaches [172]. One emerging target is oxidative stress, which plays a key role in epilepsy pathogenesis owing to the brain’s high oxygen demand, abundance of unsaturated fatty acids, and relatively weak antioxidant defenses [173]. A systematic review that examined papers published between 2013 and 2023 found potential benefits of dietary polysaccharides in managing central nervous system disorders, including epilepsy, by reducing oxidative stress and inflammation, protecting neurons, and regulating ion channels and mechanisms associated with decreased seizure frequency and severity [141]. Polysaccharides exert antiepileptic effects via multiple mechanisms. They modulate inflammation by reducing pro-inflammatory cytokines, balancing neurotransmitters such as GABA and glutamate, and regulating ion channels critical for neuronal excitability [166,172,174,175].

Additionally, polysaccharides enhance gut health by influencing brain function through the gut–brain axis, which has been increasingly implicated in epilepsy [176]. Elevated glycosaminoglycan levels in patients with epilepsy further suggest involvement of polysaccharide-related pathways in disease pathophysiology [177]. β-Glucans from diverse sources, such as barley, have demonstrated neuroprotective and anti-inflammatory effects, while supplementation with derived from the microalga *Euglena gracilis* alleviated diet-induced cognitive impairment in middle-aged mice, highlighting the relevance of algal polysaccharides in gut–brain axis modulation and neuronal support [161,178].

Polysaccharides have long been used in traditional Chinese medicine due to their neuroprotective and antiepileptic properties. Compounds derived from *Ganoderma lucidum*, *Gastrodia elata*, *Artemisia* species, and *Huperzia serrata* have demonstrated efficacy in reducing oxidative stress, neuroinflammation, and seizure severity in preclinical epilepsy models [132,133,134,179,180,181,182]. *Astragalus* and *Bupleurum chinense* polysaccharides have been shown to improve cognitive function and neuronal survival by modulating pathways including TLR4/NF-κB and Phosphoinositide 3-kinase/Protein kinase B (Akt)/glycogen synthase kinase-3 beta (PI3K/Akt/GSK-3β) pathways [173,183].

Similar to these findings from terrestrial sources, algal bioactives are increasingly being recognised as potent modulators of intracellular signaling pathways implicated in neuroinflammation and neuronal survival, notably the TLR4/NF-κB and PI3K/Akt/GSK-3β cascades. Overactivation of these pathways promotes microglial activation, cytokine release, and impaired synaptic plasticity, processes strongly associated with seizure susceptibility and ASD-related neurodevelopmental alterations [144,184]. In parallel, dysregulation of the PI3K/Akt/GSK-3β pathway contributes to impaired neurogenesis, abnormal synaptic plasticity, and epileptogenesis, while pharmacological inhibition of GSK-3β has demonstrated anticonvulsant and pro-cognitive effects [142,185,186,187].

Algal-derived polysaccharides (e.g., as fucoidan and ulvan) have been shown to inhibit TLR4 expression and NF-κB nuclear translocation, thereby reducing neuroinflammation [142,188]. Collectively, these findings highlight the therapeutic potential of natural polysaccharides in ASD and epilepsy via multitargeted neuroprotective mechanisms.

### 2.2. Carotenoids

Carotenoids are among the most prevalent lipid-soluble pigments in nature, are produced by algae, plants, and some microorganisms, and are of growing biomedical interest. They contribute to cellular protection by reducing oxidative damage and regulating the immune system [189]. Algae are currently the subject of intense research on the synthesis of carotenoids due to their wide range of biological activities and their applications in food, medicine, and cosmetics [190,191,192,193].

Algal carotenoids, including β-carotene, astaxanthin, lutein, and fucoxanthin, are of considerable interest because of their potent antioxidant activity and their potential to reduce the risk of chronic diseases, including cancer and cardiovascular disorders [194]. Large amounts of provitamin A, such as lutein, α-carotene, β-carotene, violaxanthin, neoxanthin, and fucoxanthin, are produced by *Spirulina*, *Chlorella*, *Dunaliella*, and *Haematococcus* [195]. β-Carotene, a common pigment involved in the photosynthetic process of microalgae, has demonstrated a range of bioactivities [196]. As a micronutrient for human health, it is the most prevalent dietary provitamin A carotenoid that can be converted to vitamin A [197]. Studies have shown the biological effects of crude β-carotene extract and pure substances from microalgae, including hepatoprotective, anti-obesity, anti-inflammatory, immunomodulatory, and anti-cancer properties [198,199,200,201,202]. Beta-carotenoids cannot be synthesised by the human body and must be obtained through the diet [203]. Most of them can efficiently cross the BBB, and higher dietary intake is positively associated with increased brain levels of beta-carotenoids [204,205,206,207,208].

#### 2.2.1. The Potential Effect of Carotenoids on ASD

Lutein, a carotenoid with potent antioxidant properties, has attracted attention for its potential neuroprotective effects in neurodevelopmental disorders, including ASD. To evaluate the neuroprotective potential of lutein, lutein-loaded nanoparticles were studied in a rat model of ASD induced by prenatal exposure to valproic acid. Behavioral assessments showed that lutein improved social preference and memory and reduced repetitive behavior and anxiety. Biochemical tests revealed decreased levels of oxidative stress markers and apoptosis in the hippocampus and prefrontal cortex, accompanied by improved neural structural integrity [209]. Although this study did not directly measure synaptic markers or inflammatory cytokines, the biochemical and behavioral effects of lutein imply that it may help manage neuroinflammation, oxidative balance, and synaptic function in ASD-related symptoms. Complementary evidence was provided by a Drosophila model exposed to the insecticide imidacloprid, which simulated behavioral features associated with ASD and ADHD. The treatment resulted in improvements in behavioral abnormalities including hyperactivity, aggression, and impaired social interactions. Additionally, it protects oxidative stress markers and preserves the expression of proteins involved in normal neuronal functions [210]. In a D-galactose-induced aging model, dietary supplementation with carotenoids, polyphenols, and omega-3 fatty acids improved hippocampus-dependent short-term memory and enhanced antioxidant defenses and neurogenesis markers in the brain while also increasing the abundance of beneficial gut taxa [211].

Food-derived bioactive compounds, such as carotenoids, modulate the gut microbiota composition and influence the production of metabolites, including neurotransmitters. Microbial metabolites, such as GABA, acetylcholine, serotonin, and dopamine, play pivotal roles in the gut–brain axis, affecting neurological functions and potentially impacting mental health conditions such as depression and anxiety [212]. β-carotene reduced pro-inflammatory genera (*Prevotella*, *Blautia*) and enriched the anti-inflammatory genus Parabacteroides, thereby improving intestinal morphology and reducing systemic inflammation [213]. Carotenoids may also influence gut microbiota by modulating oxidative stress, enhancing mucosal immunity (e.g., IgA-related effects), and potentially having bactericidal properties. Owing to their limited absorption in the small intestine, they reach the colon, where they can interact with microbial communities. Metagenomic analyses have shown carotenoid-associated changes in microbial composition and function, highlighting their potential role in shaping gut microbiota profiles [214,215,216,217]. Carotenoids not only influence the gut microbiota by altering its composition but also enhance intestinal barrier integrity, thereby reducing inflammation and improving metabolic outcomes [218]. Studies have demonstrated that carotenoids modulate gut microbiota composition in vivo, with specific compounds such as β-carotene, lycopene, and lutein altering the abundance of key bacterial taxa, including Short-Chain Fatty Acids (SCFA) producers and inflammation-associated genera [219]. Lutein was found to regulate nuclear factor erythroid 2-related factor (Nrf) 2, decrease TNF-α and IL-1β, and increase *Subdoligranulum* and *Faecalibacterium* in the gut [220]. Dietary intake of lutein and zeaxanthin is associated with increased microbiota diversity and may support improved mental health in adults [221]. In vitro studies using *Osmundea pinnatifida*, a marine red alga belonging to the *Rhodophyta* family, and isolated carotenoids such as β-carotene, lutein, and lycopene further demonstrate compound-specific modulation of microbial diversity and SCFA production [222]. Collectively, these effects on microbiota structure, function, and immune signaling may suggest therapeutic potential for carotenoids in regulating gut–brain axis activity relevant to neurodevelopmental disorders such as ASD.

Astaxanthin is a xanthophyll carotenoid red pigment derived mainly from microalgae, such as *Haematococcus pluvialis* and has been approved for human consumption [223,224,225,226]. It was approved by the Food and Drug Administration (FDA) in 1999 as a dietary supplement and is available in both natural and synthetic forms. Although astaxanthin is a precursor of vitamin A, it has strong antioxidant and anti-inflammatory effects [224,225]. Among carotenoids, it exhibits particularly potent antioxidant activity and has been shown to have neuroprotective effects across various brain disorders, including ASD. However, humans cannot synthesise astaxanthin naturally and must obtain it through diet [223,227].

Oxidative stress and neuroinflammation are key mechanisms in ASD [11,18,22,228]. Astaxanthin plays a key role in regulating immune pathways. It inhibits immunologic pathways, including JAK/STAT3, NF-kB, and Microtubule Affinity-Regulating Kinases, and activates PI3K/AKT and Nrf1 [225,229,230]. Its anti-inflammatory effects involve the suppression of key cytokines, including IL-6, Vascular Endothelial Growth Factor (VEGF), IL-1β, and TNF-α [225,231,232,233]. Astaxanthin has been shown to improve hippocampal structure and cognitive function in models of fetal alcohol spectrum disorder involving oxidative stress [234]. Since oxidative stress and neuroinflammation play central roles in ASD [235], targeting these pathways may be beneficial.

Avraham et al. found that astaxanthin improved social behavior in a BTBR T+ Itpr3tf/J (BTBR) mouse ASD model [236]. In a valproic acid (VPA) mouse model, astaxanthin improved autistic-like behaviors (anxiety), reduced cognitive deficits, and decreased repetitive behaviors in the offspring. It also lowered oxidative stress markers in this model [237]. In diabetic rats with cognitive impairment, astaxanthin has been shown to reduce oxidative stress, inhibit nitric oxide synthase, and improve cognitive function [238]. These results indicate that astaxanthin may help counteract the behavioral and molecular disruptions observed in ASD.

Given the high prevalence of gastrointestinal symptoms in ASD, modulation of the gut–brain axis represents a relevant therapeutic target, and the antioxidant and anti-inflammatory properties of astaxanthin may contribute to both central and peripheral regulation [239]. Studies have shown that astaxanthin can cross the BBB and reach areas critical for cognitive and behavioral functions, such as the hippocampus and cortex [240]. Astaxanthin is a lipid-soluble biomolecule that is sensitive to light, oxygen, and high temperatures; therefore, different formulations have been developed to deliver astaxanthin in vivo, such as nanoemulsions (lipid-based carriers, polymeric systems, and inclusion complexes) [241]. Astaxanthin and omega-3, when used together or separately, have shown potential in improving autistic symptoms. Significant improvements were observed in social interaction, grooming behaviors, and reductions in cytokine levels, including IFN-γ, in the hippocampus and prefrontal cortex [242]. Lycopene supplementation improved cognitive and behavioral performance in aged mice via microbiota modulation and SCFA elevation. Fecal microbiota transplantation confirmed the causal role of the lycopene-altered microbiota in these outcomes [243]. This combination highlights the effectiveness of multi-target approaches for addressing ASD symptoms.

#### 2.2.2. The Potential Effect of Carotenoids on Epilepsy

This family of compounds, including carotenoids, lycopene, fucoxanthin, astaxanthin, zeaxanthin, lutein, neoxanthin, and violaxanthin, is the primary carotenoid found in algae and exhibits strong antioxidant, anti-inflammatory, and neuroprotective effects [244,245,246,247]. Because of these effects, carotenoids may potentially contribute to beneficial effects in epilepsy.

In a pentylenetetrazole (PTZ)-induced epilepsy rat model, lycopene, a carotenoid pigment, reduced seizure severity and improved memory. It lowers the expression of iNOS and Neuronal Nitric Oxide Synthase (nNOS) and reduces nitric oxide levels in the cortex and hippocampus [248]. In a rat model of pilocarpine-induced epilepsy, lutein treatment showed antiepileptic activity that affected the hippocampus. It reduces neuronal damage, oxidative stress, and serum TNF-α, indicating a neuroprotective effect [249]. In a DSS-induced colitis model, β-carotene mitigated disease severity by restoring intestinal epithelial structure and increasing anti-inflammatory taxa, such as *Faecalibacterium*, while reducing pro-inflammatory bacteria. These effects align with their roles in maintaining gut immune homeostasis [250]. VPA, commonly prescribed for epilepsy, has been linked to an increased risk of ASD and cognitive impairment in children of mothers treated during pregnancy [251]. Additionally, the β-carotene enrichment of beneficial bacteria and their anti-inflammatory effects support a role in regulating the gut–brain axis [252]. A positive association between β-carotene intake and microbiota diversity has also been observed in children [253].

Fucoxanthin, a yellow-orange carotenoid derived from algae, has been approved by the FDA for human use [244]. Studies have demonstrated that algal fucoxanthin has potent antioxidant and anti-inflammatory properties and can modulate both apoptosis and autophagy. Its ability to influence gut microbiota further supports its role in the gut–brain axis and neuroimmune regulation in neurological disorders, including epilepsy [104,254,255,256]. Fucoxanthin from brown seaweed exerts antioxidant and neuroprotective effects on neuronal human neuroblastoma SH-SY5Y cells [254]. In an in vitro model using LPS-stimulated RAW 264.7 cells, fucoxanthin reduced the expression of pro-inflammatory markers, including IL-6, Arginase 1, and COX-2. It increased the expression of anti-inflammatory mediators, including IL-10 and Heme Oxygenase-1 (HO-1). It also downregulated the expression of the inflammation-associated microRNA miR-146b, indicating its role in modulating inflammatory signaling pathways [256]. Fucoxanthin exerts antioxidant and anti-inflammatory effects in neuronal and microglial models by modulating Akt/NF-κB, MAPK/AP-1, and Nrf2/HO-1 signaling pathways, while enhancing BDNF expression and cellular antioxidant defenses [104,254,255,256,257]. Taken together, these findings underscore the neuroprotective, anti-inflammatory, and microbiota-mediated effects of carotenoids, which may be harnessed to manage epilepsy and related neurological conditions.

Astaxanthin, a powerful antioxidant and anti-inflammatory compound, has shown promising neuroprotective effects in epilepsy. Its ability to reduce oxidative stress, inflammation, and neuronal damage suggests its potential as a novel therapeutic complement to existing treatments. It has been found that neuroinflammation is involved in epilepsy, and anti-cytokine methods show promising potential for treating epilepsy [258,259,260]. Targeting inflammation and key immune pathways can help reduce seizure severity and improve treatment outcomes.

Kandy et al. investigated the effect of astaxanthin on excitotoxicity, a mechanism associated with neuronal damage in epilepsy. Using primary cortical neuronal cultures, they found that astaxanthin protects against neuronal damage, reduces harmful ROS levels, and prevents mitochondrial membrane damage. It also affects calcium levels and glutamate receptors, both of which are critical for controlling neuronal activity and seizure onset [261]. Lu et al. have demonstrated the therapeutic potential of astaxanthin in epilepsy. They found that astaxanthin prevents neuronal apoptosis and oxidative stress in epilepsy induced by amygdala inflammation [262]. Oxidative stress is a significant factor in epileptic seizures and can interfere with the efficacy of antiepileptic drugs. Astaxanthin improves epileptogenesis independently; however, when combined with VPA, it enhances drug efficacy, protects the hippocampus, and reduces oxidative stress markers [263].

Status Epilepticus (SE) is a severe form of epilepsy that often does not respond to standard treatments. Astaxanthin has been proposed as a potential therapeutic agent because of its ability to target the P2X purinoceptor 7 receptor (P2X7R) in microglia. P2X7R, an ATP-gated ion channel expressed in activated microglia, plays a central role in inflammasome activation and IL-1β release. Targeting P2X7R reduces microglial overgrowth and inflammation in the hippocampus [241]. Astaxanthin also reduces the levels of inflammatory markers in the cortex and hippocampus when administered before the onset of SE. It has been found to improve cognitive dysfunction and decrease seizure frequency [264]. Administration of astaxanthin after SE improved cognitive dysfunction, reduced hippocampal damage, and decreased oxidative stress and inflammation by targeting the NF-κB pathway. It also reduces neuronal death via the PI3K/Akt pathway [265]. Maternal epileptic seizures, as well as antiepileptic drugs, affect the offspring’s brain development and cognitive function. When administered to pregnant mothers, astaxanthin has been shown to prevent hippocampal neuronal damage and reduce oxidative stress in offspring [266].

Collectively, algae-derived carotenoids target oxidative stress, neuroinflammation, and synaptic dysfunction while potentially enhancing antiepileptic drug efficacy, supporting their promise as multi-target modulators in ASD and epilepsy.

### 2.3. Polyunsaturated Fatty Acids (PUFAs)

Key lipid molecules, known as PUFAs, provide biological membranes with structural and functional characteristics through the presence of multiple double bonds in their hydrocarbon chains [267]. Omega-3 and omega-6 fatty acids are essential for human health because they are precursors of eicosanoids and other signaling molecules that regulate the cardiovascular, neurological, and immunological systems [268]. Although fish oil has long been the principal dietary source of omega-3 fatty acids, algae, specifically microalgae, are increasingly recognised as the primary sources of these fatty acids in the marine food chain [269]. Unlike fish-derived oils, algal-derived PUFAs offer a sustainable, reliable, and high-quality source of omega-3 fatty acids, providing environmental and nutritional benefits [270,271]. They also have lower contamination levels (e.g., heavy metals or polychlorinated biphenyls) and are acceptable to vegetarian and vegan consumers [272]. Recent research has revealed that algal omega-3 has substantial bioactivity, including anti-inflammatory, cardioprotective, neuroprotective, and anti-cancer properties. Algal omega-3 can reduce inflammation by inhibiting NF-κB activation and lowering pro-inflammatory cytokines [273]. Lipid extracts containing 34–42% total fatty acids as n-3 PUFA from three red seaweeds (*Porphyra dioica*, *Palmaria palmata* and *Chondrus crispus*) and one microalga (*Pavlova lutheri*) showed anti-inflammatory effects mediated via NFkB [274]. Several studies have shown that algae such as *Chlorella* and *Nannochloropsis oculata* are rich in fatty acids (including omega-3 and omega-6, eicosapentaenoic acid (EPA), and docosahexaenoic acid (DHA)) [275] and have been cultured in large-scale structures for the production and commercialization of PUFA to create functional foods or supplements with health benefits [276].

It has also been shown that omega-3 improves lipid profiles, cognitive function, and visual development [277]. Given the growing demand for sustainable omega-3 alternatives, algae-derived PUFAs offer a reliable option for clinical nutrition and functional foods. While direct evidence for algae-derived PUFA in ASD and epilepsy remains limited, the established neuroimmune and neuroprotective effects of PUFAs support their relevance to these conditions.

#### 2.3.1. The Potential Effect of PUFA on ASD

PUFAs, particularly omega-3 and omega-6, are essential for proper neurodevelopment and brain function. They play critical roles in maintaining the cell membrane structure, regulating neural signaling, controlling inflammation, and modulating gene expression [278]. Omega-3 includes alpha-linolenic acid (ALA), EPA, and DHA. The human body cannot synthesize these essential fatty acids, necessitating dietary intake. EPA from red algae has a strong anti-inflammatory effect by inhibiting NO [279]. Genetic polymorphisms affecting PUFA metabolism, particularly in Fatty Acid Desaturase 2 and Elongation of Very Long Chain Fatty Acids Protein 2, may lead to altered neuronal fatty acid profiles, which in turn affect membrane fluidity and signal transduction, and increase the risk of ASD [280,281,282]. Clinically, PUFA supplementation has been associated with improvements in core ASD symptoms, including social interaction, hyperactivity, and repetitive behaviors, likely through anti-inflammatory effects such as reduction in pro-inflammatory cytokines, activation of Peroxisome Proliferator-Activated Receptor Gamma (PPARγ), and enhancement of sleep quality [283,284,285,286,287]. Furthermore, supplementation with a combination of DHA and arachidonic acid (ARA; omega-6) resulted in mild improvements in social motivation and behavior, although the effects were weaker than those observed with risperidone. These benefits are likely due to enhanced neuronal signaling, neurotransmitter release, and reduced neuroinflammation. Additionally, PUFA supplementation increased insulin-like growth factor 1 levels and reduced ceruloplasmin levels, suggesting attenuation of neuroimmune activation [288].

Omega-3 fatty acids have been investigated in ASD with mixed clinical outcomes [289,290,291,292]. Jiang et al. suggested that omega-3, when combined with vitamin D, has a positive effect on ASD, although omega-3 alone does not provide the same benefit [290,291]. In contrast, Woods et al. found that when omega-3 was administered alone, there was a slight improvement in social communication and repetitive behaviors, whereas combining it with vitamins did not provide additional benefits [293]. Johnson et al. reported no significant benefit, with limitations including small sample size and lack of placebo controls [294]. Omega-3 fatty acids play a role in mental health and may also affect ASD through their involvement in the regulation of neuroinflammatory responses and different brain pathways. However, their specific effects on ASD have not yet been fully elucidated [292]. Doherty et al. found insufficient evidence to support the effectiveness of omega-3 fatty acids in treating ASD [295]. However, studies have suggested that omega-3 may be associated with certain symptoms. For example, a meta-analysis found that omega-3 supplements helped reduce speech problems and hyperactivity in children under 8 years of age. However, doses of 1000 mg or less were linked to increased repetitive behavior, suggesting that the effects of omega-3 can depend on the dosage and specific symptoms [296].

Studies have explored the potential protective effects of omega-3 when combined with other nutrients or administered under specific conditions. It has been found that omega-3-6-9 supplementation improves anxiety, depression, and autistic symptoms in premature babies [290,291,297,298]. Premature babies treated with DHA and ARA had a lower risk of developing ASD [299]. Omega-3 and vitamin B12 have also shown protective effects in mouse models of ASD [300]. In humans, regular fish consumption during pregnancy was associated with a 20% lower risk of ASD in children, although omega-3 fish oil supplementation during pregnancy did not show significant benefits [301]. Omega-3 supplementation during pregnancy has been shown to support brain development in children born to mothers with risk factors for neurodevelopmental disorders [302].

Dose and symptom targeting may influence omega-3 outcomes in ASD. Maternal DHA levels have not shown a consistent association with ASD risk [303]. Meta-analytic evidence suggests improvements in social communication and hyperactivity, with possible worsening of repetitive behaviors at lower doses [296]. Almohmadi suggested that omega-3 supplementation improves social communication and reduces hyperactivity in children with ASD. Mechanistically, omega-3 may influence ASD-relevant pathways through anti-inflammatory effects and modulation of gut–brain axis signaling [304].

Omega-3 fatty acids, particularly DHA and EPA, are frequently found at reduced levels in individuals with ASD, likely because of poor dietary intake and metabolic disturbances [283,305]. DHA and ARA together comprise up to 25% of the fatty acids in the gray matter of the brain and contribute to membrane stability, neuronal function, and modulation of neuroinflammation [288]. In a mouse model of prenatal exposure to VPA, supplementation with omega-3, either plant-derived ALA or marine-derived DHA and EPA, prevented the development of ASD-like behavioral symptoms, including impaired social interactions, stereotypic behavior, and loss of Purkinje cells in the cerebellum. A reduction in cerebellar TNF-α levels was observed, indicating a clear anti-inflammatory effect. A diet enriched with DHA and EPA from marine sources was more effective in altering the brain fatty acid composition and reducing inflammatory markers [306]. PUFAs serve as critical structural cofactors for the activity of the tandem pore domain halothane-inhibited K+ channel 1 (THIK1) potassium channel, which regulates microglial synaptic pruning and neuro-inflammation. Marine-derived DHA and EPA are among the most potent modulators of THIK1 activity, binding to a specific PUFA-recognition site and altering channel gating.

These findings highlight the therapeutic potential of PUFAs, especially those derived from marine sources, for treating neurological conditions, including ASD [307].

DHA and EPA have also been associated with modulation of microbiota composition and gut–brain axis signaling, including effects on neuroactive metabolites and inflammatory process [308,309,310,311,312]. Under malnutrition, PUFA supplementation restored cortical DHA levels and was linked to microbiota-dependent microglial changes [313]. early-life stress and metabolic states may amplify PUFA–microbiota interactions relevant to neurodevelopment and behavioral improvements observed in ASD [314,315].

#### 2.3.2. The Potential Effect of PUFA on Epilepsy

Marine and plant-derived PUFAs have been found to modulate Potassium Voltage-Gated Channel Subfamily Q, which regulates neuronal excitability [316]. PUFAs enhance channel activity by shifting the activation to more negative potential and increasing ion flow, thereby reducing neuronal hyperexcitability. This suggests therapeutic potential in epilepsy and possibly ASD, although behavioral studies are still needed [317,318,319]. Furthermore, PUFAs, particularly omega-3 fatty acids such as DHA and EPA, show potential antiepileptic effects. Clinical studies have reported reduced seizure frequency and lower levels of pro-inflammatory cytokines in patients with drug-resistant epilepsy following PUFA supplementation. Preclinical models support these findings by demonstrating improved mitochondrial function, decreased neuroinflammation, and stabilisation of neuronal excitability. The proposed mechanisms involve the activation of PPARγ and the suppression of inflammatory pathways, suggesting that PUFAs may serve as beneficial adjunctive therapies in epilepsy, particularly in cases with neurodevelopmental comorbidities [283,284,320]. A deficiency of omega-3 fatty acids early in life increases the risk of seizures later in life. Fatty acids, such as DHA and EPA, are important for healthy brain development and are mainly found in fatty fish and algal oil. In mice, omega-3 deficiency before and after birth led to increased susceptibility to seizures in adulthood. When adult mice received omega-3 supplements for 17 days, seizure sensitivity was reduced. The most effective supplement is rich in DHA bound to phospholipids, which is more easily absorbed in the brain [321].

Omega-3s have been studied for their antiepileptic potential, including reducing seizures and improving overall brain health in individuals with epilepsy. It has been demonstrated that omega-3 fatty acids may help reduce seizure frequency and severity in epilepsy by decreasing inflammation and protecting brain cells [322]. A meta-analysis found that omega-3 supplementation reduced seizure frequency in people with epilepsy. It had a stronger effect in adults when the daily dose was 1500 mg or less and the treatment lasted over 16 weeks. However, children showed less benefits from omega-3 supplementation [323]. Similarly, omega-3 supplementation, given as EPA and DHA linked to a phospholipid vector (PS-Omega3), did not show significant benefits in reducing seizures or improving epilepsy symptoms in children with both ADHD and epilepsy [324]. However, animal studies have yielded mixed results. Long-term omega-3 intake did not significantly reduce epileptic discharges or seizure activity in rats with stroke-induced epilepsy [325]. However, high doses of DHA combined with antiepileptic drugs reduce seizure frequency in some dogs, indicating potential benefits [326]. In addition, omega-3 fatty acids from fish have a protective effect against epilepsy in dogs [327]. A ketogenic diet enriched with polyunsaturated fatty acids, including omega-3, showed promising results in reducing seizures in children with refractory epilepsy compared with the traditional ketogenic diet [328]. The clinical and immunological findings further highlight these complexities. In one trial of omega-3 supplementation for 16 weeks, patients showed a significant increase in IFN-γ levels, while transforming growth factor-beta (TGF-β) levels remained the same. However, the treatment did not directly affect seizure frequency, suggesting that omega-3 may influence immune function in epilepsy without immediately affecting seizures [329]. Broader reviews emphasize promise and uncertainty. A review by Alhattab et al. highlighted the potential benefits of omega-3s in reducing seizures but emphasized the need for more research to confirm these effects on epilepsy [330]. Liang et al. found that a genetic increase in blood omega-3 levels was linked to a higher risk of developing epilepsy, suggesting a possible causal connection and providing new insights into the mechanisms underlying epilepsy [331]. Overall, evidence remains limited and inconsistent, particularly in pediatric epilepsy [332,333].

ALA enhances the function of GABA receptors, contributing to the regulation of overactive brain activity. A study that did not focus directly on epilepsy suggested that ALA may have therapeutic potential in conditions characterized by excessive neural activity, such as epilepsy [334]. A study on the ketogenic diet, which includes omega-3, found that it reduced seizure severity in a rat model of temporal lobe epilepsy and improved behavior in the open-field test [335]. Sadat-Hossieny et al. examined whether omega-3, combined with folic acid and other nutrients, could improve brain development in the children of mothers with epilepsy. However, the study found no significant link between omega-3 intake and children’s cognitive development [336].

PUFAs may also influence epilepsy-relevant mechanisms through gut–brain axis pathways involving barrier integrity and immune signaling. In diet-induced obesity models, omega-3 enhanced gut barrier markers (e.g., ZO-1, occludin), increased colonic BDNF, and reduced intestinal inflammation via TLR4 suppression [337]. Omega-3 improves learning and memory by suppressing inflammatory processes along the gut–brain axis. Omega-3 intake also attenuated gut–brain inflammatory signaling through reduced activation of the TLR–MyD88–NF-κB pathway and decreased pro-inflammatory cytokine expression [338]. In models of diabetes and Parkinson’s disease-associated cognitive impairment, both of which share neuroinflammatory and gut dysbiosis components with epilepsy, omega-3 improves cognitive outcomes, gut barrier integrity, and microbiota composition, including increased *Lactobacillus* and improved *Firmicutes/Bacteroidetes* balance [290,291,339,340]. Collectively, these findings suggest that omega-3 fatty acids may have antiepileptic benefits by stabilizing gut–brain inflammatory and neuroimmune signaling pathways.

PUFAs have the potential to address the specific symptoms and risk factors associated with ASD and epilepsy. Their anti-inflammatory effects, support for brain development, and gut–brain axis effects highlight their therapeutic importance. Further research should define optimal dosing, formulation, and target phenotypes, including evaluation of algae-derived PUFA as sustainable sources relevant to ASD and epilepsy.

### 2.4. Polyphenols

Polyphenols are a diverse group of plant-derived compounds with powerful antioxidants, anti-inflammatory, and neuroprotective properties found in plants, fruits, and marine algae. These traits make them beneficial candidates for regulating neurodevelopmental disorders, such as ASD, which is increasingly considered to be associated with chronic neuroinflammation, oxidative stress, reduced mitochondrial function, and abnormalities in the gut–brain axis [341,342]. Notably, many polyphenols can cross the BBB and enhance mitochondrial function—key attributes for neurotherapeutic agents [343,344,345,346,347,348].

Flavonoids, a variety of polyphenolic secondary metabolites, have long been investigated in terrestrial plants because of their important roles in plant defense, pigmentation, and signaling. More recently, these compounds have been discovered in algal species, particularly in green and red algae [349]. Flavonoids in algae play a crucial role in antioxidant defense mechanisms by neutralizing ROS produced during photosynthesis and in response to environmental factors, such as UV radiation, salinity variations, and nutrient scarcity [349]. In addition to their antioxidant properties, algal flavonoids also exhibit significant anti-inflammatory and immunomodulatory effects. Mechanistically, these compounds have been shown to inhibit key inflammatory mediators, including COX-2 and iNOS, and pro-inflammatory cytokines [350]. Such effects are mediated by the modulation of signaling pathways, including NF-κB and MAPKs, suggesting potential applications in the prevention and management of chronic inflammation-related diseases [351]. Emerging research has also linked algal flavonoids to anti-cancer, antimicrobial, and neuroprotective activities [352]. Overall, algae-derived flavonoids show promise for functional foods, nutritional supplements, cosmeceuticals, and medicinal formulations, although additional in vivo validation and toxicological studies are still required [353].

#### 2.4.1. The Potential Effect of Polyphenols on ASD

Preclinical studies indicate that polyphenols modulate multiple ASD-related pathways, including suppression of pro-inflammatory signaling, attenuation of oxidative stress, and activation of antioxidant responses (e.g., Nrf2-associated pathways) [348,354,355,356,357,358,359]. Polyphenols additionally support mitochondrial function, which is typically decreased in ASD, by improving cellular energy metabolism and protecting against oxidative stress in both human and animal trials [360,361].

Resveratrol, one of the most well-studied polyphenols, has been shown to improve social behavior, memory, and anxiety-related symptoms in mouse models of ASD. Research suggests that it has anti-inflammatory and neurotransmitter-modulating effects [362,363,364,365]. Other polyphenols, such as those found in blackberries and grape seeds, also show neurobehavioral advantages, most likely due to their antioxidant and signaling pathway modulation capabilities [344,345,366,367,368,369]. Marine-derived polyphenols, particularly phlorotannins from brown algae, boost these advantages by enhancing cognitive performance and reducing sleep disorders, both of which are typical conditions in children with ASD [348,354,355,356].

Polyphenols may also influence ASD-relevant outcomes through microbiota–gut–brain axis modulation. Studies report increased beneficial taxa (e.g., *Bifidobacterium* and *Lactobacillus*) and reduced potentially pathogenic taxa following polyphenol exposure [370,371,372,373]. These microbial changes have been linked to reduced gut permeability, lower intestinal inflammation, and enhanced barrier function, all of which affect brain function and behavior [374,375]. Green tea and algae such as *Delisea pulchra* contain epigallocatechin gallate (EGCG), which has been demonstrated to improve behavioral symptoms and gut microbiota in children with ASD [376,377,378,379].

Luteolin, quercetin, and apigenin have been shown in ASD models to improve social interaction, minimize repetitive activities, and elevate antioxidant defenses [362,380,381,382,383,384,385,386,387,388]. Other flavonoids, such as baicalin and hesperetin, have been shown to increase mitochondrial activity and neuronal development, resulting in improved cognitive outcomes [389,390].

#### 2.4.2. The Potential Effect of Polyphenols on Epilepsy

In addition to their therapeutic potential in ASD, polyphenols, especially algae-derived polyphenols, have shown potential for epilepsy management. In preclinical epilepsy models, the polyphenol resveratrol has been shown to reduce seizure frequency and intensity by modulating neurotransmission (including glutamatergic and GABAergic signaling), reducing oxidative stress, and suppressing inflammatory pathways [346,362,391,392,393]. Resveratrol also protects the BBB and improves neuronal survival in kainic acid-induced seizures [343,394,395]. Additionally, a study on turmeric and resveratrol in a lithium/pilocarpine-induced status epilepticus model demonstrated prolonged latency to the first generalized seizure and reduced seizure frequency without hepatotoxicity or nephrotoxicity [396].

Among stilbenes, resveratrol, pterostilbene, piceatannol, and polydatin have demonstrated potential therapeutic effects in epilepsy. However, resveratrol has high oral absorption (~75%) but exhibits low bioavailability (<1%) owing to its rapid metabolism into glucuronides and sulfates, highlighting a common barrier for plant-derived compounds [362,397]. Several formulation strategies have been explored to enhance the bioavailability and efficacy of resveratrol in the treatment of neurological disorders. Nano-encapsulation with glutathione-coated nanoparticles improved brain delivery, leading to reduced hippocampal inflammation and cognitive impairment in a PTZ-induced epilepsy mouse model. Notably, nano-resveratrol requires significantly lower doses to achieve seizure control than conventional resveratrol [398,399]. Similarly, glutathione-coated collagen nanoparticles enhance targeted brain delivery, effectively suppressing acute seizures by inhibiting the High Mobility Group Box 1 and TLR-4 signaling pathways [392].

However, some studies have reported limited anticonvulsant efficacy of resveratrol. In a PTZ-induced seizure model in zebrafish, neither resveratrol nor piceatannol reduced the seizure incidence, suggesting model-dependent variability [400]. In contrast, micronized resveratrol, formulated to enhance bioavailability, effectively delayed seizure progression and prevented tonic–clonic phases in a PTZ-induced seizure model in both zebrafish larvae [401] and adult zebrafish. In contrast to antiepileptic diazepam, micronized resveratrol did not cause behavioral side effects. It also regulates *c-fos* expression, highlighting its potential as a safe treatment [402].

The effects of polyphenols on epilepsy extend beyond those of resveratrol. In addition to resveratrol, various polyphenols have been shown to have antiepileptic effects. Curcumin, for example, has demonstrated a broad neuroprotective effect by minimizing mitochondrial oxidative stress, lipid peroxidation, and seizure-induced brain damage. It functions via modulating GABAergic signaling, inhibiting MAPK and mTOR pathways, and regulating autophagy and neuroinflammatory cascades [403,404,405,406]. Quercetin also demonstrates anticonvulsant activity through NF-κB inhibition, antioxidant effects, and preservation of cognitive functions. It modulates GABA and N-methyl-D-aspartate (NMDA) receptors, reduces neuroinflammation, and regulates the Sirtuin 1/Nrf2 signaling axis in epilepsy models [342,407,408,409].

Flavonoids derived from marine and plant sources have additional therapeutic benefits. Baicalin lowers seizure severity and oxidative stress by regulating B-cell lymphoma 2 and miR-497 [410], whereas ferulic and caffeic acids alter GABAergic transmission and inflammatory cytokines [347,411,412,413]. Green tea and some algae contain EGCG, which affects TLR4/NF-κB signaling, dopamine, and GABA-A receptor pathways, leading to anticonvulsant activity [395,414,415,416,417]. Additionally, luteolin, taxifolin, hispidulin, and apigenin demonstrate neuroprotective properties by modulating inflammatory pathways, supporting synaptic stability, and enhancing GABAergic signaling [418,419,420,421,422].

Emerging evidence supports gut–brain interactions in epilepsy through effects on inflammation, barrier integrity, and neurotransmission. In a chronic unpredictable mild stress rat model, quercetin improved affective behavior and cognition alongside microbiota shifts and coordinated changes in brain metabolic pathways [423]. Apigenin, a flavone found in red algae [424], was tested in a water avoidance stress rat model. It reduced abdominal pain sensitivity and gut motility by restoring gut microbial balance, strengthening the intestinal barrier, and reducing mast cell and microglial activation. Its effects are mediated through inhibition of the TLR4/MyD88/NF-κB signaling pathway, a route closely associated with neuroinflammation and relevant to epileptogenesis [425]. Nevertheless, these findings highlight the multi-target antiepileptic potential of polyphenols, spanning antioxidant defense, neuroinflammation reduction, mitochondrial support, synaptic modulation, and gut–brain axis regulation. While clinical trials remain limited, preclinical evidence supports polyphenols as candidate adjunctive therapies, with future work needed on formulation, absorption, and long-term safety.

### 2.5. Vitamins

Algae are increasingly being recognised as rich natural sources of vitamins, contributing substantially to human nutrition and providing bioavailable forms of both water-soluble and fat-soluble vitamins. Algal species, notably microalgae such as *Chlorella*, *Spirulina*, *Dunaliella*, and *Nannochloropsis*, may synthesize a wide range of vitamins, including A, B-complex, C, D, E, and K, owing to their rich taxonomy and flexible metabolic pathways [426]. B-complex vitamins such as B1, B2, B3, B6, and B12 are water-soluble and can be found in high concentrations in *Spirulina* and *Chlorella* [427]. Because of their high levels of nutrients and bioactive compounds, algal vitamins have been widely studied as functional foods, nutraceuticals, and for therapeutic applications that focus on preventing or managing micronutrient deficiencies [428]. These attributes position algae-derived vitamins as potential supportive components in strategies targeting ASD and epilepsy.

#### 2.5.1. The Potential Effect of Vitamins on ASD

Micronutrients, including vitamins, are increasingly recognized for their roles in neurodevelopment, mitochondrial function, oxidative balance, and neurotransmitter synthesis. Vitamins B12, B6, A, D, E, K, and C have been examined in ASD in relation to symptom severity, metabolic alterations, and immune regulation. Clinical findings are variable. Elevated B12 levels have been reported in some children with ASD, particularly those with epilepsy [429], whereas other studies link reduced B12 levels to greater symptom severity and impaired social interactions [430,431]. In ASD BTBR mice, fecal microbiota transplantation and vitamin B6 supplementation improved social behavior and neurotransmitter synthesis while enhancing mitochondrial and microbial regulation [432,433]. Vitamins A and D have also been implicated in ASD. Peripheral vitamin A levels are frequently lower in children with ASD and may correlate with neurodevelopmental mechanisms [431,434]. In rodent models, vitamin A supplementation improved core ASD-like behaviors and restored circulating vitamin A levels [435]. Low vitamin D levels have been associated with increased ASD severity and altered brain activity patterns [436], and supplementation combined with exercise reduced inflammation (lower IL-6, higher IL-10) and improved social interaction in boys aged 6–14 [437]. However, genome-wide association studies and Mendelian randomization analysis found that vitamin D does not have a direct correlation with ASD but rather is a downstream consequence of altered metabolism associated with the condition [303,438]. Findings for vitamin E and C are inconsistent. Lower vitamin E levels have been associated with greater ASD severity in some studies [439], whereas others report elevated levels [440]. Vitamin C findings similarly vary, with reports of both elevated levels and deficiencies related to restrictive diets [441,442]. In a valproic acid-induced ASD rat model, vitamin C improved social behavior, reduced repetitive behavior, and enhanced cortical antioxidant enzyme activity [443,444]. Vitamin K, produced by marine algae including *Ulva lactuca*, has been associated with neuronal survival, oxidative stress regulation, and inflammatory modulation [445,446,447,448]. Reduced blood vitamin K levels correlate with social and language impairments in children with ASD [449]. Furthermore, modification of the vitamin K pathways has been discovered in postmortem brain tissue from individuals with ASD, confirming its neurodevelopmental significance [450].

Large-scale and clinical studies yield mixed results. In 1235 children with ASD, higher levels of vitamins A, E, B12, and D were associated with lower ASD severity scores, whereas other cohorts found no significant differences in B12, folate, or vitamin E levels between ASD and controls [451]. In a randomized, double-blind, placebo-controlled study in children with ASD, intravenous glutathione alone or combined with vitamin C and N-acetylcysteine did not significantly improve behavior or oxidative stress markers compared with placebo [452]. In a large observational study of 2614 mother–child pairs, higher maternal dietary intake of nutrients, vitamin D, and vitamin B12 was found to have the highest correlation with fewer ASD traits in children, based on the Social Responsiveness Scale scores. However, no significant associations were found between these nutrients and clinical diagnosis of ASD [453]. In a study of 226 young children with ASD, lower blood levels of vitamin D 25(OH)D were linked to altered brain activity in specific regions, including the postcentral gyrus (sensory processing), precuneus (social interaction), middle cingulate gyrus, superior frontal gyrus (cognition and empathy), middle temporal gyrus, and insula (language and emotional processing) [436].

#### 2.5.2. The Potential Effect of Vitamins on Epilepsy

In addition to their critical functions in neurological development and metabolic regulation, various vitamins have been linked to the development and management of epilepsy. Specific vitamin shortages or excesses may affect seizure susceptibility, neuroinflammation, and treatment outcomes either directly or through interactions with AEDs and gut function. For instance, Vitamin B6, present in microalgae such as *Tetraselmis*, is essential for GABA synthesis, and deficiency—particularly of pyridoxal 5′-phosphate—has been directly linked to seizure risk. In rare metabolic disorders, B6 supplementation restores seizure control in drug-resistant epilepsy [454]. Vitamin D, identified in species such as *Tetraselmis suecica* and *Skelatonema costatum*, exerts immunomodulatory and neuroprotective effects and has been associated with reduced neural damage in experimental epilepsy models [183,455]. Clinical studies report frequent vitamin D deficiency in patients receiving enzyme-inducing AEDs, and supplementation has been linked to improved seizure control, although causal relationships remain debated [456,457,458,459,460]. Vitamin E, a lipid-soluble antioxidant, protects neuronal membranes from oxidative injury and reduces neurodegeneration in experimental mouse models with poor lipid absorption caused by gut dysbiosis, showing its potential to treat seizure-related diseases [461], and joint vitamins E and C reduced seizure frequency in pediatric populations [462]. Vitamin K is another promising compound generally known for its role in coagulation. In a pyridoxine-dependent epilepsy model, vitamin K improved dendritic spine formation and synaptic signaling in the hippocampus, suggesting that it may help preserve neural integrity in seizure disorders [463]. Vitamin C, which is frequently linked to the immune system, is also essential for epilepsy because of its antioxidant properties and contribution to catecholamine production. In preclinical models of valproate-induced neurotoxicity, vitamin C administration enhanced spatial learning, reduced anxiety-like behavior, and lowered oxidative markers such as malondialdehyde [464]. The antioxidant, anti-inflammatory, and synaptic support activities of vitamins make them promising candidates for supplementary epilepsy therapy techniques. Future research should concentrate on algal-based vitamins for their bioavailability, pharmacokinetics, and synergistic potential of marine vitamins, particularly when administered via whole algae extracts or functional meals.

Vitamins also influence neurological function through modulation of the gut–brain axis. Vitamin K2 (menaquinone-7), found in marine sources, restored microbiota composition and improved cognition in antibiotic-induced dysbiosis models while reducing oxidative stress and preserving hippocampal structure [465]. Similarly, in a clinical study involving healthy young adults with low vitamin C levels, four weeks of vitamin C supplementation improved the gut microbiota by increasing the abundance of beneficial bacteria, such as *Bacillaceae* and *Anaerotruncus*, while reducing harmful *Desulfovibrio* bacteria. These microbial shifts were associated with reductions in inflammatory markers, along with better cognitive performance and higher BDNF levels [466]. Vitamin A, well known for its role in visual and immune functions, also exerts protective effects on the gut–brain axis, particularly under metabolic stress. In a C57BL/6J mouse model exposed to a high-fat, high-sugar diet early in life, vitamin A supplementation prevented memory impairment, abnormal neuronal activation in the hippocampus, and gut microbiota changes, thereby maintaining gut microbial diversity and protecting cognitive functions [467], whereas deficiency exacerbated cognitive decline and microbiota disruption in transgenic mouse model of Alzheimer’s disease [468]. In addition to specific vitamins, co-supplementation with probiotics and vitamin D3 for 12 weeks in patients with Parkinson’s disease significantly reduced inflammatory cytokines and oxidative stress. Clinically, these alterations improve anxiety, gastrointestinal function, and overall disease severity [469]. Additionally, 25-hydroxyvitamin D3 has been shown to modulate neurotransmitter levels and gut-derived hormone signaling alongside microbiota changes, suggesting that vitamin D is a key regulator of the gut–brain axis [470]. Together, these findings underscore the therapeutic potential of vitamins in modulating the gut–brain axis. Marine-derived vitamins represent a multi-targeted strategy for managing brain health.

Collectively, these algae-derived compounds converge on a limited number of shared targets, including NF-κB signaling, oxidative stress regulation (Nrf2 pathway), mitochondrial function, excitatory–inhibitory balance, and gut–brain axis modulation (Table 3 and Table 4). Despite differences in chemical structure, their overlapping mechanisms suggest that combination strategies or whole-algae preparations may provide additive or synergistic effects. However, the majority of available data remain preclinical, and rigorously designed clinical trials are required to determine optimal dosing, safety, and translational applicability in ASD–epilepsy comorbidity.

## 3. Discussion

The mechanistic convergence between ASD and epilepsy characterized by neuroinflammation, oxidative stress, and gut–brain axis disruption provides a biologically coherent framework for exploring multi-target interventions. Algae-derived bioactive compounds demonstrate modulatory effects across these interconnected pathways. However, several translational challenges must be addressed.

### 3.1. Preclinical–Clinical Discrepancy

Most evidence supporting the role of algae-derived compounds in ASD and epilepsy comes from preclinical models. These models include valproic acid-induced ASD paradigms, chemically induced seizure models, and inflammatory or metabolic systems. Consistent findings from these studies indicate reductions in oxidative stress markers, suppression of pro-inflammatory signaling, and partial restoration of synaptic plasticity.

Nevertheless, the hypothesis of ASD–epilepsy comorbidity requires caution. ASD is a diverse neurodevelopmental condition characterized by varying cognitive, behavioral, and immunological profiles, while epilepsy encompasses diverse etiologies ranging from genetic channelopathies to acquired structural damage. Model systems rarely capture this complexity, and seizure suppression in acute paradigms does not necessarily predict long-term clinical efficacy or co-resistance outcomes.

Clinical trials evaluating omega-3 fatty acids or selected polyphenols report modest and symptom-specific benefits, yet findings remain inconsistent. Variability in dosing, age, phenotype stratification, and trial duration likely contributes to heterogeneous results. Well-designed randomized controlled trials targeting clearly defined ASD–epilepsy subpopulations are urgently needed.

### 3.2. Bioavailability and Dose Considerations

Natural compound translation faces a significant limitation in pharmacokinetics. For instance, polyphenols like resveratrol have low bioavailability because they are rapidly metabolized. Carotenoids, being lipophilic, require absorption, while polysaccharides’ activity may depend more on microbiota-mediated fermentation than systemic circulation. Even for PUFAs, dose-dependent effects and formulation variations can influence the outcomes.

Emerging delivery strategies, including nano-encapsulation and phospholipid-bound systems, have improved brain penetration in experimental models. However, there is a need for more comprehensive data on pediatric safety and long-term tolerability. Establishing optimal dosing ranges and therapeutic windows, especially in children, and considering the potential interactions with AEDs, remains a crucial priority.

### 3.3. Phenotypic Heterogeneity and Precision Approaches

The ASD–epilepsy comorbidity is biologically diverse. Genetic mutations affecting synaptic scaffolding proteins or ion channels can lead to distinct inflammatory and metabolic profiles. Consequently, uniform nutritional interventions are unlikely to produce consistent effects across all patients.

Future strategies could benefit from a precision-based framework that incorporates genetic background, inflammatory biomarkers, microbiota composition, and metabolic profiling. Sub-group supplementation approaches guided by biological markers could improve response predictability and minimize unnecessary exposure.

### 3.4. Gut–Brain Axis as a Convergent Target

Algae-derived compounds have several compelling translational applications. They can modulate the composition of gut microbiota, maintain intestinal barrier integrity, and influence microbial metabolite production. Both ASD and epilepsy have been linked to dysbiosis, increased gut permeability, and systemic immune activation.

Algal polysaccharides, carotenoids, and PUFAs exhibit prebiotic and anti-inflammatory effects in experimental models, often accompanied by increased short-chain fatty acid production and reduced peripheral cytokine levels. However, it remains to be determined if such microbiota-mediated modulation translates into consistent neurological improvement in humans. Longitudinal clinical studies incorporating microbiome, metabolomic, and neurobehavioral endpoints are necessary to establish this.

### 3.5. Safety and Regulatory Considerations

Marine-derived compounds are generally considered safe and widely used in nutraceuticals, but long-term safety data in neurodevelopmental populations is limited. Careful evaluation of chronic modulation of inflammatory and oxidative pathways is crucial, especially for pediatric patients receiving AED polytherapy.

Standardizing extraction methods, ensuring compound purity, and maintaining batch consistency are crucial for clinical translation. Furthermore, distinguishing between dietary supplementation and therapeutic use will significantly impact regulatory pathways.

### 3.6. Future Directions

Future research should prioritize the following areas: (1) Mechanistically stratified clinical trials in ASD–epilepsy cohorts, (2) Pharmacokinetic optimization and formulation studies, (3) Biomarker-guided intervention frameworks, (4) Evaluation of synergistic combinations (e.g., carotenoids with PUFAs), and (5) Integration of microbiome and immune profiling into clinical designs.

In summary, while definitive clinical validation is still limited, the convergence of biological pathways targeted by algae-derived compounds suggests their potential as adjunctive modulators of ASD–epilepsy comorbidity. However, rigorous translational research is now necessary to determine whether marine-inspired multi-target strategies can meaningfully impact neurodevelopmental outcomes.

## 4. Materials and Methods

This review was conducted as a narrative synthesis of published literature.

### 4.1. Search Strategy and Data Sources

A comprehensive electronic literature search was systematically conducted across three primary databases: PubMed, Web of Science, and Scopus. The search covered all indexed literature from database inception between the years 2020 and 2025, using the following search terms: (Autism OR ASD) AND (epilepsy OR seizures), (Autism OR ASD) AND (algae OR marine compounds OR inflammation OR oxidative stress OR gut-brain axis OR brain OR microbiome), (epilepsy OR seizures) AND (algae OR marine compounds OR inflammation OR oxidative stress OR gut-brain axis OR brain OR microbiome), (Autism OR ASD OR epilepsy OR seizures) AND (carotenoids OR astaxanthin OR polyunsaturated fatty acids (PUFA) OR omega-3/6 OR polysaccharides OR β-glucan OR fucoidan OR polyphenols OR flavonoids OR vitamin B/K/D/C/A/E). Given these overlapping pathologies, marine algae have emerged as compelling therapeutic sources. Their bioactive compounds simultaneously target inflammation, oxidative stress, and neurotransmitter balance, offering unique opportunities for dual interventions in ASD and epilepsy. Reference lists of key publications were also screened.

### 4.2. Inclusion and Exclusion Criteria

To be considered for evaluation, studies had to meet the following predefined criteria:

Inclusion Criteria: (1) Peer-reviewed primary research manuscripts including both in vitro/in vivo preclinical models and clinical trials; (2) Studies focusing directly on functional components derived from marine algae (e.g., fucoidan, carotenoids, polyphenols); (3) Studies addressing clear mechanistic pathways relevant to ASD–epilepsy comorbidity, such as inflammatory, oxidative stress, synaptic, or microbiota-related pathways.

Exclusion Criteria: (1) Document types such as conference abstracts, editorials, patents, or book chapters; (2) Non-English-language manuscripts; (3) Studies analyzing terrestrial plant extracts without marine relevance.

### 4.3. Study Selection and Data Extraction

The study selection process was executed in a multi-stage screening workflow. Initially, duplicate records across databases were identified and removed. Next, the titles and abstracts of the remaining manuscripts were screened based on the inclusion and exclusion criteria. In the final stage, full-text articles were comprehensively evaluated for final inclusion. Additionally, the reference lists of all relevant original studies, systematic reviews, and meta-analyses were manually screened to identify any missed landmark publications.

A total of 480 manuscripts met all criteria and were included in the final narrative synthesis.

## Figures and Tables

**Table 1:** Shared mechanisms in ASD and epilepsy.

Mechanism	ASD	Epilepsy
Inflammation	Microglial activation; elevated cytokines (IL-6, IL-1β, IL-8, TNF-α, IFN-γ, IL-4, IL-21); JAK/STAT and mTOR signaling; inflammation in cortex, amygdala, and hippocampus [15,16,18,20,53]	Microglial activation; mTOR, COX-2, and JAK/STAT pathway activation; immune-mediated BBB disruption; elevated IL-1β, IL-6, IL-8; neuroinflammation [53,58,68,70,95]
Oxidative Stress	Increased ROS production and lipid peroxidation; oxidative imbalance [11,22,57]	Oxidative imbalance; ROS production linked to mTOR activation; reduced antioxidant defense [53,95]
Gut–Brain Axis	Altered microbiota composition; increased gut permeability; altered SCFA production; neurotransmitter dysregulation [33,35,39,40]	Gut dysbiosis; altered neurotransmission; increased BBB permeability; SCFA-mediated modulation; probiotics associated with reduced seizure frequency [85,86,92,93,96,98,99]

Abbreviations: ASD, autism spectrum disorder; BBB, blood–brain barrier; COX-2, cyclooxygenase-2; IFN-γ, interferon-gamma; IL, interleukin; JAK/STAT, Janus kinase/signal transducer and activator of transcription; mTOR, mechanistic target of rapamycin; ROS, reactive oxygen species; SCFA, short-chain fatty acids; TNF-α, tumor necrosis factor-alpha.

**Table 2:** Representative chemical structures of major algae-derived compound classes discussed in this review. Schematic diagrams and chemical structures were created with ChemDraw and Canva.

Polysaccharides	Polyphenols
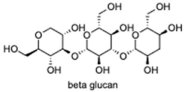	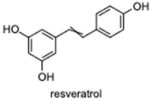
**Cartenoids**	**PUFA**
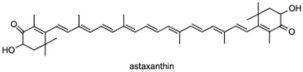	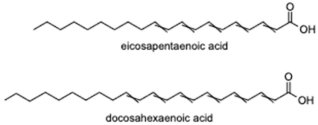
**Vitamins**	
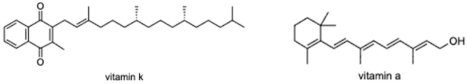	

**Table 3:** Summary of algae-derived compound classes and their reported behavioral and clinical effects in ASD and epilepsy (↑ represents increased outcome, ↓ represents decreased outcome).

Compound Class	Active Compounds	Behavior	Brain Changes	Epilepsy	Refs.
Polysaccharides	Fucoidan β glucan	↑ Social behavior ↑ Memory ↓ Anxiety	↑ Cognitive function ↑ BDNF ↑ PSD95	↓ Seizure frequency ↓ Seizure severity ↓ Seizure onset	[141,148,149,150,155,156,158,160,161,162,173,181,471,472,473]
Carotenoids	Astaxanthin Lutein β carotene Lycopene Fucoxanthin Fucoxanthinol	↑ Social behavior ↑ Memory ↓ Anxiety ↓ Hyperactivity ↓ Aggression ↓ Repetitiveness ↓ Depression ↓ Pain sensation	↑ Cognitive function ↑ Neuronal survival ↑ Neuronal function ↑ Synaptic function NMDA AMPA ↑ BDNF ↑ CREB ↓ ATP P2X7R signal	↓ Seizure frequency ↓ Seizure severity ↑ Seizure onset times	[104,209,210,213,220,221,222,225,231,234,236,242,248,249,250,254,255,256,258,261,263,266,474]
PUFA	Omega 3 (DHA EPA)	↑ Social behavior ↑ Memory ↓ Anxiety ↓ Repetitive behavior ↓ Hyperactivity ↓ Speech problems; ↓ Depression	↑ Neuronal function ↑ Brain development	↓ Seizure frequency ↓ Seizure severity	[242,283,292,296,297,306,308,309,310,311,312,313,323,324,329,331,332,337]
Polyphenols	EGCG Resveratrol Luteolin Fisetin Phlorotannins	↑ Social behavior ↑ Memory ↓ Anxiety ↓ Stereotypy	↑ Synaptic development ↑ Neuronal function ↑ Cognitive function ↑ BDNF	↓ Seizure frequency ↓ Seizure intensity ↓ Seizure onset	[220,342,343,346,348,357,359,362,376,380,384,392,396,398,400,401,404,407,414,475,476,477,478,479]
Vitamins	Vitamin D Vitamin B Vitamin C Vitamin A Vitamin E Vitamin K	↑ Social behavior ↑ Memory ↓ Repetitiveness ↓ ASD symptoms	↑ Serotonin ↑ Dopamine ↑ GABA ↑ Cognitive function ↑ BDNF ↓ Neuronal damage	↓ Seizure frequency ↓ Seizure intensity ↓ Seizure onset	[183,431,432,434,436,437,443,449,454,457,460,462,465,466,467,469]

Abbreviations: ASD, autism spectrum disorder; AMPA, α-amino-3-hydroxy-5-methyl-4-isoxazolepropionic acid receptor; BDNF, brain-derived neu-rotrophic factor; CCL, C-C motif chemokine ligand; GABA, gamma-aminobutyric acid; NMDA, N-methyl-D-aspartate receptor; P2X7R, P2X purinoceptor 7 receptor.

**Table 4:** Summary of algae-derived compound classes and their underlying molecular, biological, and microbial mechanisms in ASD and epilepsy (↑ represents increased outcome, ↓ represents decreased outcome).

Compound Class	Active Compounds	Inflammatory Markers	Oxidative Stress Markers	Signaling Pathways	Microbiome	Refs.
Polysaccharides	Fucoidan β glucan	↑ IL-10 ↓ TNF-α, IL-1β, IL-6 ↓ CCL5, CCL22, CXCL1, CXCL5, CXCL8 ↓ COX 2	↓ NO ↓ iNOS	↓ NF-κB ↓ NLRP3 ↓ MAPK ↓ JAK STAT	↑ *Bacteroides* ↑ *Prevotella* ↑ *Faecalibacterium prausnitzii* ↑ *Prevotella copri* ↓ *Enterobacter* ↓ *Lactobacillus* ↓ *E. coli* ↓ *Akkermansia muciniphila* CAG:154 ↓ *Blautia* spp. ↓ *Coprobacillus* sp. ↓ *Clostridium*	[141,148,149,150,155,156,158,160,161,162,173,181,471,473]
Carotenoids	Astaxanthin Lutein β carotene Lycopene Fucoxanthin Fucoxanthinol	↑ IL-10 ↓ TNF-α, IL-1β, IL-6 ↓ Arginase 1 ↓ VEGF ↓ COX 2	↑ GSH ↓ Oxidative stress ↓ ROS ↓ NOS ↓ iNOS ↓ nNOS ↓ MDA ↑ SOD	↑ PI3K AKT ↑ Nrf2 ↑ PKA CREB ↑ Nrf2 HO 1 ↓ JAK STAT3 ↓ NF-κB ↓ MAPK ↓ Microtubule affinity-regulating kinases	↑ *Akkermansia* ↑ beneficial microbial diversity ↑ *Butyricimonas* ↑ *Faecalibacterium* ↑ *Firmicutes* ↑ *Lachnospiraceae* ↑ *Phascolarctobacterium* ↑ *Parabacteroides* ↑ SCFA ↑ *Ruminococcaceae* ↑ *Subdoligranulum* ↓ *Bacteroidetes* ↓ *Blautia* ↓ *Bilophila* ↓ *Dehalobacterium* ↓ *Ruminococcus* ↓ *Prevotella*	[104,209,210,213,220,221,222,225,231,234,236,242,248,249,250,254,255,256,258,261,263,266,474]
PUFA	Omega 3 (DHA EPA)	↑ IFN-γ ↓ TNF-α ↓ IL-6	↓ NO	↓ NF-κB ↑ PPARγ		[242,283,292,296,297,306,308,309,310,311,312,313,323,324,329,331,332,337]
Polyphenols	EGCG Resveratrol Luteolin Fisetin Phlorotannins	↑ IL-10 ↓ TNF-α, IL-1β, IL-6, IL-4, IL-5, IL-13	↑ GSH ↓ iNOS ↓ ROS ↓ MDA ↓ Glial activation	↑ Nrf2 ↓ NF-κB	↑ *Bifidobacterium* ↑ *Akkermansia* ↓ *Clostridium*	[220,342,343,346,348,357,359,362,376,380,384,392,396,398,400,401,404,407,414,475,476,477,478,479]
Vitamins	Vitamin D Vitamin B Vitamin C Vitamin A Vitamin E Vitamin K	↓ IL-6 ↓ TNF-α ↑ IL-10	↑ SOD ↑ GSH Px ↑ GSH ↓ Myeloperoxidase ↓ MDA		↑ *Bifidobacterium* ↑ *Bacillaceae* ↑ *Firmicutes* ↑ *Anaerotruncus* ↑ *Lactobacillus* ↓ *Desulfovibrio*	[183,431,432,434,436,437,443,449,454,457,460,462,465,466,467,469]

Abbreviations: COX-2, cyclooxygenase-2; CREB, cAMP re-sponse element-binding protein; CXCL, C-X-C motif chemokine ligand; DHA, docosahexaenoic acid; EGCG, epigallocatechin gallate; EPA, eicosapentaenoic acid; GSH, glutathione; GSH-Px, glutathione peroxidase; IFN-γ, interferon-gamma; IL, interleukin; iNOS, inducible nitric oxide synthase; JAK/STAT, Janus kinase/signal transducer and activator of transcription; MAPK, mitogen-activated protein kinase; MDA, malondialdehyde; NF-κB, nuclear factor kappa-light-chain-enhancer of activated B cells; NO, nitric oxide; nNOS, neuronal nitric oxide synthase; NLRP3, NOD-like receptor family pyrin domain containing 3; Nrf2, nuclear factor erythroid 2-related factor 2; PI3K/AKT, phosphoinositide 3-kinase/protein kinase B; PKA, protein kinase A; PSD95, postsynaptic density protein 95; PUFA, polyunsaturated fatty acid; ROS, reactive oxygen species; SCFA, short-chain fatty acids; SOD, superoxide dismutase; TNF-α, tumor necrosis factor-alpha; VEGF, vascular endothelial growth factor.

## Data Availability

No new data were created or analyzed in this study. Data sharing is not applicable to this article.

## Abbreviations

The following abbreviations are used in this manuscript:

ADHD	Attention-deficit hyperactivity disorder	
AEDs	Antiepileptic drugs	
ALA	Alpha-linolenic acid	
ARA	Arachidonic acid	
ASD	Autism spectrum disorder	
BBB	Blood–brain barrier	
BDNF	Brain-derived neurotrophic factor	
CO_2_	Carbon dioxide	
COX-2	Cyclooxygenase-2	
CXCL	C-X-C motif chemokine ligand	
DHA	Docosahexaenoic acid	
EGCG	Epigallocatechin gallate	
EPA	Eicosapentaenoic acid	
GABA	Gamma-aminobutyric acid	
GI	Gastrointestinal	
HO-1	Heme oxygenase-1	
IFN-γ	Interferon-gamma	
IL	Interleukin	
iNOS	Inducible Nitric oxide synthase	
Jak/STAT	Janus kinase/signal transducer and activator of transcription	
MAPK	Mitogen-activated protein kinase	
mTOR	Mechanistic target of rapamycin	
NF-κB	Nuclear factor kappa-light-chain-enhancer of activated B cells	
NMDA	N-methyl-D-aspartate	
nNOS	Neuronal nitric oxide synthase	
Nrf1/2	Nuclear factor erythroid 2-related factor 1/2	
P2X7R	P2X purinoceptor 7 receptor	
PI3K/Akt/GSK-3β	Phosphoinositide 3-kinase/Protein kinase B/Glycogen synthase kinase-3 beta	
PPARγ	Peroxisome proliferator-activated receptor gamma	
PTEN	Phosphatase and tensin homolog	
PTZ	Pentylenetetrazole	
PUFAs	Polyunsaturated fatty acids	
ROS	Reactive oxygen species	
SCFAs	Short-chain fatty acids	
SE	Status epilepticus	
THIK1	Tandem pore domain halothane-inhibited K+ channel 1	
TLR	Toll-like receptor	
TNF	Tumor necrosis factor	
VEGF	Vascular endothelial growth factor	

## References

[1] Weissberg O., Elliott E. (2021). The mechanisms of CHD8 in neurodevelopment and autism spectrum disorders. Genes.

[2] Posar A., Visconti P. (2023). Autism Spectrum Disorder and the Diagnostic and Statistical Manual of Mental Disorders-Fifth Edition (DSM-5): The Experience of 10 Years. Turk. Arch. Pediatr..

[3] Lord C., Bishop S.L. (2015). Recent advances in autism research as reflected in DSM-5 criteria for autism spectrum disorder. Annu. Rev. Clin. Psychol..

[4] Maenner M.J., Warren Z., Williams A.R., Amoakohene E., Bakian A.V., Bilder D.A., Durkin M.S., Fitzgerald R.T., Furnier S.M., Hughes M.M. (2023). Prevalence and characteristics of Autism Spectrum Disorder among children aged 8 years—Autism and developmental disabilities monitoring network, 11 sites, united states, 2020. MMWR Surveill. Summ..

[5] Maenner M.J., Shaw K.A., Bakian A.V., Bilder D.A., Durkin M.S., Esler A., Furnier S.M., Hallas L., Hall-Lande J., Hudson A. (2021). Prevalence and Characteristics of Autism Spectrum Disorder Among Children Aged 8 Years—Autism and Developmental Disabilities Monitoring Network, 11 Sites, United States, 2018. MMWR Surveill. Summ..

[6] Chatterjee D., Maparu K., Singh S. (2025). Exploring pathological targets and advancing pharmacotherapy in autism spectrum disorder: Contributions of glial cells and heavy metals. Histol. Histopathol..

[7] Bulonza R., Watkins K., Parsons R., Sunderland B., Whitehouse A., Caccetta R. (2024). The use of psychotropic medications in autistic individuals (21 years and younger) in Western Australia: A preliminary investigation. Autism.

[8] Weissberg O., Harari R., Dogun C., Getselter D., Elliott E. (2025). CHD8 adulthood microglial knockdown in C57BL6 mice induces behavioral, morphological, and transcriptional changes in a sex-dependent manner. Transl. Psychiatry.

[9] Keil-Stietz K., Lein P.J. (2023). Gene × environment interactions in autism spectrum disorders. Curr. Top. Dev. Biol..

[10] Satterstrom F.K., Kosmicki J.A., Wang J., Breen M.S., De Rubeis S., An J.-Y., Peng M., Collins R., Grove J., Klei L. (2020). Large-Scale Exome Sequencing Study Implicates Both Developmental and Functional Changes in the Neurobiology of Autism. Cell.

[11] Usui N., Kobayashi H., Shimada S. (2023). Neuroinflammation and oxidative stress in the pathogenesis of autism spectrum disorder. Int. J. Mol. Sci..

[12] Bergdolt L., Dunaevsky A. (2019). Brain changes in a maternal immune activation model of neurodevelopmental brain disorders. Prog. Neurobiol..

[13] Lee G.A., Lin Y.-K., Lai J.-H., Lo Y.-C., Yang Y.-C.S.H., Ye S.-Y., Lee C.-J., Wang C.-C., Chiang Y.-H., Tseng S.-H. (2021). Maternal Immune Activation Causes Social Behavior Deficits and Hypomyelination in Male Rat Offspring with an Autism-Like Microbiota Profile. Brain Sci..

[14] Howsmon D.P., Kruger U., Melnyk S., James S.J., Hahn J. (2017). Classification and adaptive behavior prediction of children with autism spectrum disorder based upon multivariate data analysis of markers of oxidative stress and DNA methylation. PLoS Comput. Biol..

[15] Toscano C.V.A., Barros L., Lima A.B., Nunes T., Carvalho H.M., Gaspar J.M. (2021). Neuroinflammation in autism spectrum disorders: Exercise as a “pharmacological” tool. Neurosci. Biobehav. Rev..

[16] Kordulewska N.K., Kostyra E., Piskorz-Ogórek K., Moszyńska M., Cieślińska A., Fiedorowicz E., Jarmołowska B. (2019). Serum cytokine levels in children with spectrum autism disorder: Differences in pro- and anti-inflammatory balance. J. Neuroimmunol..

[17] Vogel Ciernia A., Careaga M., LaSalle J.M., Ashwood P. (2018). Microglia from offspring of dams with allergic asthma exhibit epigenomic alterations in genes dysregulated in autism. Glia.

[18] Than U.T.T., Nguyen L.T., Nguyen P.H., Nguyen X.-H., Trinh D.P., Hoang D.H., Nguyen P.A.T., Dang V.D. (2023). Inflammatory mediators drive neuroinflammation in autism spectrum disorder and cerebral palsy. Sci. Rep..

[19] Xie J., Huang L., Li X., Li H., Zhou Y., Zhu H., Pan T., Kendrick K.M., Xu W. (2017). Immunological cytokine profiling identifies TNF-α as a key molecule dysregulated in autistic children. Oncotarget.

[20] Meng J., Zhang L., Zhang Y.-W. (2024). Microglial dysfunction in autism spectrum disorder. Neuroscientist.

[21] Hughes H.K., Moreno R.J., Ashwood P. (2023). Innate immune dysfunction and neuroinflammation in autism spectrum disorder (ASD). Brain Behav. Immun..

[22] Bjørklund G., Meguid N.A., El-Bana M.A., Tinkov A.A., Saad K., Dadar M., Hemimi M., Skalny A.V., Hosnedlová B., Kizek R. (2020). Oxidative stress in autism spectrum disorder. Mol. Neurobiol..

[23] Atanasova E., Arévalo A.P., Graf I., Zhang R., Bockmann J., Lutz A.-K., Boeckers T.M. (2023). Immune activation during pregnancy exacerbates ASD-related alterations in Shank3-deficient mice. Mol. Autism.

[24] Varghese M., Keshav N., Jacot-Descombes S., Warda T., Wicinski B., Dickstein D.L., Harony-Nicolas H., De Rubeis S., Drapeau E., Buxbaum J.D. (2017). Autism spectrum disorder: Neuropathology and animal models. Acta Neuropathol..

[25] Vuong H.E., Hsiao E.Y. (2017). Emerging roles for the gut microbiome in autism spectrum disorder. Biol. Psychiatry.

[26] Zerbo O., Qian Y., Yoshida C., Grether J.K., Van de Water J., Croen L.A. (2013). Maternal Infection During Pregnancy and Autism Spectrum Disorders. J. Autism Dev. Disord..

[27] Nudel R., Thompson W.K., Børglum A.D., Hougaard D.M., Mortensen P.B., Werge T., Nordentoft M., Benros M.E. (2022). Maternal pregnancy-related infections and autism spectrum disorder-the genetic perspective. Transl. Psychiatry.

[28] Morton J.T., Jin D.-M., Mills R.H., Shao Y., Rahman G., McDonald D., Zhu Q., Balaban M., Jiang Y., Cantrell K. (2023). Multi-level analysis of the gut-brain axis shows autism spectrum disorder-associated molecular and microbial profiles. Nat. Neurosci..

[29] Lasheras I., Real-López M., Santabárbara J. (2023). Prevalence of gastrointestinal symptoms in autism spectrum disorder: A meta-analysis. An. Pediatr..

[30] Paysour M.J., Bolte A.C., Lukens J.R. (2019). Crosstalk Between the Microbiome and Gestational Immunity in Autism-Related Disorders. DNA Cell Biol..

[31] Hao X., Pan J., Gao X., Zhang S., Li Y. (2020). Gut microbiota on gender bias in autism spectrum disorder. Rev. Neurosci..

[32] Srikantha P., Mohajeri M.H. (2019). The Possible Role of the Microbiota-Gut-Brain-Axis in Autism Spectrum Disorder. Int. J. Mol. Sci..

[33] Lewandowska-Pietruszka Z., Figlerowicz M., Mazur-Melewska K. (2023). Microbiota in autism spectrum disorder: A systematic review. Int. J. Mol. Sci..

[34] Agarwala S., Naik B., Ramachandra N.B. (2021). Mucosa-associated specific bacterial species disrupt the intestinal epithelial barrier in the autism phenome. Brain Behav. Immun. Health.

[35] Tomova A., Husarova V., Lakatosova S., Bakos J., Vlkova B., Babinska K., Ostatnikova D. (2015). Gastrointestinal microbiota in children with autism in Slovakia. Physiol. Behav..

[36] Yang J., Fu X., Liao X., Li Y. (2020). Effects of gut microbial-based treatments on gut microbiota, behavioral symptoms, and gastrointestinal symptoms in children with autism spectrum disorder: A systematic review. Psychiatry Res..

[37] Shaaban S.Y., El Gendy Y.G., Mehanna N.S., El-Senousy W.M., El-Feki H.S.A., Saad K., El-Asheer O.M. (2018). The role of probiotics in children with autism spectrum disorder: A prospective, open-label study. Nutr. Neurosci..

[38] Buey B., Forcén A., Grasa L., Layunta E., Mesonero J.E., Latorre E. (2023). Gut Microbiota-Derived Short-Chain Fatty Acids: Novel Regulators of Intestinal Serotonin Transporter. Life.

[39] Boles J.S., Krueger M.E., Jernigan J.E., Cole C.L., Neighbarger N.K., Huarte O.U., Tansey M.G. (2024). A leaky gut dysregulates gene networks in the brain associated with immune activation, oxidative stress, and myelination in a mouse model of colitis. Brain Behav. Immun..

[40] Strandwitz P. (2018). Neurotransmitter modulation by the gut microbiota. Brain Res..

[41] Dicks L.M.T. (2022). Gut bacteria and neurotransmitters. Microorganisms.

[42] Zahra A., Wang Y., Wang Q., Wu J. (2022). Shared Etiology in Autism Spectrum Disorder and Epilepsy with Functional Disability. Behav. Neurol..

[43] Jeste S.S., Tuchman R. (2015). Autism spectrum disorder and epilepsy: Two sides of the same coin?. J. Child Neurol..

[44] Cano-Villagrasa A., Porcar-Gozalbo N., López-Chicheri I., López-Zamora M. (2024). Executive Functioning and Language in a Pediatric Population with Autism Spectrum Disorders and Epilepsy: A Comparative Study. Children.

[45] Liu X., Sun X., Sun C., Zou M., Chen Y., Huang J., Wu L., Chen W.-X. (2022). Prevalence of epilepsy in autism spectrum disorders: A systematic review and meta-analysis. Autism.

[46] Luz-Escamilla L., Morales-González J.A. (2019). Association between Interictal Epileptiform Discharges and Autistic Spectrum Disorder. Brain Sci..

[47] Bosetti C., Ferrini L., Ferrari A.R., Bartolini E., Calderoni S. (2024). Children with Autism Spectrum Disorder and Abnormalities of Clinical EEG: A Qualitative Review. J. Clin. Med..

[48] Santarone M.E., Zambrano S., Zanotta N., Mani E., Minghetti S., Pozzi M., Villa L., Molteni M., Zucca C. (2023). EEG features in autism spectrum disorder: A retrospective analysis in a cohort of preschool children. Brain Sci..

[49] Canitano R., Bozzi Y. (2023). Autism Spectrum Disorder with Epilepsy: A Research Protocol for a Clinical and Genetic Study. Genes.

[50] Frye R.E., Casanova M.F., Fatemi S.H., Folsom T.D., Reutiman T.J., Brown G.L., Edelson S.M., Slattery J.C., Adams J.B. (2016). Neuropathological mechanisms of seizures in autism spectrum disorder. Front. Neurosci..

[51] Betancur C. (2011). Etiological heterogeneity in autism spectrum disorders: More than 100 genetic and genomic disorders and still counting. Brain Res..

[52] Dhawan A., Baitamouni S., Liu D., Busch R., Klaas P., Frazier T.W., Srivastava S., Parikh S., Hsich G.E., Friedman N.R. (2024). Exploring the neurological features of individuals with germline PTEN variants: A multicenter study. Ann. Clin. Transl. Neurol..

[53] Specchio N., Di Micco V., Aronica E., Auvin S., Balestrini S., Brunklaus A., Gardella E., Scheper M., Taglialatela M., Trivisano M. (2025). The epilepsy-autism phenotype associated with developmental and epileptic encephalopathies: New mechanism-based therapeutic options. Epilepsia.

[54] Ahring P.K., Liao V.W.Y., Gardella E., Johannesen K.M., Krey I., Selmer K.K., Stadheim B.F., Davis H., Peinhardt C., Koko M. (2022). Gain-of-function variants in GABRD reveal a novel pathway for neurodevelopmental disorders and epilepsy. Brain.

[55] Johannesen K.M., Gardella E., Linnankivi T., Courage C., Martin A.D.S., Lehesjoki A.-E., Mignot C., Afenjar A., Lesca G., Abi-Warde M.-T. (2018). Defining the phenotypic spectrum of SLC6A1 mutations. Epilepsia.

[56] Fabisiak T., Patel M. (2022). Crosstalk between neuroinflammation and oxidative stress in epilepsy. Front. Cell Dev. Biol..

[57] Matta S.M., Hill-Yardin E.L., Crack P.J. (2019). The influence of neuroinflammation in Autism Spectrum Disorder. Brain Behav. Immun..

[58] Ravizza T., Scheper M., Di Sapia R., Gorter J., Aronica E., Vezzani A. (2024). mTOR and neuroinflammation in epilepsy: Implications for disease progression and treatment. Nat. Rev. Neurosci..

[59] Fleeman N., Bradley P.M., Panebianco M., Sharma A. (2022). Care delivery and self-management strategies for children with epilepsy. Cochrane Database Syst. Rev..

[60] Sarmast S.T., Abdullahi A.M., Jahan N. (2020). Current classification of seizures and epilepsies: Scope, limitations and recommendations for future action. Cureus.

[61] Hawas Y., Abbas A., Alkhawaldeh I.M., Zeid M.A., Azzawi M.A.D.A., Alsalhi H.K., Negida A. (2025). Efficacy and safety of transcranial direct current stimulation (tDCS) in treatment of refractory epilepsy: An updated systematic review and meta-analysis of randomized sham-controlled trials. Neurol. Sci..

[62] Scheffer I.E., Berkovic S., Capovilla G., Connolly M.B., French J., Guilhoto L., Hirsch E., Jain S., Mathern G.W., Moshé S.L. (2017). ILAE classification of the epilepsies: Position paper of the ILAE Commission for Classification and Terminology. Epilepsia.

[63] Nabbout R., Kuchenbuch M. (2020). Impact of predictive, preventive and precision medicine strategies in epilepsy. Nat. Rev. Neurol..

[64] Benbadis S.R., Beniczky S., Bertram E., MacIver S., Moshé S.L. (2020). The role of EEG in patients with suspected epilepsy. Epileptic Disord..

[65] Di Noia S., Bonezzi L., Accorinti I., Bartolini E. (2024). Diagnosis and Classification of Pediatric Epilepsy in Sub-Saharan Africa: A Comprehensive Review. J. Clin. Med..

[66] Žiburkus J., Cressman J.R., Schiff S.J. (2013). Seizures as imbalanced up states: Excitatory and inhibitory conductances during seizure-like events. J. Neurophysiol..

[67] van van Hugte E.J.H., Schubert D., NadifKasri N. (2023). Excitatory/inhibitory balance in epilepsies and neurodevelopmental disorders: Depolarizing γ-aminobutyric acid as a common mechanism. Epilepsia.

[68] Singh S., Singh T.G., Rehni A.K. (2020). An Insight into Molecular Mechanisms and Novel Therapeutic Approaches in Epileptogenesis. CNS Neurol. Disord. Drug Targets.

[69] Hodges S.L., Lugo J.N. (2020). Therapeutic role of targeting mTOR signaling and neuroinflammation in epilepsy. Epilepsy Res..

[70] Clossen B.L., Reddy D.S. (2017). Novel therapeutic approaches for disease-modification of epileptogenesis for curing epilepsy. Biochim. Biophys. Acta Mol. Basis Dis..

[71] Löscher W., Howe C.L. (2022). Molecular mechanisms in the genesis of seizures and epilepsy associated with viral infection. Front. Mol. Neurosci..

[72] DePaula-Silva A.B., Bell L.A., Wallis G.J., Wilcox K.S. (2021). Inflammation Unleashed in Viral-Induced Epileptogenesis. Epilepsy Curr..

[73] Sánchez J.D., Gómez-Carpintero J., González J.F., Menéndez J.C. (2024). Twenty-first century antiepileptic drugs. An overview of their targets and synthetic approaches. Eur. J. Med. Chem..

[74] Ghosh S., Sinha J.K., Khan T., Devaraju K.S., Singh P., Vaibhav K., Gaur P. (2021). Pharmacological and therapeutic approaches in the treatment of epilepsy. Biomedicines.

[75] Borowicz-Reutt K., Krawczyk M., Czernia J. (2024). Ketogenic diet in the treatment of epilepsy. Nutrients.

[76] Klein P., Kaminski R.M., Koepp M., Löscher W. (2024). New epilepsy therapies in development. Nat. Rev. Drug Discov..

[77] Ghosh S., Sinha J.K., Ghosh S., Sharma H., Bhaskar R., Narayanan K.B. (2023). A comprehensive review of emerging trends and innovative therapies in epilepsy management. Brain Sci..

[78] Taha M., Nordli D.R., Park C., Nordli D.R. (2025). Innovative epilepsy management: A combined figure of EEG categorization and medication mechanisms. Front. Neurol..

[79] Amlerova J., Šroubek J., Angelucci F., Hort J. (2021). Evidences for a role of gut microbiota in pathogenesis and management of epilepsy. Int. J. Mol. Sci..

[80] Dinan T.G., Stanton C., Cryan J.F. (2013). Psychobiotics: A novel class of psychotropic. Biol. Psychiatry.

[81] Yano J.M., Yu K., Donaldson G.P., Shastri G.G., Ann P., Ma L., Nagler C.R., Ismagilov R.F., Mazmanian S.K., Hsiao E.Y. (2015). Indigenous bacteria from the gut microbiota regulate host serotonin biosynthesis. Cell.

[82] Suganya K., Koo B.-S. (2020). Gut-Brain Axis: Role of Gut Microbiota on Neurological Disorders and How Probiotics/Prebiotics Beneficially Modulate Microbial and Immune Pathways to Improve Brain Functions. Int. J. Mol. Sci..

[83] Braniste V., Al-Asmakh M., Kowal C., Anuar F., Abbaspour A., Tóth M., Korecka A., Bakocevic N., Ng L.G., Kundu P. (2014). The gut microbiota influences blood-brain barrier permeability in mice. Sci. Transl. Med..

[84] Heiss C.N., Olofsson L.E. (2019). The role of the gut microbiota in development, function and disorders of the central nervous system and the enteric nervous system. J. Neuroendocrinol..

[85] Holmes M., Flaminio Z., Vardhan M., Xu F., Li X., Devinsky O., Saxena D. (2020). Cross talk between drug-resistant epilepsy and the gut microbiome. Epilepsia.

[86] Chatzikonstantinou S., Gioula G., Kimiskidis V.K., McKenna J., Mavroudis I., Kazis D. (2021). The gut microbiome in drug-resistant epilepsy. Epilepsia Open.

[87] Peng A., Qiu X., Lai W., Li W., Zhang L., Zhu X., He S., Duan J., Chen L. (2018). Altered composition of the gut microbiome in patients with drug-resistant epilepsy. Epilepsy Res..

[88] Gong X., Liu X., Chen C., Lin J., Li A., Guo K., An D., Zhou D., Hong Z. (2020). Alteration of gut microbiota in patients with epilepsy and the potential index as a biomarker. Front. Microbiol..

[89] Dahlin M., Prast-Nielsen S. (2019). The gut microbiome and epilepsy. EBioMedicine.

[90] Darch H., McCafferty C.P. (2022). Gut microbiome effects on neuronal excitability & activity: Implications for epilepsy. Neurobiol. Dis..

[91] Kim S., Park S., Choi T.G., Kim S.S. (2022). Role of short chain fatty acids in epilepsy and potential benefits of probiotics and prebiotics: Targeting “health” of epileptic patients. Nutrients.

[92] Silva Y.P., Bernardi A., Frozza R.L. (2020). The Role of Short-Chain Fatty Acids From Gut Microbiota in Gut-Brain Communication. Front. Endocrinol..

[93] Wang X., Ma R., Liu X., Zhang Y. (2022). Effects of long-term supplementation of probiotics on cognitive function and emotion in temporal lobe epilepsy. Front. Neurol..

[94] Gómez-Eguílaz M., Ramón-Trapero J.L., Pérez-Martínez L., Blanco J.R. (2018). The beneficial effect of probiotics as a supplementary treatment in drug-resistant epilepsy: A pilot study. Benef. Microbes.

[95] El-Ansary A., Alhakbany M., Alfawaz H.A., Al-Ayadhi L.Y. (2024). Indian Hedgehog (IHh) Protein and COX-2 as Biomarkers to Define the Mechanism of Epilepsy and Gastrointestinal Problems as Comorbid Medical Illnesses in Autism Spectrum Disorder: Combining ROC Curves to Improve Diagnostic Values. J. Clin. Med..

[96] Giliberti A., Frisina A.M., Giustiniano S., Carbonaro Y., Roccella M., Nardello R. (2025). Autism spectrum disorder and epilepsy: Pathogenetic mechanisms and therapeutic implications. J. Clin. Med..

[97] Ding M., Lang Y., Shu H., Shao J., Cui L. (2021). Microbiota-Gut-Brain Axis and Epilepsy: A Review on Mechanisms and Potential Therapeutics. Front. Immunol..

[98] Saurman V., Margolis K.G., Luna R.A. (2020). Autism Spectrum Disorder as a Brain-Gut-Microbiome Axis Disorder. Dig. Dis. Sci..

[99] Yue Q., Cai M., Xiao B., Zhan Q., Zeng C. (2022). The Microbiota-Gut-Brain Axis and Epilepsy. Cell. Mol. Neurobiol..

[100] Socała K., Doboszewska U., Szopa A., Serefko A., Włodarczyk M., Zielińska A., Poleszak E., Fichna J., Wlaź P. (2021). The role of microbiota-gut-brain axis in neuropsychiatric and neurological disorders. Pharmacol. Res..

[101] Strzelczyk A., Schubert-Bast S. (2022). Psychobehavioural and Cognitive Adverse Events of Anti-Seizure Medications for the Treatment of Developmental and Epileptic Encephalopathies. CNS Drugs.

[102] Valabhji J., Barron E., Pratt A., Hafezparast N., Dunbar-Rees R., Turner E.B., Roberts K., Mathews J., Deegan R., Cornelius V. (2024). Prevalence of multiple long-term conditions (multimorbidity) in England: A whole population study of over 60 million people. J. R. Soc. Med..

[103] Cano-Villagrasa A., Moya-Faz F.J., Porcar-Gozalbo N., López-Zamora M. (2024). Treatment options in autism with epilepsy. Front. Child Adolesc. Psychiatry.

[104] Hannan M.A., Dash R., Haque M.N., Mohibbullah M., Sohag A.A.M., Rahman M.A., Uddin M.J., Alam M., Moon I.S. (2020). Neuroprotective potentials of marine algae and their bioactive metabolites: Pharmacological insights and therapeutic advances. Mar. Drugs.

[105] Barbalace M.C., Malaguti M., Giusti L., Lucacchini A., Hrelia S., Angeloni C. (2019). Anti-Inflammatory Activities of Marine Algae in Neurodegenerative Diseases. Int. J. Mol. Sci..

[106] Barbosa M., Valentão P., Andrade P.B. (2014). Bioactive compounds from macroalgae in the new millennium: Implications for neurodegenerative diseases. Mar. Drugs.

[107] Li C.-Q., Ma Q.-Y., Gao X.-Z., Wang X., Zhang B.-L. (2021). Research Progress in Anti-Inflammatory Bioactive Substances Derived from Marine Microorganisms, Sponges, Algae, and Corals. Mar. Drugs.

[108] Michalak I., Chojnacka K. (2015). Algae as production systems of bioactive compounds. Eng. Life Sci..

[109] Martins B., Vieira M., Delerue-Matos C., Grosso C., Soares C. (2022). Biological Potential, Gastrointestinal Digestion, Absorption, and Bioavailability of Algae-Derived Compounds with Neuroprotective Activity: A Comprehensive Review. Mar. Drugs.

[110] Barbosa F., Pinto E., Kijjoa A., Pinto M., Sousa E. (2020). Targeting antimicrobial drug resistance with marine natural products. Int. J. Antimicrob. Agents.

[111] Gnanavel V., Roopan S.M., Rajeshkumar S. (2019). Aquaculture: An overview of chemical ecology of seaweeds (food species) in natural products. Aquaculture.

[112] Lourenço-Lopes C., Fraga-Corral M., Jimenez-Lopez C., Pereira A.G., Garcia-Oliveira P., Carpena M., Prieto M.A., Simal-Gandara J. (2020). Metabolites from Macroalgae and Its Applications in the Cosmetic Industry: A Circular Economy Approach. Resources.

[113] Silva A., Silva S.A., Carpena M., Garcia-Oliveira P., Gullón P., Barroso M.F., Prieto M., Simal-Gandara J. (2020). Macroalgae as a source of valuable antimicrobial compounds: Extraction and applications. Antibiotics.

[114] Batista A.P., Niccolai A., Fradinho P., Fragoso S., Bursic I., Rodolfi L., Biondi N., Tredici M.R., Sousa I., Raymundo A. (2017). Microalgae biomass as an alternative ingredient in cookies: Sensory, physical and chemical properties, antioxidant activity and in vitro digestibility. Algal Res..

[115] Ahmad S., Singh A., Akram W., Upadhyay A., Abrol G.S. (2025). Algal lipids: A review on current status and future prospects in food processing. J. Food Sci..

[116] Pereira L. (2021). Macroalgae. Encyclopedia.

[117] Spalding H.L., Amado-Filho G.M., Bahia R.G., Ballantine D.L., Fredericq S., Leichter J.J., Nelson W.A., Slattery M., Tsuda R.T., Loya Y., Puglise K.A., Bridge T.C.L. (2019). Macroalgae. Mesophotic Coral Ecosystems.

[118] Pepper I.L., Gentry T.J. (2015). Microorganisms found in the environment. Environmental Microbiology.

[119] Mirzapour-Kouhdasht A., Garcia-Vaquero M., Huang J.-Y. (2024). Algae-derived compounds: Bioactivity, allergenicity and technologies enhancing their values. Bioresour. Technol..

[120] Praveen M.A., Parvathy K.R.K., Balasubramanian P., Jayabalan R. (2019). An overview of extraction and purification techniques of seaweed dietary fibers for immunomodulation on gut microbiota. Trends Food Sci. Technol..

[121] Anyanwu R.C., Rodriguez C., Durrant A., Olabi A.G. (2018). Micro-Macroalgae Properties and Applications. Reference Module in Materials Science and Materials Engineering.

[122] Zhang R., Parniakov O., Grimi N., Lebovka N., Marchal L., Vorobiev E. (2019). Emerging techniques for cell disruption and extraction of valuable bio-molecules of microalgae *Nannochloropsis* sp. *Bioprocess*. Biosyst. Eng..

[123] Osuala O.J., Ezemba C.C. (2022). Algae, a hope for sustainable living. Asian J. Res. Bot..

[124] Ratnapuram H.P., Vutukuru S.S., Yadavalli R. (2018). Mixotrophic transition induced lipid productivity in Chlorella pyrenoidosa under stress conditions for biodiesel production. Heliyon.

[125] Vu C.H.T., Lee H.-G., Chang Y.K., Oh H.-M. (2018). Axenic cultures for microalgal biotechnology: Establishment, assessment, maintenance, and applications. Biotechnol. Adv..

[126] Caroppo C., Pagliara P. (2022). Microalgae: A promising future. Microorganisms.

[127] Gamal R., Shreadah M.A. (2024). Marine microalgae and their industrial biotechnological applications: A review. J. Genet. Eng. Biotechnol..

[128] Guo J., Qi M., Chen H., Zhou C., Ruan R., Yan X., Cheng P. (2022). Macroalgae-Derived Multifunctional Bioactive Substances: The Potential Applications for Food and Pharmaceuticals. Foods.

[129] Zhou L., Li K., Duan X., Hill D., Barrow C., Dunshea F., Martin G., Suleria H. (2022). Bioactive compounds in microalgae and their potential health benefits. Food Biosci..

[130] Chen X., Yang J., Shen M., Chen Y., Yu Q., Xie J. (2022). Structure, function and advance application of microwave-treated polysaccharide: A review. Trends Food Sci. Technol..

[131] Andryukov B.G., Besednova N.N., Kuznetsova T.A., Zaporozhets T.S., Ermakova S.P., Zvyagintseva T.N., Chingizova E.A., Gazha A.K., Smolina T.P. (2020). Sulfated Polysaccharides from Marine Algae as a Basis of Modern Biotechnologies for Creating Wound Dressings: Current Achievements and Future Prospects. Biomedicines.

[132] Mišurcová L., Škrovánková S., Samek D., Ambrožová J., Machů L. (2012). Health benefits of algal polysaccharides in human nutrition. Adv. Food Nutr. Res..

[133] Geetha Bai R., Tuvikene R. (2021). Potential antiviral properties of industrially important marine algal polysaccharides and their significance in fighting a future viral pandemic. Viruses.

[134] Mohammed A.S.A., Naveed M., Jost N. (2021). Polysaccharides; classification, chemical properties, and future perspective applications in fields of pharmacology and biological medicine (A review of current applications and upcoming potentialities). J. Polym. Environ..

[135] Otero P., Carpena M., Garcia-Oliveira P., Echave J., Soria-Lopez A., Garcia-Perez P., Fraga-Corral M., Cao H., Nie S., Xiao J. (2023). Seaweed polysaccharides: Emerging extraction technologies, chemical modifications and bioactive properties. Crit. Rev. Food Sci. Nutr..

[136] Hu D., Cheong K., Zhao J., Li S. (2013). Chromatography in characterization of polysaccharides from medicinal plants and fungi. J. Sep. Sci..

[137] Besednova N.N., Zaporozhets T.S., Kuznetsova T.A., Makarenkova I.D., Kryzhanovsky S.P., Fedyanina L.N., Ermakova S.P. (2020). Extracts and marine algae polysaccharides in therapy and prevention of inflammatory diseases of the intestine. Mar. Drugs.

[138] Cunha L., Grenha A. (2016). Sulfated seaweed polysaccharides as multifunctional materials in drug delivery applications. Mar. Drugs.

[139] Liyanage N.M., Nagahawatta D.P., Jayawardena T.U., Jeon Y.-J. (2023). The Role of Seaweed Polysaccharides in Gastrointestinal Health: Protective Effect against Inflammatory Bowel Disease. Life.

[140] Sepe F., Valentino A., Marcolongo L., Petillo O., Calarco A., Margarucci S., Peluso G., Conte R. (2025). Polysaccharide hydrogels as delivery platforms for natural bioactive molecules: From tissue regeneration to infection control. Gels.

[141] Guo R., Pang J., Zhao J., Xiao X., Li J., Li J., Wang W., Zhou S., Zhao Y., Zhang Z. (2023). Unveiling the neuroprotective potential of dietary polysaccharides: A systematic review. Front. Nutr..

[142] Lomartire S., Gonçalves A.M.M. (2023). Algal phycocolloids: Bioactivities and pharmaceutical applications. Mar. Drugs.

[143] Jang H.J., Lee N.-K., Paik H.-D. (2024). A narrative review on the advance of probiotics to metabiotics. J. Microbiol. Biotechnol..

[144] Kamimura K., Maeda N. (2021). Glypicans and heparan sulfate in synaptic development, neural plasticity, and neurological disorders. Front. Neural Circuits.

[145] Dowling C., Allen N.J. (2018). Mice lacking glypican 4 display juvenile hyperactivity and adult social interaction deficits. Brain Plast..

[146] Hope K.A., Flatten D., Cavitch P., May B., Sutcliffe J.S., O’donnell J., Reiter L.T. (2019). The Drosophila Gene Sulfateless Modulates Autism-Like Behaviors. Front. Genet..

[147] Sha A., Chen H., Zhao X. (2024). Exploration of the mechanisms of improving learning and memory in the offspring of aging pregnant mice by supplementation with Paris polyphylla polysaccharide based on the P19-P53-P21 and Wnt/β-catenin signaling pathways. J. Ethnopharmacol..

[148] Raghavan K., Dedeepiya V.D., Yamamoto N., Ikewaki N., Sonoda T., Iwasaki M., Kandaswamy R.S., Senthilkumar R., Preethy S., Abraham S.J. (2023). Benefits of Gut Microbiota Reconstitution by Beta 1,3-1,6 Glucans in Subjects with Autism Spectrum Disorder and Other Neurodegenerative Diseases. J. Alzheimers Dis..

[149] Raghavan K., Dedeepiya V.D., Ikewaki N., Sonoda T., Iwasaki M., Preethy S., Abraham S.J. (2022). Improvement of behavioural pattern and alpha-synuclein levels in autism spectrum disorder after consumption of a beta-glucan food supplement in a randomised, parallel-group pilot clinical study. BMJ Neurol. Open.

[150] Raghavan K., Dedeepiya V.D., Kandaswamy R.S., Balamurugan M., Ikewaki N., Sonoda T., Kurosawa G., Iwasaki M., Preethy S., Abraham S.J. (2022). Improvement of sleep and melatonin in children with autism spectrum disorder after β-1,3/1,6-glucan consumption: An open-label prospective pilot clinical study. Brain Behav..

[151] Kim H.I., Kim D.-S., Jung Y., Sung N.-Y., Kim M., Han I.-J., Nho E.Y., Hong J.H., Lee J.-K., Boo M. (2022). Immune-Enhancing Effect of Sargassum horneri on Cyclophosphamide-Induced Immunosuppression in BALB/c Mice and Primary Cultured Splenocytes. Molecules.

[152] Herath K.H.I.N.M., Kim H.J., Lee J.H., Je J.G., Yu H.-S., Jeon Y.-J., Kim H.J., Jee Y. (2021). Sargassum horneri (Turner) C. Agardh containing polyphenols attenuates particulate matter-induced inflammatory response by blocking TLR-mediated MYD88-dependent MAPK signaling pathway in MLE-12 cells. J. Ethnopharmacol..

[153] Woo G.-E., Hwang H.-J., Park A.-Y., Sim J.-Y., Woo S.-Y., Kim M.-J., Jeong S.-M., Sung N.-Y., Kim D.-S., Ahn D.-H. (2023). Anti-Atopic Activities of Sargassum horneri Hot Water Extracts in 2,4-Dinitrochlorobezene-Induced Mouse Models. J. Microbiol. Biotechnol..

[154] Sanjeewa K.A., Jayawardena T.U., Kim S.-Y., Lee H.G., Je J.-G., Jee Y., Jeon Y.-J. (2020). Sargassum horneri (Turner) inhibit urban particulate matter-induced inflammation in MH-S lung macrophages via blocking TLRs mediated NF-κB and MAPK activation. J. Ethnopharmacol..

[155] Ye J., Chen D., Ye Z., Huang Y., Zhang N., Lui E.M.K., Xue C., Xiao M. (2020). Fucoidan Isolated from Saccharina japonica Inhibits LPS-Induced Inflammation in Macrophages via Blocking NF-κB, MAPK and JAK-STAT Pathways. Mar. Drugs.

[156] Dutot M., Grassin-Delyle S., Salvator H., Brollo M., Rat P., Fagon R., Naline E., Devillier P. (2019). A marine-sourced fucoidan solution inhibits Toll-like-receptor-3-induced cytokine release by human bronchial epithelial cells. Int. J. Biol. Macromol..

[157] Pozharitskaya O.N., Obluchinskaya E.D., Shikov A.N. (2020). Mechanisms of Bioactivities of Fucoidan from the Brown Seaweed Fucus vesiculosus L. of the Barents Sea. Mar. Drugs.

[158] Hu P., Li Z.X., Chen M.C., Sun Z.L., Ling Y., Jiang J., Huang C.G. (2016). Structural elucidation and protective role of a polysaccharide from Sargassum fusiforme on ameliorating learning and memory deficiencies in mice. Carbohydr. Polym..

[159] dos Santos T.C., Obando J.M.C., Leite P.E.C., Pereira M.R., Leitão M.d.F., Abujadi C., Pimenta L.d.F.L., Martins R.C.C., Cavalcanti D.N. (2024). Approaches of marine compounds and relevant immune mediators in Autism Spectrum Disorder: Opportunities and challenges. Eur. J. Med. Chem..

[160] Jiang Y., Wang Z., Wang W., Liu Y., Meng Y., Wang Y., Fan M., Cai C. (2025). Ganoderma lucidum polysaccharide alleviates cognitive dysfunction by inhibiting neuroinflammation via NLRP3/NF-κB signaling pathway. J. Ethnopharmacol..

[161] Hu M., Zhang P., Wang R., Zhou M., Pang N., Cui X., Ge X., Liu X., Huang X.-F., Yu Y. (2022). Three Different Types of β-Glucans Enhance Cognition: The Role of the Gut-Brain Axis. Front. Nutr..

[162] Gall A.J., Griffin G.D. (2021). Anxiolytic effects of administration of a commercially available prebiotic blend of galacto-oligosaccharides and beta glucans in Sprague-Dawley rats. Benef. Microbes.

[163] Singh R.P., Bhardwaj A. (2023). β-glucans: A potential source for maintaining gut microbiota and the immune system. Front. Nutr..

[164] Bobadilla F., Rodriguez-Tirado C., Imarai M., Galotto M.J., Andersson R. (2013). Soluble β-1,3/1,6-glucan in seaweed from the southern hemisphere and its immunomodulatory effect. Carbohydr. Polym..

[165] Camacho F., Macedo A., Malcata F. (2019). Potential industrial applications and commercialization of microalgae in the functional food and feed industries: A short review. Mar. Drugs.

[166] Kumar V., Bhoyar M.S., Mohanty C.S., Chauhan P.S., Toppo K., Ratha S.K. (2025). Untapping the potential of algae for β-glucan production: A review of biological properties, strategies for enhanced production and future perspectives. Carbohydr. Polym..

[167] Sorrenti V., Castagna D.A., Fortinguerra S., Buriani A., Scapagnini G., Willcox D.C. (2021). Spirulina microalgae and brain health: A scoping review of experimental and clinical evidence. Mar. Drugs.

[168] Reilly P., O’Doherty J.V., Pierce K.M., Callan J.J., O’Sullivan J.T., Sweeney T. (2008). The effects of seaweed extract inclusion on gut morphology, selected intestinal microbiota, nutrient digestibility, volatile fatty acid concentrations and the immune status of the weaned pig. Animal.

[169] Yu T., Wang Y., Chen X., Xiong W., Tang Y., Lin L. (2020). Spirulina platensis alleviates chronic inflammation with modulation of gut microbiota and intestinal permeability in rats fed a high-fat diet. J. Cell. Mol. Med..

[170] Pereira L., Valado A. (2021). The seaweed diet in prevention and treatment of the neurodegenerative diseases. Mar. Drugs.

[171] Cornish M.L., Critchley A.T., Mouritsen O.G. (2017). Consumption of seaweeds and the human brain. J. Appl. Phycol..

[172] Xie X., Wu Y., Xie H., Wang H., Zhang X., Yu J., Zhu S., Zhao J., Sui L., Li S. (2022). Polysaccharides, next potential agent for the treatment of epilepsy?. Front. Pharmacol..

[173] Li X., Liu Y., Wang S., Jiang Y., Algradi A.M., Zhou Y., Guan W., Pan J., Kuang H., Yang B. (2022). The Polysaccharides from the Aerial Parts of Bupleurum chinense DC Attenuate Epilepsy-Like Behavior through Oxidative Stress Signaling Pathways. Oxid. Med. Cell. Longev..

[174] Xi S., Li Y., Yue L., Gong Y., Qian L., Liang T., Ye Y. (2020). Role of traditional chinese medicine in the management of viral pneumonia. Front. Pharmacol..

[175] Mittal R., Debs L.H., Patel A.P., Nguyen D., Patel K., O’Connor G., Grati M., Mittal J., Yan D., Eshraghi A.A. (2017). Neurotransmitters: The Critical Modulators Regulating Gut-Brain Axis. J. Cell. Physiol..

[176] Wang Y., Sun M., Jin H., Yang J., Kang S., Liu Y., Yang S., Ma S., Ni J. (2021). Effects of Lycium barbarum Polysaccharides on Immunity and the Gut Microbiota in Cyclophosphamide-Induced Immunosuppressed Mice. Front. Microbiol..

[177] Khan S.A., Nidhi F., Leal A.F., Celik B., Herreño-Pachón A.M., Saikia S., Benincore-Flórez E., Ago Y., Tomatsu S. (2024). Glycosaminoglycans in mucopolysaccharidoses and other disorders. Adv. Clin. Chem..

[178] Xu H., Wang Y.-C., Li X., Wu X., Lu L.W., He J., Wang M., Liu B., Xu H., Chen J.-H. (2024). 1,3-glucans-richsupplementation alleviates high-fat-high-fructose diet-induced cognitive impairment in middle-aged mice via the gut-brain axis. Food Sci. Hum. Wellness.

[179] Bisht D., Kumar D., Kumar D., Dua K., Chellappan D.K. (2021). Phytochemistry and pharmacological activity of the genus artemisia. Arch. Pharm. Res..

[180] Chu Z., Sun Q., Mao M., Wu Y., Yu L., Xu J., Liu K., Qin L., Zhu B. (2025). Pharmacology, phytochemistry, and traditional uses of Huperzia serrata (Thunb. ex Murray) Trev. Fitoterapia.

[181] Ahmad R., Riaz M., Khan A., Aljamea A., Algheryafi M., Sewaket D., Alqathama A. (2021). Ganoderma lucidum (Reishi) an edible mushroom; a comprehensive and critical review of its nutritional, cosmeceutical, mycochemical, pharmacological, clinical, and toxicological properties. Phytother. Res..

[182] Sun X., Jia B., Sun J., Lin J., Lu B., Duan J., Li C., Wang Q., Zhang X., Tan M. (2023). Gastrodia elata Blume: A review of its mechanisms and functions on cardiovascular systems. Fitoterapia.

[183] Lu T., Chen X., Zhang Q., Shang K., Yang X., Xiang W. (2024). Vitamin D Relieves Epilepsy Symptoms and Neuroinflammation in Juvenile Mice by Activating the mTOR Signaling Pathway via RAF1: Insights from Network Pharmacology and Molecular Docking Studies. Neurochem. Res..

[184] Wu J.Y., Tso R., Teo H.S., Haldar S. (2023). The utility of algae as sources of high value nutritional ingredients, particularly for alternative/complementary proteins to improve human health. Front. Nutr..

[185] Yang J., Ran M., Li H., Lin Y., Ma K., Yang Y., Fu X., Yang S. (2022). New insight into neurological degeneration: Inflammatory cytokines and blood-brain barrier. Front. Mol. Neurosci..

[186] Beurel E., Grieco S.F., Jope R.S. (2015). Glycogen synthase kinase-3 (GSK3): Regulation, actions, and diseases. Pharmacol. Ther..

[187] Li J., Ma S., Chen J., Hu K., Li Y., Zhang Z., Su Z., Woodgett J.R., Li M., Huang Q. (2020). GSK-3β Contributes to Parkinsonian Dopaminergic Neuron Death: Evidence From Conditional Knockout Mice and Tideglusib. Front. Mol. Neurosci..

[188] Li Y., Liu T., Li Y., Han D., Hong J., Yang N., He J., Peng R., Mi X., Kuang C. (2020). Baicalin Ameliorates Cognitive Impairment and Protects Microglia from LPS-Induced Neuroinflammation via the SIRT1/HMGB1 Pathway. Oxid. Med. Cell. Longev..

[189] Polyakov N.E., Focsan A.L., Gao Y., Kispert L.D. (2023). The Endless World of Carotenoids-Structural, Chemical and Biological Aspects of Some Rare Carotenoids. Int. J. Mol. Sci..

[190] Galasso C., Corinaldesi C., Sansone C. (2017). Carotenoids from Marine Organisms: Biological Functions and Industrial Applications. Antioxidants.

[191] Viera I., Pérez-Gálvez A., Roca M. (2018). Bioaccessibility of marine carotenoids. Mar. Drugs.

[192] Novoveská L., Ross M.E., Stanley M.S., Pradelles R., Wasiolek V., Sassi J.-F. (2019). Microalgal carotenoids: A review of production, current markets, regulations, and future direction. Mar. Drugs.

[193] Duan X., Xie C., Hill D.R.A., Barrow C.J., Dunshea F.R., Martin G.J.O., Suleria H.A. (2023). Bioaccessibility, bioavailability and bioactivities of carotenoids in microalgae: A review. Food Rev. Int..

[194] Pereira L., Cotas J., Valado A. (2024). Antioxidants from microalgae and their potential impact on human well-being. Explor. Drug Sci..

[195] Foong L.C., Loh C.W.L., Ng H.S., Lan J.C.-W. (2021). Recent development in the production strategies of microbial carotenoids. World J. Microbiol. Biotechnol..

[196] Tanguy G., Legat A., Gonçalves O., Marchal L., Schoefs B. (2021). Selection of Culture Conditions and Cell Morphology for Biocompatible Extraction of β-Carotene from Dunaliella salina. Mar. Drugs.

[197] Chen W., Chen G. (2014). The Roles of Vitamin A in the Regulation of Carbohydrate, Lipid, and Protein Metabolism. J. Clin. Med..

[198] Marcelino G., Machate D.J., Freitas K.d.C., Hiane P.A., Maldonade I.R., Pott A., Asato M.A., Candido C.J., Guimarães R.d.C.A. (2020). β-Carotene: Preventive Role for Type 2 Diabetes Mellitus and Obesity: A Review. Molecules.

[199] Riccio G., Lauritano C. (2019). Microalgae with Immunomodulatory Activities. Mar. Drugs.

[200] Martínez Andrade K.A., Lauritano C., Romano G., Ianora A. (2018). Marine Microalgae with Anti-Cancer Properties. Mar. Drugs.

[201] Ambati R.R., Gogisetty D., Aswathanarayana R.G., Ravi S., Bikkina P.N., Bo L., Yuepeng S. (2019). Industrial potential of carotenoid pigments from microalgae: Current trends and future prospects. Crit. Rev. Food Sci. Nutr..

[202] Wang J., Hu X., Chen J., Wang T., Huang X., Chen G. (2022). The Extraction of β-Carotene from Microalgae for Testing Their Health Benefits. Foods.

[203] Böhm V., Lietz G., Olmedilla-Alonso B., Phelan D., Reboul E., Bánati D., Borel P., Corte-Real J., de Lera A.R., Desmarchelier C. (2021). From carotenoid intake to carotenoid blood and tissue concentrations—Implications for dietary intake recommendations. Nutr. Rev..

[204] Kakarla R., Karuturi P., Siakabinga Q., Viswanath M.K., Dumala N., Guntupalli C., Nalluri B.N., Venkateswarlu K., Prasanna V.S., Gutti G. (2024). Current understanding and future directions of cruciferous vegetables and their phytochemicals to combat neurological diseases. Phytother. Res..

[205] Jeon S., Ranard K.M., Neuringer M., Johnson E.E., Renner L., Kuchan M.J., Pereira S.L., Johnson E.J., Erdman J.W. (2018). Lutein Is Differentially Deposited across Brain Regions following Formula or Breast Feeding of Infant Rhesus Macaques. J. Nutr..

[206] Mrowicka M., Mrowicki J., Kucharska E., Majsterek I. (2022). Lutein and Zeaxanthin and Their Roles in Age-Related Macular Degeneration-Neurodegenerative Disease. Nutrients.

[207] Eggersdorfer M., Wyss A. (2018). Carotenoids in human nutrition and health. Arch. Biochem. Biophys..

[208] Lakey-Beitia J., Kumar D.J., Hegde M.L., Rao K.S. (2019). Carotenoids as novel therapeutic molecules against neurodegenerative disorders: Chemistry and molecular docking analysis. Int. J. Mol. Sci..

[209] Viana C.E., Bortolotto V.C., Araujo S.M., Dahleh M.M.M., Machado F.R., Pereira A.d.S., de Oliveira B.P.M., Leimann F.V., Gonçalves O.H., Prigol M. (2023). Lutein-loaded nanoparticles reverse oxidative stress, apoptosis, and autism spectrum disorder-like behaviors induced by prenatal valproic acid exposure in female rats. Neurotoxicology.

[210] Janner D.E., Poetini M.R., Musachio E.A.S., Chaves N.S.G., Meichtry L.B., Fernandes E.J., Mustafa M.M.D., De Carvalho A.S., Gonçalves O.H., Leimann F.V. (2024). Neurodevelopmental changes in Drosophila melanogaster are restored by treatment with lutein-loaded nanoparticles: Positive modulation of neurochemical and behavioral parameters. Comp. Biochem. Physiol. C Toxicol. Pharmacol..

[211] Martin M., Boulaire M., Lucas C., Peltier A., Pourtau L., Gaudout D., Layé S., Pallet V., Joffre C., Dinel A.-L. (2024). Plant Extracts and ω-3 Improve Short-Term Memory and Modulate the Microbiota-Gut-Brain Axis in D-galactose Model Mice. J. Nutr..

[212] Yeo J. (2023). Influence of food-derived bioactives on gut microbiota compositions and their metabolites by focusing on neurotransmitters. Food Sci. Biotechnol..

[213] Li R., Li L., Hong P., Lang W., Hui J., Yang Y., Zheng X. (2021). β-Carotene prevents weaning-induced intestinal inflammation by modulating gut microbiota in piglets. Anim. Biosci..

[214] Bohn T., Balbuena E., Ulus H., Iddir M., Wang G., Crook N., Eroglu A. (2023). Carotenoids in Health as Studied by Omics-Related Endpoints. Adv. Nutr..

[215] Magbanua M.J.M., Roy R., Sosa E.V., Weinberg V., Federman S., Mattie M.D., Hughes-Fulford M., Simko J., Shinohara K., Haqq C.M. (2011). Gene expression and biological pathways in tissue of men with prostate cancer in a randomized clinical trial of lycopene and fish oil supplementation. PLoS ONE.

[216] Sun S., Cao C., Li J., Meng Q., Cheng B., Shi B., Shan A. (2021). Lycopene Modulates Placental Health and Fetal Development Under High-Fat Diet During Pregnancy of Rats. Mol. Nutr. Food Res..

[217] Eroglu A., Al’Abri I.S., Kopec R.E., Crook N., Bohn T. (2023). Carotenoids and Their Health Benefits as Derived via Their Interactions with Gut Microbiota. Adv. Nutr..

[218] Hashemi D., Fard M.V., Mohammadhasani K., Barati M., Nattagh-Eshtivani E. (2025). Carotenoids improve obesity and fatty liver disease via gut microbiota: A narrative review. Food Sci. Nutr..

[219] Silva Meneguelli T., Duarte Villas Mishima M., Hermsdorff H.H.M., Martino H.S.D., Bressan J., Tako E. (2024). Effect of carotenoids on gut health and inflammatory status: A systematic review of in vivo animal studies. Crit. Rev. Food Sci. Nutr..

[220] Zhao S., Zhang Y., Ding H., Hu S., Wu X., Ma A., Ma Y. (2023). Lutein prevents liver injury and intestinal barrier dysfunction in rats subjected to chronic alcohol intake. Nutrients.

[221] Magzal F., Turroni S., Fabbrini M., Barone M., Schorr A.V., Ofran A., Tamir S. (2023). A personalized diet intervention improves depression symptoms and changes microbiota and metabolite profiles among community-dwelling older adults. Front. Nutr..

[222] Rocha H.R., Pintado M.E., Gomes A.M., Coelho M.C. (2024). Carotenoids and intestinal harmony: Exploring the link for health. Foods.

[223] Donoso A., González-Durán J., Muñoz A.A., González P.A., Agurto-Muñoz C. (2021). Therapeutic uses of natural astaxanthin: An evidence-based review focused on human clinical trials. Pharmacol. Res..

[224] Lu Q., Li H., Zou Y., Liu H., Yang L. (2021). Astaxanthin as a microalgal metabolite for aquaculture: A review on the synthetic mechanisms, production techniques, and practical application. Algal Res..

[225] Zarneshan S.N., Fakhri S., Farzaei M.H., Khan H., Saso L. (2020). Astaxanthin targets PI3K/Akt signaling pathway toward potential therapeutic applications. Food Chem. Toxicol..

[226] Cunha S.A., Borges S., Baptista-Silva S., Ribeiro T., Oliveira-Silva P., Pintado M., Batista P. (2023). Astaxanthin impact on brain: Health potential and market perspective. Crit. Rev. Food Sci. Nutr..

[227] Patil A.D., Kasabe P.J., Dandge P.B. (2022). Pharmaceutical and nutraceutical potential of natural bioactive pigment: Astaxanthin. Nat. Prod. Bioprospect..

[228] Kuźniar-Pałka A. (2025). The role of oxidative stress in autism spectrum disorder pathophysiology, diagnosis and treatment. Biomedicines.

[229] Kowshik J., Baba A.B., Giri H., Deepak Reddy G., Dixit M., Nagini S. (2014). Astaxanthin inhibits JAK/STAT-3 signaling to abrogate cell proliferation, invasion and angiogenesis in a hamster model of oral cancer. PLoS ONE.

[230] Kim S.H., Kim H. (2019). Astaxanthin modulation of signaling pathways that regulate autophagy. Mar. Drugs.

[231] Kohandel Z., Farkhondeh T., Aschner M., Pourbagher-Shahri A.M., Samarghandian S. (2022). Anti-inflammatory action of astaxanthin and its use in the treatment of various diseases. Biomed. Pharmacother..

[232] Izumi-Nagai K., Nagai N., Ohgami K., Satofuka S., Ozawa Y., Tsubota K., Ohno S., Oike Y., Ishida S. (2008). Inhibition of choroidal neovascularization with an anti-inflammatory carotenoid astaxanthin. Investig. Ophthalmol. Vis. Sci..

[233] Kochi T., Shimizu M., Sumi T., Kubota M., Shirakami Y., Tanaka T., Moriwaki H. (2014). Inhibitory effects of astaxanthin on azoxymethane-induced colonic preneoplastic lesions in C57/BL/KsJ-db/db mice. BMC Gastroenterol..

[234] Chen M.-H., Hong C.-L., Wang Y.-T., Wang T.-J., Chen J.-R. (2022). The effect of astaxanthin treatment on the rat model of fetal alcohol spectrum disorders (FASD). Brain Res. Bull..

[235] Frustaci A., Neri M., Cesario A., Adams J.B., Domenici E., Bernardina B.D., Bonassi S. (2012). Oxidative stress-related biomarkers in autism: Systematic review and meta-analyses. Free Radic. Biol. Med..

[236] Avraham Y., Mankuta D., Lipsker L., Vorobiev L., Patael S., Hassid G., Berry E.M., Albeck A. (2021). Beta-Carotene derivatives as novel therapy for the prevention and treatment of autistic symptoms. Bioorg. Chem..

[237] Al-Amin M.M., Rahman M.M., Khan F.R., Zaman F., Mahmud Reza H. (2015). Astaxanthin improves behavioral disorder and oxidative stress in prenatal valproic acid-induced mice model of autism. Behav. Brain Res..

[238] Xu L., Zhu J., Yin W., Ding X. (2015). Astaxanthin improves cognitive deficits from oxidative stress, nitric oxide synthase and inflammation through upregulation of PI3K/Akt in diabetes rat. Int. J. Clin. Exp. Pathol..

[239] Bahbah E.I., Ghozy S., Attia M.S., Negida A., Bin Emran T., Mitra S., Albadrani G.M., Abdel-Daim M.M., Uddin S., Simal-Gandara J. (2021). Molecular mechanisms of astaxanthin as a potential neurotherapeutic agent. Mar. Drugs.

[240] Chik M.W., Mohd Affandi M.M.R.M., Singh G.K.S. (2022). Detection of Astaxanthin at Different Regions of the Brain in Rats Treated with Astaxanthin Nanoemulsion. J. Pharm. Bioallied Sci..

[241] Fakhri S., Aneva I.Y., Farzaei M.H., Sobarzo-Sánchez E. (2019). The neuroprotective effects of astaxanthin: Therapeutic targets and clinical perspective. Molecules.

[242] Adiguzel E., Bozkurt N.M., Unal G. (2024). Independent and combined effects of astaxanthin and omega-3 on behavioral deficits and molecular changes in a prenatal valproic acid model of autism in rats. Nutr. Neurosci..

[243] Wang J., Shen Y., Li L., Li L., Zhang J., Li M., Qiu F. (2025). Lycopene attenuates D-galactose-induced memory and behavioral deficits by mediating microbiota-SCFAs-gut-brain axis balance in female CD-1 mice. J. Nutr. Biochem..

[244] Yordi E.G., Martínez A.P., Radice M., Scalvenzi L., Abreu-Naranjo R., Uriarte E., Santana L., Matos M.J. (2024). Seaweeds as Source of Bioactive Pigments with Neuroprotective and/or Anti-Neurodegenerative Activities: Astaxanthin and Fucoxanthin. Mar. Drugs.

[245] Gullón B., Gagaoua M., Barba F.J., Gullón P., Zhang W., Lorenzo J.M. (2020). Seaweeds as promising resource of bioactive compounds: Overview of novel extraction strategies and design of tailored meat products. Trends Food Sci. Technol..

[246] Aryee A.N., Agyei D., Akanbi T.O. (2018). Recovery and utilization of seaweed pigments in food processing. Curr. Opin. Food Sci..

[247] Sun G., Xin T., Zhang R., Liu C., Pang Q. (2020). Fucoxanthin attenuates behavior deficits and neuroinflammatory response in 1-methyl-4-phenyl-1,2,3,6 -tetrahydropyridine-induced parkinson’s disease in mice. Pharmacogn. Mag..

[248] Taskiran A.S., Tastemur Y. (2021). The role of nitric oxide in anticonvulsant effects of lycopene supplementation on pentylenetetrazole-induced epileptic seizures in rats. Exp. Brain Res..

[249] Al-Rafiah A.R., Mehdar K.M. (2021). Histopathological and biochemical assessment of neuroprotective effects of sodium valproate and lutein on the pilocarpine albino rat model of epilepsy. Behav. Neurol..

[250] Zhu L., Song Y., Liu H., Wu M., Gong H., Lan H., Zheng X. (2021). Gut microbiota regulation and anti-inflammatory effect of β-carotene in dextran sulfate sodium-stimulated ulcerative colitis in rats. J. Food Sci..

[251] Bromley R.L., Mawer G., Love J., Kelly J., Purdy L., McEwan L., Briggs M., Smith J.C., Sin X., Baker G.A. (2010). Early cognitive development in children born to women with epilepsy: A prospective report. Epilepsia.

[252] Lin L., Tang R., Liu Y., Li Z., Li H., Yang H. (2024). Research on the anti-aging mechanisms of Panax ginseng extract in mice: A gut microbiome and metabolomics approach. Front. Pharmacol..

[253] Panpetch J., Kiatrungrit K., Tuntipopipat S., Tangphatsornruang S., Mhuantong W., Chongviriyaphan N. (2024). Gut Microbiota and Clinical Manifestations in Thai Pediatric Patients with Attention-Deficit Hyperactivity Disorder. J. Pers. Med..

[254] Pruccoli L., Balducci M., Pagliarani B., Tarozzi A. (2024). Antioxidant and neuroprotective effects of fucoxanthin and its metabolite fucoxanthinol: A comparative in vitro study. Curr. Issues Mol. Biol..

[255] Pajot A., Hao Huynh G., Picot L., Marchal L., Nicolau E. (2022). Fucoxanthin from Algae to Human, an Extraordinary Bioresource: Insights and Advances in up and Downstream Processes. Mar. Drugs.

[256] Bigagli E., D’Ambrosio M., Cinci L., Niccolai A., Biondi N., Rodolfi L., Dos Santos Nascimiento L.B., Tredici M.R., Luceri C. (2021). A Comparative In Vitro Evaluation of the Anti-Inflammatory Effects of a Tisochrysis lutea Extract and Fucoxanthin. Mar. Drugs.

[257] Zhao D., Kwon S.-H., Chun Y.S., Gu M.-Y., Yang H.O. (2017). Anti-Neuroinflammatory Effects of Fucoxanthin via Inhibition of Akt/NF-κB and MAPKs/AP-1 Pathways and Activation of PKA/CREB Pathway in Lipopolysaccharide-Activated BV-2 Microglial Cells. Neurochem. Res..

[258] Foiadelli T., Santangelo A., Costagliola G., Costa E., Scacciati M., Riva A., Volpedo G., Smaldone M., Bonuccelli A., Clemente A.M. (2023). Neuroinflammation and status epilepticus: A narrative review unraveling a complex interplay. Front. Pediatr..

[259] Terrone G., Frigerio F., Balosso S., Ravizza T., Vezzani A. (2019). Inflammation and reactive oxygen species in status epilepticus: Biomarkers and implications for therapy. Epilepsy Behav..

[260] Li G., Bauer S., Nowak M., Norwood B., Tackenberg B., Rosenow F., Knake S., Oertel W.H., Hamer H.M. (2011). Cytokines and epilepsy. Seizure.

[261] Kandy S.K., Nimonkar M.M., Dash S.S., Mehta B., Markandeya Y.S. (2022). Astaxanthin Protection against Neuronal Excitotoxicity via Glutamate Receptor Inhibition and Improvement of Mitochondrial Function. Mar. Drugs.

[262] Lu Y., Xie T., He X.-X., Mao Z.-F., Jia L.-J., Wang W.-P., Zhen J.-L., Liu L.-M. (2015). Astaxanthin rescues neuron loss and attenuates oxidative stress induced by amygdala kindling in adult rat hippocampus. Neurosci. Lett..

[263] Ata Yaseen Abdulqader Y., Abdel Kawy H.S., Mohammed Alkreathy H., Abdullah Rajeh N. (2021). The potential antiepileptic activity of astaxanthin in epileptic rats treated with valproic acid. Saudi Pharm. J..

[264] Wang M., Deng X., Xie Y., Chen Y. (2020). Astaxanthin Attenuates Neuroinflammation in Status Epilepticus Rats by Regulating the ATP-P2X7R Signal. Drug Des. Dev. Ther..

[265] Deng X., Wang M., Hu S., Feng Y., Shao Y., Xie Y., Wu M., Chen Y., Shi X. (2019). The Neuroprotective Effect of Astaxanthin on Pilocarpine-Induced Status Epilepticus in Rats. Front. Cell. Neurosci..

[266] Lu Y., Wang X., Feng J., Xie T., Si P., Wang W. (2019). Neuroprotective effect of astaxanthin on newborn rats exposed to prenatal maternal seizures. Brain Res. Bull..

[267] Baccouch R., Shi Y., Vernay E., Mathelié-Guinlet M., Taib-Maamar N., Villette S., Feuillie C., Rascol E., Nuss P., Lecomte S. (2022). The impact of lipid polyunsaturation on the physical and mechanical properties of lipid membranes. Biochim. ET Biophys. Acta (BBA) -Biomembr..

[268] Djuricic I., Calder P.C. (2021). Beneficial Outcomes of Omega-6 and Omega-3 Polyunsaturated Fatty Acids on Human Health: An Update for 2021. Nutrients.

[269] Chen W., Li T., Du S., Chen H., Wang Q. (2023). Microalgal polyunsaturated fatty acids: Hotspots and production techniques. Front. Bioeng. Biotechnol..

[270] Mariam I., Bettiga M., Rova U., Christakopoulos P., Matsakas L., Patel A. (2024). Ameliorating microalgal OMEGA production using omics platforms. Trends Plant Sci..

[271] Zhao W., Zhu J., Yang S., Liu J., Sun Z., Sun H. (2024). Microalgal metabolic engineering facilitates precision nutrition and dietary regulation. Sci. Total Environ..

[272] Kumari A., Pabbi S., Tyagi A. (2024). Recent advances in enhancing the production of long chain omega-3 fatty acids in microalgae. Crit. Rev. Food Sci. Nutr..

[273] Chitranjali T., Anoop Chandran P., Muraleedhara Kurup G. (2015). Omega-3 fatty acid concentrate from Dunaliella salina possesses anti-inflammatory properties including blockade of NF-κB nuclear translocation. Immunopharmacol. Immunotoxicol..

[274] Robertson R.C., Guihéneuf F., Bahar B., Schmid M., Stengel D.B., Fitzgerald G.F., Ross R., Stanton C. (2015). The Anti-Inflammatory Effect of Algae-Derived Lipid Extracts on Lipopolysaccharide (LPS)-Stimulated Human THP-1 Macrophages. Mar. Drugs.

[275] Borowitzka M.A., Borowitzka M.A., Beardall J., Raven J.A. (2016). Algal Physiology and Large-Scale Outdoor Cultures of Microalgae. The Physiology of Microalgae.

[276] Piyatilleke S., Thevarajah B., Nimarshana P.H.V., Ariyadasa T.U. (2024). Large-scale production of Nannochloropsis-derived EPA: Current status and perspectives via a biorefinery context. Food Bioprod. Process..

[277] DiNicolantonio J.J., O’Keefe J.H. (2020). The Importance of Marine Omega-3s for Brain Development and the Prevention and Treatment of Behavior, Mood, and Other Brain Disorders. Nutrients.

[278] Chaaba R., Bouaziz A., Ben Amor A., Mnif W., Hammami M., Mehri S. (2023). Fatty acid profile and genetic variants of proteins involved in fatty acid metabolism could be considered as disease predictor. Diagnostics.

[279] Banskota A.H., Stefanova R., Sperker S., Lall S., Craigie J.S., Hafting J.T. (2014). Lipids isolated from the cultivated red alga Chondrus crispus inhibit nitric oxide production. J. Appl. Phycol..

[280] Khamlaoui W., Mehri S., Hammami S., Hammouda S., Chraeif I., Elosua R., Hammami M. (2020). Association Between Genetic Variants in FADS1-FADS2 and ELOVL2 and Obesity, Lipid Traits, and Fatty Acids in Tunisian Population. Clin. Appl. Thromb. Hemost..

[281] Yamagishi K., Ikeda A., Chei C.-L., Noda H., Umesawa M., Cui R., Muraki I., Ohira T., Imano H., Sankai T. (2017). Serum α-linolenic and other ω-3 fatty acids, and risk of disabling dementia: Community-based nested case-control study. Clin. Nutr..

[282] Sun C., Zou M., Wang X., Xia W., Ma Y., Liang S., Hao Y., Wu L., Fu S. (2018). FADS1-FADS2 and ELOVL2 gene polymorphisms in susceptibility to autism spectrum disorders in Chinese children. BMC Psychiatry.

[283] Iyer S.H., Yeh M.Y., Netzel L., Lindsey M.G., Wallace M., Simeone K.A., Simeone T.A. (2024). Dietary and Metabolic Approaches for Treating Autism Spectrum Disorders, Affective Disorders and Cognitive Impairment Comorbid with Epilepsy: A Review of Clinical and Preclinical Evidence. Nutrients.

[284] Omrani S., Taheri M., Omrani M.D., Arsang-Jang S., Ghafouri-Fard S. (2019). The effect of omega-3 fatty acids on clinical and paraclinical features of intractable epileptic patients: A triple blind randomized clinical trial. Clin. Transl. Med..

[285] Yao J., Lu Y., Zhi M., Hu P., Wu W., Gao X. (2017). Dietary n-3 polyunsaturated fatty acids ameliorate Crohn’s disease in rats by modulating the expression of PPAR-γ/NFAT. Mol. Med. Rep..

[286] Di Miceli M., Martinat M., Rossitto M., Aubert A., Alashmali S., Bosch-Bouju C., Fioramonti X., Joffre C., Bazinet R.P., Layé S. (2022). Dietary Long-Chain n-3 Polyunsaturated Fatty Acid Supplementation Alters Electrophysiological Properties in the Nucleus Accumbens and Emotional Behavior in Naïve and Chronically Stressed Mice. Int. J. Mol. Sci..

[287] Reichenberg A., Yirmiya R., Schuld A., Kraus T., Haack M., Morag A., Pollmächer T. (2001). Cytokine-associated emotional and cognitive disturbances in humans. Arch. Gen. Psychiatry.

[288] Yui K., Imataka G. (2025). A Comparison of the Treatment Effects of a Risperidone Solution, an Equal Ratio of DHA/ARA, and a Larger Ratio of Omega-6 PUFA Added to Omega-3 PUFA: An Open-Label Clinical Trial. Curr. Issues Mol. Biol..

[289] Horvath A., Łukasik J., Szajewska H. (2017). ω-3 Fatty Acid Supplementation Does Not Affect Autism Spectrum Disorder in Children: A Systematic Review and Meta-Analysis. J. Nutr..

[290] Jiang Y., Dang W., Nie H., Kong X., Jiang Z., Guo J. (2023). Omega-3 polyunsaturated fatty acids and/or vitamin D in autism spectrum disorders: A systematic review. Front. Psychiatry.

[291] Mazahery H., Conlon C.A., Beck K.L., Mugridge O., Kruger M.C., Stonehouse W., Camargo C.A., Meyer B.J., Jones B., von Hurst P.R. (2019). A randomised controlled trial of vitamin D and omega-3 long chain polyunsaturated fatty acids in the treatment of irritability and hyperactivity among children with autism spectrum disorder. J. Steroid Biochem. Mol. Biol..

[292] Bozzatello P., Novelli R., Montemagni C., Rocca P., Bellino S. (2024). Nutraceuticals in psychiatric disorders: A systematic review. Int. J. Mol. Sci..

[293] Woods S., Doherty A., Hill J. (2024). Dietary interventions in autism: A critical appraisal and commentary on the findings of a systematic review. Br. J. Neurosci. Nurs..

[294] Johnson C.R., Handen B.L., Zimmer M., Sacco K. (2010). Polyunsaturated Fatty Acid Supplementation in Young Children with Autism. J. Dev. Phys. Disabil..

[295] Doherty M., Foley K.-R., Schloss J. (2025). Complementary and Alternative Medicine for Autism—A Systematic Review. J. Autism Dev. Disord..

[296] Abbasi H., Parhiz A., Khoshdooz S., Bakhshimoghaddam F., Doaei S., Gholamalizadeh M. (2025). Impact of omega-3 fatty acid supplementation on clinical manifestations in autism spectrum disorders: An umbrella review of meta-analyses. Nutr. Res. Rev..

[297] Boone K.M., Klebanoff M.A., Rogers L.K., Rausch J., Coury D.L., Keim S.A. (2022). Effects of Omega-3-6-9 fatty acid supplementation on behavior and sleep in preterm toddlers with autism symptomatology: Secondary analysis of a randomized clinical trial. Early Hum. Dev..

[298] Keim S.A., Gracious B., Boone K.M., Klebanoff M.A., Rogers L.K., Rausch J., Coury D.L., Sheppard K.W., Husk J., Rhoda D. (2018). ω-3 and ω-6 Fatty Acid Supplementation May Reduce Autism Symptoms Based on Parent Report in Preterm Toddlers. J. Nutr..

[299] Boone K.M., Parrott A., Rausch J., Yeates K.O., Klebanoff M.A., Turner A.N., Keim S.A. (2020). Fatty acid supplementation and socioemotional outcomes: Secondary analysis of a randomized trial. Pediatrics.

[300] Al-Dbass A., Ben Bacha A., Moubayed N.M., Bhat R.S., Al-Mutairi M., Alnakhli O.M., Al-Mrshoud M., Alfawaz H., Daghestani M., El-Ansary A. (2021). Comparative Studies on Phospholipase A2 as a Marker for Gut Microbiota- liver-brain Axis in a rodent Model of Autism. Curr. Proteom..

[301] Lyall K., Westlake M., Musci R.J., Gachigi K., Barrett E.S., Bastain T.M., Bush N.R., Buss C., Camargo C.A., Croen L.A. (2024). Association of maternal fish consumption and ω-3 supplement use during pregnancy with child autism-related outcomes: Results from a cohort consortium analysis. Am. J. Clin. Nutr..

[302] Samà M., Musillo C., Cirulli F. (2025). Counteracting the effects of maternal obesity on offspring neurodevelopment through Omega-3-based nutritional strategies. Neuroscience.

[303] Wootton R.E., Dack K., Jones H.J., Riglin L., Madley-Dowd P., Borges C., Pagoni P., Roth C., Brantsæter A.L., Corfield E.C. (2024). Testing maternal effects of vitamin-D and omega-3 levels on offspring neurodevelopmental traits in the Norwegian Mother, Father and Child Cohort Study. Psychol. Med..

[304] Almohmadi N.H. (2024). Brain-gut-brain axis, nutrition, and autism spectrum disorders: A review. Transl. Pediatr..

[305] Mazahery H., Stonehouse W., Delshad M., Kruger M.C., Conlon C.A., Beck K.L., Von Hurst P.R. (2017). Relationship between Long Chain n-3 Polyunsaturated Fatty Acids and Autism Spectrum Disorder: Systematic Review and Meta-Analysis of Case-Control and Randomised Controlled Trials. Nutrients.

[306] Turpin V., Schaffhauser M., Thabault M., Aubert A., Joffre C., Balado E., Longueville J.-E., Francheteau M., Burucoa C., Pichon M. (2023). Mice prenatally exposed to valproic acid do not show autism-related disorders when fed with polyunsaturated fatty acid-enriched diets. Sci. Rep..

[307] Roy-Chowdhury S., Jang S., Abderemane-Ali F., Naughton F., Grabe M., Minor D.L. (2025). Structure of the human K2P13.1 channel reveals a hydrophilic pore restriction and lipid cofactor site. Nat. Struct. Mol. Biol..

[308] Jayapala H.P.S., Lim S.Y. (2023). N-3 Polyunsaturated Fatty Acids and Gut Microbiota. Comb. Chem. High. Throughput Screen..

[309] Kumar V., Rohilla A., Ahire J.J. (2025). Omega-3 fatty acids and the gut microbiome: A new frontier in cardiovascular disease prevention. Discov. Med..

[310] Fock E., Parnova R. (2023). Mechanisms of Blood-Brain Barrier Protection by Microbiota-Derived Short-Chain Fatty Acids. Cells.

[311] Zinkow A., Grodzicki W., Czerwińska M., Dziendzikowska K. (2024). Molecular Mechanisms Linking Omega-3 Fatty Acids and the Gut-Brain Axis. Molecules.

[312] Fu Y., Wang Y., Gao H., Li D., Jiang R., Ge L., Tong C., Xu K. (2021). Associations among Dietary Omega-3 Polyunsaturated Fatty Acids, the Gut Microbiota, and Intestinal Immunity. Mediat. Inflamm..

[313] Bauer K.C., York E.M., Cirstea M.S., Radisavljevic N., Petersen C., Huus K.E., Brown E.M., Bozorgmehr T., Berdún R., Bernier L. (2022). Gut microbes shape microglia and cognitive function during malnutrition. Glia.

[314] Reemst K., Tims S., Yam K.-Y., Mischke M., Knol J., Brul S., Schipper L., Korosi A. (2022). The Role of the Gut Microbiota in the Effects of Early-Life Stress and Dietary Fatty Acids on Later-Life Central and Metabolic Outcomes in Mice. mSystems.

[315] Wu S.-X., Li J., Zhou D.-D., Xiong R.-G., Huang S.-Y., Saimaiti A., Shang A., Li H.-B. (2022). Possible effects and mechanisms of dietary natural products and nutrients on depression and anxiety: A narrative review. Antioxidants.

[316] Moreno C., de la Cruz A., Oliveras A., Kharche S.R., Guizy M., Comes N., Starý T., Ronchi C., Rocchetti M., Baró I. (2015). Marine n-3 PUFAs modulate IKs gating, channel expression, and location in membrane microdomains. Cardiovasc. Res..

[317] Larsson J.E., Frampton D.J.A., Liin S.I. (2020). Polyunsaturated fatty acids as modulators of KV7 channels. Front. Physiol..

[318] Barrese V., Stott J.B., Greenwood I.A. (2018). KCNQ-Encoded Potassium Channels as Therapeutic Targets. Annu. Rev. Pharmacol. Toxicol..

[319] Valenzuela C. (2016). M-channels and n-3 polyunsaturated fatty acids: Role in pain and epilepsy. Acta Physiol..

[320] Simeone T.A., Matthews S.A., Samson K.K., Simeone K.A. (2017). Regulation of brain PPARgamma2 contributes to ketogenic diet anti-seizure efficacy. Exp. Neurol..

[321] Zhao Y.-C., Wang C.-C., Li X.-Y., Wang D.-D., Wang Y.-M., Xue C.-H., Wen M., Zhang T.-T. (2023). Supplementation of n-3 PUFAs in Adulthood Attenuated Susceptibility to Pentylenetetrazol Induced Epilepsy in Mice Fed with n-3 PUFAs Deficient Diet in Early Life. Mar. Drugs.

[322] Elsadek A.E., Maksoud Y.H.A., Suliman H.A., Al-Shokary A.H., Ibrahim A.O., Kamal N.M., Fathallah M.G.E.D., Elshorbagy H.H., Abdelghani W.E. (2021). Omega-3 supplementation in children with ADHD and intractable epilepsy. J. Clin. Neurosci..

[323] Sohouli M.H., Razmpoosh E., Zarrati M., Jaberzadeh S. (2022). The effect of omega-3 fatty acid supplementation on seizure frequency in individuals with epilepsy: A systematic review and meta-analysis. Nutr. Neurosci..

[324] Rheims S., Herbillon V., Gaillard S., Mercier C., Villeuve N., Villéga F., Cances C., Castelnau P., Napuri S., de Saint-Martin A. (2024). Phosphatidylserine enriched with polyunsaturated n-3 fatty acid supplementation for attention-deficit hyperactivity disorder in children and adolescents with epilepsy: A randomized placebo-controlled trial. Epilepsia Open.

[325] Vogel D.D., Ortiz-Villatoro N.N., de Freitas L., Aimbire F., Scorza F.A., Albertini R., Scorza C.A. (2021). Repetitive transcranial photobiomodulation but not long-term omega-3 intake reduces epileptiform discharges in rats with stroke-induced epilepsy. J. Biophotonics.

[326] Yonezawa T., Marasigan C.N.B.B., Matsumiya Y., Maeda S., Motegi T., Momoi Y. (2023). Effects of high-dose docosahexaenoic acid supplementation as an add-on therapy for canine idiopathic epilepsy: A pilot study. Open Vet. J..

[327] Hemida M., Rosendahl S., Jokinen T.S., Moore R., Vuori K.A., Anturaniemi J., Hielm-Björkman A. (2023). Assessing the association between supplemented puppyhood dietary fat sources and owner-reported epilepsy in adulthood, among Finnish companion dogs. Front. Vet. Sci..

[328] Ray S., Nathan J., Godhia M. (2024). Efficacy and tolerability of classical and polyunsaturated fatty acids ketogenic diet in controlling paediatric refractory epilepsy—A randomized study. Epilepsy Res..

[329] Ghafouri-Fard S., Hashemi M., Rafigh M., Omrani M.A., Hussen B.M., Sayad A., Taheri M. (2021). Altered IFN-γ Levels after Treatment of Epileptic Patients with Omega-3 Fatty Acids. J. Mol. Neurosci..

[330] Alhattab M., Moorthy L.S., Patel D., Franco C.M.M., Puri M. (2024). Oleaginous microbial lipids’ potential in the prevention and treatment of neurological disorders. Mar. Drugs.

[331] Liang Z., Lou Y., Li Z., Liu S. (2023). Causal relationship between human blood omega-3 fatty acids and the risk of epilepsy: A two-sample Mendelian randomization study. Front. Neurol..

[332] Asadi-Pooya A.A., Simani L. (2021). Clinical trials of vitamin-mineral supplementations in people with epilepsy: A systematic review. Clin. Nutr..

[333] Eaton C., Yong K., Walter V., Mbizvo G.K., Rhodes S., Chin R.F. (2022). Stimulant and non-stimulant drug therapy for people with attention deficit hyperactivity disorder and epilepsy. Cochrane Database Syst. Rev..

[334] Pidoplichko V.I., Figueiredo T.H., Braga M.F., Pan H., Marini A.M. (2023). Alpha-linolenic acid enhances the facilitation of GABAergic neurotransmission in the BLA and CA1. Exp. Biol. Med..

[335] Meeusen H., Kalf R.S., Broekaart D.W., Silva J.P., Verkuyl J.M., van Helvoort A., Gorter J.A., van Vliet E.A., Aronica E. (2024). Effective reduction in seizure severity and prevention of a fatty liver by a novel low ratio ketogenic diet composition in the rapid kindling rat model of epileptogenesis. Exp. Neurol..

[336] Sadat-Hossieny Z., Robalino C.P., Pennell P.B., Cohen M.J., Loring D.W., May R.C., Block T., Swiatlo T., Meador K.J. (2021). Folate fortification of food: Insufficient for women with epilepsy. Epilepsy Behav..

[337] Hua Y., Shen J., Fan R., Xiao R., Ma W. (2022). High-fat diets containing different types of fatty acids modulate gut-brain axis in obese mice. Nutr. Metab..

[338] Fan R., Hua Y., Shen J., Xiao R., Ma W. (2022). Dietary fatty acids affect learning and memory ability via regulating inflammatory factors in obese mice. J. Nutr. Biochem..

[339] Techaniyom P., Korsirikoon C., Rungruang T., Pakaprot N., Prombutara P., Mukda S., Kettawan A.K., Kettawan A. (2024). Cold-pressed perilla seed oil: Investigating its protective influence on the gut-brain axis in mice with rotenone-induced Parkinson’s disease. Food Sci. Nutr..

[340] Huang H., Zhao T., Ma W. (2025). Omega-3 polyunsaturated fatty acids attenuates cognitive impairment via the gut-brain axis in diabetes-associated cognitive dysfunction rats. Brain Behav. Immun..

[341] Horn J., Mayer D.E., Chen S., Mayer E.A. (2022). Role of diet and its effects on the gut microbiome in the pathophysiology of mental disorders. Transl. Psychiatry.

[342] Shi R., Gao D., Stoika R., Liu K., Sik A., Jin M. (2024). Potential implications of polyphenolic compounds in neurodegenerative diseases. Crit. Rev. Food Sci. Nutr..

[343] Madireddy S., Madireddy S. (2023). Therapeutic strategies to ameliorate neuronal damage in epilepsy by regulating oxidative stress, mitochondrial dysfunction, and neuroinflammation. Brain Sci..

[344] Serra D., Almeida L.M., Dinis T.C.P. (2019). Polyphenols as food bioactive compounds in the context of Autism Spectrum Disorders: A critical mini-review. Neurosci. Biobehav. Rev..

[345] Serra D., Almeida L.M., Dinis T.C.P. (2020). Polyphenols in the management of brain disorders: Modulation of the microbiota-gut-brain axis. Adv. Food Nutr. Res..

[346] Grabarczyk M., Justyńska W., Czpakowska J., Smolińska E., Bielenin A., Glabinski A., Szpakowski P. (2024). Role of plant phytochemicals: Resveratrol, curcumin, luteolin and quercetin in demyelination, neurodegeneration, and epilepsy. Antioxidants.

[347] Thapliyal S., Singh T., Handu S., Bisht M., Kumari P., Arya P., Srivastava P., Gandham R. (2021). A review on potential footprints of ferulic acid for treatment of neurological disorders. Neurochem. Res..

[348] Wu X., Zhou Y., Xi Y., Zhou H., Tang Z., Xiong L., Qin D. (2024). Polyphenols: Natural Food-Grade Biomolecules for the Treatment of Nervous System Diseases from a Multi-Target Perspective. Pharmaceuticals.

[349] Pereira L., Cotas J. (2023). Therapeutic potential of polyphenols and other micronutrients of marine origin. Mar. Drugs.

[350] Ghallab D.S., Ibrahim R.S., Mohyeldin M.M., Shawky E. (2024). Marine algae: A treasure trove of bioactive anti-inflammatory compounds. Mar. Pollut. Bull..

[351] Ginwala R., Bhavsar R., Chigbu D.I., Jain P., Khan Z.K. (2019). Potential Role of Flavonoids in Treating Chronic Inflammatory Diseases with a Special Focus on the Anti-Inflammatory Activity of Apigenin. Antioxidants.

[352] Ferdous U.T., Balia Yusof Z.N. (2021). Insight into Potential Anticancer Activity of Algal Flavonoids: Current Status and Challenges. Molecules.

[353] Guil-Guerrero J.L., Prates J.A.M. (2025). Microalgae Bioactives for Functional Food Innovation and Health Promotion. Foods.

[354] Rajan D.K., Mohan K., Zhang S., Ganesan A.R. (2021). Dieckol: A brown algal phlorotannin with biological potential. Biomed. Pharmacother..

[355] Um M.Y., Lim D.W., Son H.J., Cho S., Lee C. (2018). Phlorotannin-rich fraction from Ishige foliacea brown seaweed prevents the scopolamine-induced memory impairment via regulation of ERK-CREB-BDNF pathway. J. Funct. Foods.

[356] Kwon S., Yoon M., Lee J., Moon K.-D., Kim D., Kim S.-B., Cho S. (2019). A Standardized Phlorotannin Supplement Attenuates Caffeine-Induced Sleep Disruption in Mice. Nutrients.

[357] Yammine A., Zarrouk A., Nury T., Vejux A., Latruffe N., Vervandier-Fasseur D., Samadi M., Mackrill J.J., Greige-Gerges H., Auezova L. (2020). Prevention by Dietary Polyphenols (Resveratrol, Quercetin, Apigenin) Against 7-Ketocholesterol-Induced Oxiapoptophagy in Neuronal N2a Cells: Potential Interest for the Treatment of Neurodegenerative and Age-Related Diseases. Cells.

[358] Lin K., Zhou M., Leng C., Tao X., Zhou R., Li Y., Sun B., Shu X., Liu W. (2022). Neuroprotective Effect of Polyphenol Extracts from Terminalia chebula Retz. against Cerebral Ischemia-Reperfusion Injury. Molecules.

[359] Truong V.-L., Jun M., Jeong W.-S. (2018). Role of resveratrol in regulation of cellular defense systems against oxidative stress. Biofactors.

[360] Grosso C., Santos M., Barroso M.F. (2023). From Plants to Psycho-Neurology: Unravelling the Therapeutic Benefits of Bioactive Compounds in Brain Disorders. Antioxidants.

[361] Nasiry D., Khalatbary A.R. (2024). Natural polyphenols for the management of autism spectrum disorder: A review of efficacy and molecular mechanisms. Nutr. Neurosci..

[362] Socała K., Żmudzka E., Lustyk K., Zagaja M., Brighenti V., Costa A.M., Andres-Mach M., Pytka K., Martinelli I., Mandrioli J. (2024). Therapeutic potential of stilbenes in neuropsychiatric and neurological disorders: A comprehensive review of preclinical and clinical evidence. Phytother. Res..

[363] Shu X.-H., Wang L.-L., Li H., Song X., Shi S., Gu J.-Y., Wu M.-L., Chen X.-Y., Kong Q.-Y., Liu J. (2015). Diffusion efficiency and bioavailability of resveratrol administered to rat brain by different routes: Therapeutic implications. Neurotherapeutics.

[364] de Oliveira M.R., Chenet A.L., Duarte A.R., Scaini G., Quevedo J. (2018). Molecular Mechanisms Underlying the Anti-depressant Effects of Resveratrol: A Review. Mol. Neurobiol..

[365] Zhang L.-X., Li C.-X., Kakar M.U., Khan M.S., Wu P.-F., Amir R.M., Dai D.-F., Naveed M., Li Q.-Y., Saeed M. (2021). Resveratrol (RV): A pharmacological review and call for further research. Biomed. Pharmacother..

[366] Mahdipour R., Ebrahimzadeh-Bideskan A., Hosseini M., Shahba S., Lombardi G., Malvandi A.M., Mohammadipour A. (2023). The benefits of grape seed extract in neurological disorders and brain aging. Nutr. Neurosci..

[367] Mantena S.K., Katiyar S.K. (2006). Grape seed proanthocyanidins inhibit UV-radiation-induced oxidative stress and activation of MAPK and NF-kappaB signaling in human epidermal keratinocytes. Free Radic. Biol. Med..

[368] Tikhonova M.A., Tikhonova N.G., Tenditnik M.V., Ovsyukova M.V., Akopyan A.A., Dubrovina N.I., Amstislavskaya T.G., Khlestkina E.K. (2020). Effects of Grape Polyphenols on the Life Span and Neuroinflammatory Alterations Related to Neurodegenerative Parkinson Disease-Like Disturbances in Mice. Molecules.

[369] Figueira I., Tavares L., Jardim C., Costa I., Terrasso A.P., Almeida A.F., Govers C., Mes J.J., Gardner R., Becker J.D. (2019). Blood-brain barrier transport and neuroprotective potential of blackberry-digested polyphenols: An in vitro study. Eur. J. Nutr..

[370] Chandel P., Thapa K., Kanojia N., Rani L., Singh T.G., Rohilla P. (2024). Exploring therapeutic potential of phytoconstituents as a gut microbiota modulator in the management of neurological and psychological disorders. Neuroscience.

[371] Taliou A., Zintzaras E., Lykouras L., Francis K. (2013). An open-label pilot study of a formulation containing the anti-inflammatory flavonoid luteolin and its effects on behavior in children with autism spectrum disorders. Clin. Ther..

[372] Shanmugam H., Ganguly S., Priya B. (2022). Plant food bioactives and its effects on gut microbiota profile modulation for better brain health and functioning in Autism Spectrum Disorder individuals: A review. Food Front..

[373] Tang S., Hu Y., Luo J., Hu M., Chen M., Ye D., Ye J., Xue F. (2024). Insight into the Gut-Brain Axis and the Productive Performance and Egg Quality Response to Kudzu Leaf Flavonoid Supplementation in Late-Laying Hens. Animals.

[374] Amadi C.N., Orish C.N., Frazzoli C., Orisakwe O.E. (2022). Dietary interventions for autism spectrum disorder: An updated systematic review of human studies. Psychiatriki.

[375] Cruz-Martins N., Quispe C., Kırkın C., Şenol E., Zuluğ A., Özçelik B., Ademiluyi A.O., Oyeniran O.H., Semwal P., Kumar M. (2021). Paving Plant-Food-Derived Bioactives as Effective Therapeutic Agents in Autism Spectrum Disorder. Oxid. Med. Cell. Longev..

[376] de la Rubia Ortí J.E., Moneti C., Serrano-Ballesteros P., Castellano G., Bayona-Babiloni R., Carriquí-Suárez A.B., Motos-Muñoz M., Proaño B., Benlloch M. (2023). Liposomal Epigallocatechin-3-Gallate for the Treatment of Intestinal Dysbiosis in Children with Autism Spectrum Disorder: A Comprehensive Review. Nutrients.

[377] Liu Z., de Bruijn W.J.C., Bruins M.E., Vincken J.-P. (2020). Reciprocal Interactions between Epigallocatechin-3-gallate (EGCG) and Human Gut Microbiota In Vitro. J. Agric. Food Chem..

[378] Ushiroda C., Naito Y., Takagi T., Uchiyama K., Mizushima K., Higashimura Y., Yasukawa Z., Okubo T., Inoue R., Honda A. (2019). Green tea polyphenol (epigallocatechin-3-gallate) improves gut dysbiosis and serum bile acids dysregulation in high-fat diet-fed mice. J. Clin. Biochem. Nutr..

[379] Wu Z., Huang S., Li T., Li N., Han D., Zhang B., Xu Z.Z., Zhang S., Pang J., Wang S. (2021). Gut microbiota from green tea polyphenol-dosed mice improves intestinal epithelial homeostasis and ameliorates experimental colitis. Microbiome.

[380] Mamun A.A., Shao C., Geng P., Wang S., Xiao J. (2024). Polyphenols Targeting NF-κB Pathway in Neurological Disorders: What We Know So Far?. Int. J. Biol. Sci..

[381] Hamsalakshmi Alex A.M., Arehally Marappa M., Joghee S., Chidambaram S.B. (2022). Therapeutic benefits of flavonoids against neuroinflammation: A systematic review. Inflammopharmacology.

[382] Huang T.-C., Lu K.-T., Wo Y.-Y.P., Wu Y.-J., Yang Y.-L. (2011). Resveratrol protects rats from Aβ-induced neurotoxicity by the reduction of iNOS expression and lipid peroxidation. PLoS ONE.

[383] Solberg N.O., Chamberlin R., Vigil J.R., Deck L.M., Heidrich J.E., Brown D.C., Brady C.I., Jagt T.A.V., Garwood M., Bisoffi M. (2014). Optical and SPION-enhanced MR imaging shows that trans-stilbene inhibitors of NF-κB concomitantly lower Alzheimer’s disease plaque formation and microglial activation in AβPP/PS-1 transgenic mouse brain. J. Alzheimers Dis..

[384] Savino R., Medoro A., Ali S., Scapagnini G., Maes M., Davinelli S. (2023). The emerging role of flavonoids in autism spectrum disorder: A systematic review. J. Clin. Med..

[385] Tassinari M., Mottolese N., Galvani G., Ferrara D., Gennaccaro L., Loi M., Medici G., Candini G., Rimondini R., Ciani E. (2022). Luteolin treatment ameliorates brain development and behavioral performance in a mouse model of CDKL5 deficiency disorder. Int. J. Mol. Sci..

[386] Parker-Athill E., Luo D., Bailey A., Giunta B., Tian J., Shytle R.D., Murphy T., Legradi G., Tan J. (2009). Flavonoids, a prenatal prophylaxis via targeting JAK2/STAT3 signaling to oppose IL-6/MIA associated autism. J. Neuroimmunol..

[387] Bertolino B., Crupi R., Impellizzeri D., Bruschetta G., Cordaro M., Siracusa R., Esposito E., Cuzzocrea S. (2017). Beneficial Effects of Co-Ultramicronized Palmitoylethanolamide/Luteolin in a Mouse Model of Autism and in a Case Report of Autism. CNS Neurosci. Ther..

[388] Jayaprakash P., Isaev D., Yang K.-H.S., Beiram R., Oz M., Sadek B. (2024). Apigenin Alleviates Autistic-like Stereotyped Repetitive Behaviors and Mitigates Brain Oxidative Stress in Mice. Pharmaceuticals.

[389] Liu Z.S.J., Truong T.T.T., Bortolasci C.C., Spolding B., Panizzutti B., Swinton C., Kim J.H., Hernández D., Kidnapillai S., Gray L. (2024). The potential of baicalin to enhance neuroprotection and mitochondrial function in a human neuronal cell model. Mol. Psychiatry.

[390] Kato Y., Shirai R., Ohbuchi K., Oizumi H., Yamamoto M., Miyata W., Iguchi T., Mimaki Y., Miyamoto Y., Yamauchi J. (2023). Hesperetin Ameliorates Inhibition of Neuronal and Oligodendroglial Cell Differentiation Phenotypes Induced by Knockdown of Rab2b, an Autism Spectrum Disorder-Associated Gene Product. Neurol. Int..

[391] Li Z., You Z., Li M., Pang L., Cheng J., Wang L. (2017). Protective effect of resveratrol on the brain in a rat model of epilepsy. Neurosci. Bull..

[392] Siddiqui M.A., Asad M., Akhter J., Hoda U., Rastogi S., Arora I., Aggarwal N.B., Samim M. (2022). Resveratrol-Loaded Glutathione-Coated Collagen Nanoparticles Attenuate Acute Seizures by Inhibiting HMGB1 and TLR-4 in the Hippocampus of Mice. ACS Chem. Neurosci..

[393] Lofrumento D.D., Nicolardi G., Cianciulli A., De Nuccio F., La Pesa V., Carofiglio V., Dragone T., Calvello R., Panaro M.A. (2014). Neuroprotective effects of resveratrol in an MPTP mouse model of Parkinson’s-like disease: Possible role of SOCS-1 in reducing pro-inflammatory responses. Innate Immun..

[394] Mishra V., Shuai B., Kodali M., Shetty G.A., Hattiangady B., Rao X., Shetty A.K. (2015). Resveratrol Treatment after Status Epilepticus Restrains Neurodegeneration and Abnormal Neurogenesis with Suppression of Oxidative Stress and Inflammation. Sci. Rep..

[395] Wu Z., Xu Q., Zhang L., Kong D., Ma R., Wang L. (2009). Protective effect of resveratrol against kainate-induced temporal lobe epilepsy in rats. Neurochem. Res..

[396] Zamora-Bello I., Rivadeneyra-Domínguez E., Rodríguez-Landa J.F. (2022). Anticonvulsant Effect of Turmeric and Resveratrol in Lithium/Pilocarpine-Induced Status Epilepticus in Wistar Rats. Molecules.

[397] Teka T., Zhang L., Ge X., Li Y., Han L., Yan X. (2022). Stilbenes: Source plants, chemistry, biosynthesis, pharmacology, application and problems related to their clinical Application-A comprehensive review. Phytochemistry.

[398] Siddiqui M.A., Akhter J., Bashir D.J., Manzoor S., Rastogi S., Arora I., Aggarwal N.B., Samim M. (2021). Resveratrol loaded nanoparticles attenuate cognitive impairment and inflammatory markers in PTZ-induced kindled mice. Int. Immunopharmacol..

[399] Kaur H., Bal A., Sandhir R. (2014). Curcumin supplementation improves mitochondrial and behavioral deficits in experimental model of chronic epilepsy. Pharmacol. Biochem. Behav..

[400] Pedroso J., Schneider S.E., Lima-Rezende C.A., Aguiar G.P.S., Müller L.G., Oliveira J.V., Piato A., Siebel A.M. (2022). Evaluation of Resveratrol and Piceatannol Anticonvulsant Potential in Adult Zebrafish (*Danio rerio*). Neurochem. Res..

[401] Decui L., Garbinato C.L.L., Schneider S.E., Mazon S.C., Almeida E.R., Aguiar G.P.S., Müller L.G., Oliveira J.V., Siebel A.M. (2020). Micronized resveratrol shows promising effects in a seizure model in zebrafish and signalizes an important advance in epilepsy treatment. Epilepsy Res..

[402] Almeida E.R., Lima-Rezende C.A., Schneider S.E., Garbinato C., Pedroso J., Decui L., Aguiar G.P.S., Müller L.G., Oliveira J.V., Siebel A.M. (2021). Micronized Resveratrol Shows Anticonvulsant Properties in Pentylenetetrazole-Induced Seizure Model in Adult Zebrafish. Neurochem. Res..

[403] Erfani M., Ashrafzadeh F., Rahimi H.R., Ebrahimi S.A., Kalali K., Beiraghi Toosi M., Faraji Rad E. (2022). Effect of curcumin on pediatric intractable epilepsy. Iran. J. Child Neurol..

[404] Huang R., Zhu Y., Lin L., Song S., Cheng L., Zhu R. (2020). Solid Lipid Nanoparticles Enhanced the Neuroprotective Role of Curcumin against Epilepsy through Activation of Bcl-2 Family and P38 MAPK Pathways. ACS Chem. Neurosci..

[405] Korczowska-Łącka I., Słowikowski B., Piekut T., Hurła M., Banaszek N., Szymanowicz O., Jagodziński P.P., Kozubski W., Permoda-Pachuta A., Dorszewska J. (2023). Disorders of endogenous and exogenous antioxidants in neurological diseases. Antioxidants.

[406] Borowicz-Reutt K.K., Czuczwar S.J. (2020). Role of oxidative stress in epileptogenesis and potential implications for therapy. Pharmacol. Rep..

[407] Amaghnouje A., Chebaibi M., Aldossari S.M., Ghneim H.K., Amrati F.E.-Z., Es-Safi I., Di Cristo F., Calarco A., Achour S., Carta F. (2023). Origanum majorana L. polyphenols: In vivo antiepileptic effect, in silico evaluation of their bioavailability, and interaction with the NMDA receptor. Front. Chem..

[408] Ahmed H., Khan M.A., Ali Zaidi S.A., Muhammad S. (2021). In silico and in vivo: Evaluating the therapeutic potential of kaempferol, quercetin, and catechin to treat chronic epilepsy in a rat model. Front. Bioeng. Biotechnol..

[409] Xie R., Zhao W., Lowe S., Bentley R., Hu G., Mei H., Jiang X., Sun C., Wu Y., Liu Y. (2022). Quercetin alleviates kainic acid-induced seizure by inhibiting the Nrf2-mediated ferroptosis pathway. Free Radic. Biol. Med..

[410] Liao Z.-J., Liang R.-S., Shi S.-S., Wang C.-H., Yang W.-Z. (2016). Effect of baicalin on hippocampal damage in kainic acid-induced epileptic mice. Exp. Ther. Med..

[411] Machado K.C., Oliveira G.L.S., Islam T., Junior A.L.G., De Sousa D.P., Freitas R.M. (2015). Anticonvulsant and behavioral effects observed in mice following treatment with an ester derivative of ferulic acid: Isopentyl ferulate. Chem. Biol. Interact..

[412] Singh T., Kaur T., Goel R.K. (2017). Ferulic acid supplementation for management of depression in epilepsy. Neurochem. Res..

[413] Li G., Huang L., Gu D., Wang P., Yi L., Kuang W., Zhang Y., Zhang J., Liu D., Shi Q. (2025). Activity-based chemical proteomics reveals caffeic acid ameliorates pentylenetetrazol-induced seizures by covalently targeting aconitate decarboxylase 1. Cell Commun. Signal..

[414] Dhir A. (2020). Natural polyphenols in preclinical models of epilepsy. Phytother. Res..

[415] Nieoczym D., Socała K., Jedziniak P., Wyska E., Wlaź P. (2019). Effect of Pterostilbene, a Natural Analog of Resveratrol, on the Activity of some Antiepileptic Drugs in the Acute Seizure Tests in Mice. Neurotox. Res..

[416] Qu Z., Jia L., Xie T., Zhen J., Si P., Cui Z., Xue Y., Sun C., Wang W. (2019). (-)-Epigallocatechin-3-Gallate Protects Against Lithium-Pilocarpine-Induced Epilepsy by Inhibiting the Toll-Like Receptor 4 (TLR4)/Nuclear Factor-κB (NF-κB) Signaling Pathway. Med. Sci. Monit..

[417] Cano A., Ettcheto M., Espina M., Auladell C., Calpena A.C., Folch J., Barenys M., Sánchez-López E., Camins A., García M.L. (2018). Epigallocatechin-3-gallate loaded PEGylated-PLGA nanoparticles: A new anti-seizure strategy for temporal lobe epilepsy. Nanomedicine.

[418] Mustapha S., Magaji R.A., Magaji M.G., Gaya I.B., Umar B., Yusha’u Y., Daku A.B., Chiroma S.M., Jaafar A., Mehat M.Z. (2024). Neuroprotective roles of flavonoid “hispidulin” in the central nervous system: A review. Iran. J. Basic Med. Sci..

[419] Kavvadias D., Sand P., Youdim K.A., Qaiser M.Z., Rice-Evans C., Baur R., Sigel E., Rausch W., Riederer P., Schreier P. (2004). The flavone hispidulin, a benzodiazepine receptor ligand with positive allosteric properties, traverses the blood-brain barrier and exhibits anticonvulsive effects. Br. J. Pharmacol..

[420] Agregán R., Munekata P.E.S., Franco D., Dominguez R., Carballo J., Lorenzo J.M. (2017). Phenolic compounds from three brown seaweed species using LC-DAD-ESI-MS/MS. Food Res. Int..

[421] Cheng Y., Zhang Y., Huang P., Cheng Q., Ding H. (2024). Luteolin ameliorates pentetrazole-induced seizures through the inhibition of the TLR4/NF-κB signaling pathway. Epilepsy Res..

[422] Socała K., Kowalczuk-Vasilev E., Komar M., Szalak R., Wyska E., Wlaź P. (2025). Effect of apigenin on seizure susceptibility, parvalbumin immunoreactivity, and GABAA receptor expression in the hippocampal neurons in mice. Eur. J. Pharmacol..

[423] Li B., Yan Y., Zhang T., Xu H., Wu X., Yao G., Li X., Yan C., Wu L.-L. (2024). Quercetin reshapes gut microbiota homeostasis and modulates brain metabolic profile to regulate depression-like behaviors induced by CUMS in rats. Front. Pharmacol..

[424] El Shoubaky G.A., Abdel-Daim M.M., Mansour M.H., Salem E.A. (2016). Isolation and Identification of a Flavone Apigenin from Marine Red Alga Acanthophora spicifera with Antinociceptive and Anti-Inflammatory Activities. J. Exp. Neurosci..

[425] Xia Y., Peng S., Lin M., Duan H., Yuan F., Shao M., Tan W., Luo H. (2023). Apigenin attenuates visceral hypersensitivity in water avoidance stress rats by modulating the microbiota-gut-brain axis and inhibiting mast cell activation. Biomed. Pharmacother..

[426] Arora N., Philippidis G.P. (2023). The Prospects of Algae-Derived Vitamins and Their Precursors for Sustainable Cosmeceuticals. Processes.

[427] Watanabe F., Yabuta Y., Bito T., Teng F. (2014). Vitamin B_12_-containing plant food sources for vegetarians. Nutrients.

[428] Leong Y.K., Chang J.-S. (2024). Proteins and bioactive peptides from algae: Insights into antioxidant, anti-hypertensive, anti-diabetic and anti-cancer activities. Trends Food Sci. Technol..

[429] Diler Durgut B. (2025). Neurologic disease and vitamin B12 levels in children. J. Child Neurol..

[430] Liu Q., Yu D. (2025). Interaction and association between multiple vitamins and social adaptability and severity of autism: A large-scale retrospective study from China. Autism Res..

[431] Zou M., Zhang Y., Li D., Li S., Hu J., Gao Y., Cheng Z., Liu S., Wu L., Sun C. (2024). Correlation of Co-Morbidities with Symptom Severity of Children with Autism Spectrum Disorder: A Cross-Sectional Survey. Nutrients.

[432] Zheng L., Jiao Y., Zhong H., Tan Y., Yin Y., Liu Y., Liu D., Wu M., Wang G., Huang J. (2024). Human-derived fecal microbiota transplantation alleviates social deficits of the BTBR mouse model of autism through a potential mechanism involving vitamin B6 metabolism. mSystems.

[433] Parra M., Stahl S., Hellmann H. (2018). Vitamin B_6_ and its role in cell metabolism and physiology. Cells.

[434] Wang N., Zhao Y., Gao J. (2021). Association Between Peripheral Blood Levels of Vitamin A and Autism Spectrum Disorder in Children: A Meta-Analysis. Front. Psychiatry.

[435] Liu Z., Wang J., Xu Q., Wu Z., You L., Hong Q., Zhu J., Chi X. (2022). Vitamin A supplementation ameliorates prenatal valproic acid-induced autism-like behaviors in rats. Neurotoxicology.

[436] Tian P., Zhu X., Liu Z., Bian B., Jia F., Dou L., Jie Y., Lv X., Zhao T., Li D. (2025). Effects of vitamin D on brain function in preschool children with autism spectrum disorder: A resting-state functional MRI study. BMC Psychiatry.

[437] Adibsaber F., Ansari S., Elmieh A., Barkadehi B. (2024). Vitamin D3 Supplementation and Aquatic Exercise Combination as a Safe- Efficient Therapeutic Strategy to Ameliorate Interleukin-6 and 10, and Social Interaction in Children with Autism. Iran. J. Child Neurol..

[438] Gao T., Dang W., Jiang Z., Jiang Y. (2024). Exploring the Missing link between vitamin D and autism spectrum disorder: Scientific evidence and new perspectives. Heliyon.

[439] Mobarakeh K.A., Mahmoudi Z., Mousavi Z., Kachooei M.A., Adabi S.B., Nabi S.B.S., Moradi M., Saeedirad Z., Mohammadi S., Yazdi S.A.N. (2025). Autism spectrum disorder and dietary intake of vitamin E. Neuropsychopharmacol. Rep..

[440] Zhang L., Ji J., Wang Y., Wang L., Zheng R., Jiang Y. (2024). Plasma vitamin levels and pathway analysis in boys with autism spectrum disorders. Sci. Rep..

[441] Alharbi A., Bamaga A.K., Algarni M.A., Abduljabbar M.H., Alnemari R.M., Althobaiti Y.S., Alsenani F., Abdelazim A.H., Almalki A.H. (2024). Spectrofluorometric determination of ascorbic acid in the plasma matrix: Exploring correlation with autism spectrum disorder. Anal. Biochem..

[442] van Heerden C., Cheng D.R., McNab S., Burgess R., Russell A., Wang Y., Bleathman F., Maharaj I., Zhang J., Easterbrook M. (2024). Scurvy and vitamin C deficiency in an Australian tertiary children’s hospital. J. Paediatr. Child Health.

[443] Margedari P., Goudarzi I., Sepehri H. (2024). The protective role of prenatal administration of ascorbic acid on autistic-like behavior in a rat model of autism. IBRO Neurosci. Rep..

[444] Alabdali A.N., Ben Bacha A., Alonazi M., Abuaish S., Almotairi A., Al-Ayadhi L., El-Ansary A.K. (2025). Impact of GABA and nutritional supplements on neurochemical biomarkers in autism: A PPA rodent model study. Front. Mol. Neurosci..

[445] Tarento T.D.C., McClure D.D., Talbot A.M., Regtop H.L., Biffin J.R., Valtchev P., Dehghani F., Kavanagh J.M. (2019). A potential biotechnological process for the sustainable production of vitamin K1. Crit. Rev. Biotechnol..

[446] Tamadon-Nejad S., Ouliass B., Rochford J., Ferland G. (2018). Vitamin K deficiency induced by warfarin is associated with cognitive and behavioral perturbations, and alterations in brain sphingolipids in rats. Front. Aging Neurosci..

[447] Li J., Lin J.C., Wang H., Peterson J.W., Furie B.C., Furie B., Booth S.L., Volpe J.J., Rosenberg P.A. (2003). Novel role of vitamin k in preventing oxidative injury to developing oligodendrocytes and neurons. J. Neurosci..

[448] De Roeck-Holtzhauer Y., Quere I., Claire C. (1991). Vitamin analysis of five planktonic microalgae and one macroalga. J. Appl. Phycol..

[449] Dong H., Wang B., Feng J., Yue X., Jia F. (2021). Correlation Between Serum Concentrations of Menaquinone-4 and Developmental Quotients in Children With Autism Spectrum Disorder. Front. Nutr..

[450] Graham S.F., Turkoglu O., Yilmaz A., Ustun I., Ugur Z., Bjorndhal T., Han B., Mandal R., Wishart D., Bahado-Singh R.O. (2020). Targeted metabolomics highlights perturbed metabolism in the brain of autism spectrum disorder sufferers. Metabolomics.

[451] Gulati S., Narayan C.L., Mahesan A., Kamila G., Kapoor S., Chaturvedi P.K., Scaria V., Velpandian T., Jauhari P., Chakrabarty B. (2026). Transmethylation and Oxidative Biomarkers in Children with Autism Spectrum Disorder: A Cross Sectional Study. J. Autism Dev. Disord..

[452] Williams P.G., Sears L., Watson W.H., Gunaratnam B., Feygin Y., Wright S.P., Sullivan J.E. (2025). Glutathione, Vitamin C, and Cysteine Use in Autistic Children With Disruptive Behavior: A Double-Blind, Placebo-Controlled Crossover Pilot Study. J. Dev. Behav. Pediatr..

[453] Bragg M.G., Rando J., Carroll K.N., Eick S.M., Karagas M.R., Lin P.-I., Schmidt R.J., Lyall K. (2025). The Association of Prenatal Dietary Factors with Child Autism Diagnosis and Autism-Related Traits Using a Mixtures Approach: Results from the Environmental Influences on Child Health Outcomes Cohort. J. Nutr..

[454] Naghavi E., Davari A., Bahadori A.R., Razmafrooz M., AmiriFard H., Sabzgolin I., Tafakhori A., Sheikhvatan M., Ranji S. (2024). Late-onset drug-resistant epilepsy in pyridoxamine 5′-phosphate oxidase deficiency: A case report. J. Med. Case Rep..

[455] Del Mondo A., Smerilli A., Sané E., Sansone C., Brunet C. (2020). Challenging microalgal vitamins for human health. Microb. Cell Fact..

[456] Bilge S., Taşkın S.N. (2025). Vitamin D status of pediatric epilepsy patients and evaluation of affecting factors. Ital. J. Pediatr..

[457] Bashiri F.A., Hudairi A., Hamad M.H., Al-Sulimani L.K., Al Homyani D., Al Saqabi D., Kentab A.Y., Al Khalifah R.A. (2024). Vitamin D Supplementation for Children with Epilepsy on Antiseizure Medications: A Randomized Controlled Trial. Children.

[458] Cheng Z., Zuo J., Peng X., Zhang H., Su W., Luan G., Guan Y. (2024). Causal Relationships Between Epilepsy, Anti-Epileptic Drugs, and Serum Vitamin D and Vitamin D Binding Protein: A Bidirectional and Drug Target Mendelian Randomization Study. CNS Neurosci. Ther..

[459] Chen H., Zheng Z., Cai X., Gao F. (2024). Causal links between serum micronutrients and epilepsy: A Mendelian randomization analysis. Front. Neurol..

[460] Chassoux F., Navarro V., Quirins M., Laurent A., Gavaret M., Cousyn L., Crépon B., Landré E., Marchi A., Soufflet C. (2024). Vitamin D deficiency and effect of treatment on seizure frequency and quality of life parameters in patients with drug-resistant epilepsy: A randomized clinical trial. Epilepsia.

[461] Li Y., Liu X., Sun X., Li H., Wang S., Tian W., Xiang C., Zhang X., Zheng J., Wang H. (2024). Gut dysbiosis impairs intestinal renewal and lipid absorption in Scarb2 deficiency-associated neurodegeneration. Protein Cell.

[462] Ismy J., Soebadi A., Mangunatmadja I., Monica M., Sari T.T., Yuliarti K. (2024). Role of antioxidants in reducing oxidative stress and seizure frequency in drug-resistant epileptic patients. Narra J..

[463] Wu J., Qin D., Liang Z., Liu Q., Wang M., Guo Y., Guo W. (2025). Dysregulation of astrocyte-derived matrix gla protein impairs dendritic spine development in pyridoxine-dependent epilepsy. Mol. Ther..

[464] Tarahomi P., Arab M., Seyedinia S.A., Rahmani M., Rashidy-Pour A., Vafaei A.A., Raise-Abdullahi P. (2025). Vitamin C and Gallic Acid Ameliorate Motor Dysfunction, Cognitive Deficits, and Brain Oxidative Stress in a Valproic Acid-Induced Model of Autism. Brain Behav..

[465] Chatterjee K., Mazumder P.M., Sarkar S.R., Saha R., Chatterjee A., Sarkar B., Banerjee S. (2023). Neuroprotective effect of Vitamin K2 against gut dysbiosis associated cognitive decline. Physiol. Behav..

[466] Sim M., Hong S., Jung M.H., Choi E.Y., Hwang G.-S., Shin D.-M., Kim C.-S. (2025). Gut microbiota links vitamin C supplementation to enhanced mental vitality in healthy young adults with suboptimal vitamin C status: A randomized, double-blind, placebo-controlled trial. Brain Behav. Immun..

[467] Biyong E.F., Alfos S., Dumetz F., Helbling J.-C., Aubert A., Brossaud J., Foury A., Moisan M.-P., Layé S., Richard E. (2021). Dietary vitamin A supplementation prevents early obesogenic diet-induced microbiota, neuronal and cognitive alterations. Int. J. Obes..

[468] Chen B.-W., Zhang K.-W., Chen S.-J., Yang C., Li P.-G. (2021). Vitamin A deficiency exacerbates gut microbiota dysbiosis and cognitive deficits in amyloid precursor protein/presenilin 1 transgenic mice. Front. Aging Neurosci..

[469] Zali A., Hajyani S., Salari M., Tajabadi-Ebrahimi M., Mortazavian A.M., Pakpour B. (2024). Co-administration of probiotics and vitamin D reduced disease severity and complications in patients with Parkinson’s disease: A randomized controlled clinical trial. Psychopharmacology.

[470] Cao G., Yu Y., Wang H., Yang H., Tao F., Yang S., Liu J., Li Z., Yang C. (2024). Dietary Clostridium butyricum and 25-Hydroxyvitamin D3 modulate bone metabolism of broilers through the gut-brain axis. Poult. Sci..

[471] Lu Y., Lin M., Ou S., Sun L., Qian K., Kuang H., Wu Y. (2024). Astragalus polysaccharides ameliorate epileptogenesis, cognitive impairment, and neuroinflammation in a pentylenetetrazole-induced kindling mouse model. Front. Pharmacol..

[472] Nonato D.T.T., Aragão G.F., Craveiro R.M.C.B., Pereira M.G., Vasconcelos S.M.M., Wong D.V.T., Júnior R.C.P.L., Soares P.M.G., Lima M.A.d.S., Assreuy A.M.S. (2024). Polysaccharide-rich extract of Genipa americana leaves protects seizures and oxidative stress in the mice model of pentylenetetrazole-induced epilepsy. Biomed. Pharmacother..

[473] Yakmaz F., Bozkurt A.S., Görücü Yilmaz Ş. (2024). PTZ-kindled rat model; evaluation of seizure, hippocampal EGR-1, and Rev-erbα gene regulation, behavioral analysis, and antioxidant capacity of Gum Arabic. Mol. Biol. Rep..

[474] Wang D., Hu B., Hu C., Zhu F., Liu X., Zhang J., Wang B., Xiang H., Cheng Z., Xiong Y. (2020). Clinical Characteristics of 138 Hospitalized Patients with 2019 Novel Coronavirus-Infected Pneumonia in Wuhan, China. JAMA.

[475] Anyanwu G.E., Nwachukwu I.J., Oria S.R., Obasi K.K., Ekwueme E.P., Nto J.N., Anyanwu N.C. (2024). Fisetin attenuates AlCl3-induced neurodegeneration by modulating oxidative stress and inflammatory cytokine release in adult albino wistar rats. Toxicol. Rep..

[476] Trovò L., Fuchs C., De Rosa R., Barbiero I., Tramarin M., Ciani E., Rusconi L., Kilstrup-Nielsen C. (2020). The green tea polyphenol epigallocatechin-3-gallate (EGCG) restores CDKL5-dependent synaptic defects in vitro and in vivo. Neurobiol. Dis..

[477] Duarte R.M.F., Ribeiro-Barbosa E.R., Ferreira F.R., Espindola F.S., Spini V.B.M.G. (2025). Resveratrol prevents offspring’s behavioral impairment associated with immunogenic stress during pregnancy. Prog. Neuropsychopharmacol. Biol. Psychiatry.

[478] Nieoczym D., Socała K., Zelek-Molik A., Pieróg M., Przejczowska-Pomierny K., Szafarz M., Wyska E., Nalepa I., Wlaź P. (2021). Anticonvulsant effect of pterostilbene and its influence on the anxiety- and depression-like behavior in the pentetrazol-kindled mice: Behavioral, biochemical, and molecular studies. Psychopharmacology.

[479] Dang J., Paudel Y.N., Yang X., Ren Q., Zhang S., Ji X., Liu K., Jin M. (2021). Schaftoside Suppresses Pentylenetetrazol-Induced Seizures in Zebrafish via Suppressing Apoptosis, Modulating Inflammation, and Oxidative Stress. ACS Chem. Neurosci..

